# Health status, health behavior and perceived stress of nursing staff in Germany: a scoping review

**DOI:** 10.1186/s12912-025-04282-4

**Published:** 2026-01-09

**Authors:** Stefanie Pecha, Ralph Brinks, Insa Feinkohl, Christine Macare, Ozlem Koseoglu-Ornek

**Affiliations:** 1 Department of Human Medicine, Medical Biometrics and Epidemiology, Faculty of Health, Witten/Herdecke University, Alfred-Herrhausen-Straße 50, 58448 Witten, Germany; 2 Epidemiology Research Group, Faculty of Health Sciences Brandenburg, University of Potsdam, Potsdam, Germany; 3 Max Delbrück Center for Molecular Medicine in the Helmholtz Association (MDC), Berlin, Germany; 4 Nursing Department, Faculty of Health, Witten/Herdecke University, Witten, Germany

**Keywords:** Evidence gaps, Nursing profession, Well-being, Job demands, Health promotion

## Abstract

**Objective:**

In view of increasing work-related burdens resulting from staff shortages, demographic changes, and high physical and psychological demands, there is a growing need for an understanding of the health status of nursing staff in Germany. The aim of this review is to consolidate existing knowledge on nurses’ health, health behaviors, and subjective stress perceptions to highlight existing research gaps, and to provide impetus for the development of future health-promoting interventions.

**Methods:**

To analyze the research field, a scoping review was conducted following the JBI methodology. The systematic literature search was carried out using CINAHL, PubMed, and CareLit- databases and was supplemented by searches of the preprint servers OpenGrey and MedRxiv. In addition, a targeted supplementary search for relevant publications was also conducted on selected pertinent websites.

**Results:**

A total of 11,006 titles and abstracts were screened, of which 150 full texts were reviewed, resulting in the inclusion of 90 studies. The literature predominantly focused on nurses’ mental health. Physical health and health-related behaviors were examined less frequently. Results consistently indicate a high burden of morbidity and substantial work-related stress, which have significant implications for individual well-being, professional performance, and long-term retention in the nursing profession. These challenges have been further intensified by the COVID-19 pandemic. Protective factors such as team cohesion and recognition have emerged repeatedly, highlighting the importance of supportive work environments. Although some interventions have demonstrated short-term improvements in mental health outcomes, robust evidence of long-term effects and physical health promotion remains limited.

**Conclusions:**

Nursing staff are exposed to a wide range of health risks and high work-related burdens. Despite a broad body of research, substantial gaps remain - particularly regarding health behaviors and physical health. Future research requires longitudinal, comparative studies, and a structured, nursing-specific health monitoring system. In practice, comprehensive strategies that combine individual-level interventions with structural improvements in the work environment are needed.

**Review registration:**

Open Science Framework 10.17605/OSF.IO/HX9ZM.

**Clinical trial number:**

Not applicable.

**Supplementary Information:**

The online version contains supplementary material available at 10.1186/s12912-025-04282-4.

## Introduction

### Background and rationale

As of 2023, approximately 1.7 million professionals have worked in the field of nursing and caregiving [1]. These professionals are responsible for providing care to approximately 5 million individuals in need [2] and operate within an increasingly demanding work environment shaped by profound societal, demographic, and technological changes [3]. Rising life expectancy and the associated increase in care dependency have significantly heightened the demand for qualified nursing staff and are expected to drive further growth in the future [4, 5]. Moreover, the number of active nursing professionals remains limited due to demographic changes, the perceived low attractiveness of the profession, and high physical and psychological demands [6, 7]. These conditions have resulted in considerable workload intensification and a corresponding increase in physical and psychological strain within the nursing profession [3, 8, 9].

Various work-related stress factors affect nurses’ lives in multiple ways. These factors include dealing with death and dying; resistance from care recipients during the implementation of nursing measures; emotional conflicts between nursing staff and family members; unclear information flows; high workload; poor management practices, such as unfair treatment; lack of social support; staff shortages; long and irregular working hours; physical demands; conflicts with colleagues or other professional groups; and insufficient training opportunities. Additional factors include a lack of appreciation, perceived inadequate pay, unfavorable working hours, and time pressure, which make balancing work and family life more difficult [10–19]. Climatic conditions have also been cited as an additional stress factor, particularly in home care [20].

Work-related stress is a significant issue with far-reaching effects on the health, safety, and well-being of nursing staff, as emphasized by the World Health Organization and other leading institutions in occupational health. These organizations play pivotal roles in policy development and conceptualize work-related stress from various perspectives [21–26]. For example, the World Health Organization defines work-related stress as situations in which work demands exceed the knowledge and skills of nursing staff and challenge their coping capacities [21]. The National Institute for Occupational Safety and Health (NIOSH), on the other hand, describes work-related stress as negative physical and emotional reactions that occur when job demands do not match employees’ abilities, resources, or needs [22].

Prolonged exposure to this work-related stress may result in multidimensional health issues among nurses, including physical health problems such as musculoskeletal disorders; mental health conditions such as depression and anxiety; and sleep disturbances and burnout, which are often driven by the high physical and psychological demands of nursing work [3, 9, 27–29]. Studies conducted in Germany further highlight the particular vulnerability of nurses to health issues, as demonstrated by elevated sickness absence, widespread work-related illnesses, and an increase in early retirement rates [3, 8, 30]. In addition to affecting nurses’ health, these stressors also compromise the quality of patient care [28, 31]. Approximately 46% of nursing professionals report that they (frequently or very frequently) manage their workload at the expense of the quality of their work [28]. This simultaneously leads to reduced empathy toward care recipients among nurses, a decline in the quality of effective communication, and an increase in professional errors [32]. Relieving the burden on nursing staff is crucial not only for their own health but also for the stability and functionality of the healthcare system [28, 33].

Individual differences in stress perception and coping strategies are well documented. Health behavior, such as avoiding smoking and alcohol, regular physical activity, effective stress management, balanced and healthy nutrition, adequate and restorative sleep, taking responsibility for one’s health, maintaining healthy interpersonal relationships, and spiritual development, plays a dynamic and multidimensional role in shaping these differences, functioning in terms of both causes and consequences [34]. For example, nurses who work irregular and extended hours in shift-based systems often face limitations in sustaining health behaviors such as regular exercise, healthy eating, and sufficient sleep. Therefore, health behaviors are influenced not only by individuals’ life philosophies or health literacy but also by the resources and conditions available to them, including their working environment [35].

The described challenges clearly demonstrate the urgent need for measures to sustainably improve working conditions in nursing. However, a solid foundation for such measures can be established only if the scientific data are precise and up-to-date. Nevertheless, a more in-depth analysis of the previously cited studies underscores the existence of substantial research gaps. Some of the studies cited are based on older data [11, 14, 16, 18, 36], which may no longer reflect the current challenges in the nursing profession. Others rely on more recent data but are limited to specific regions of Germany or particular specialties and/or have small sample sizes [9, 10, 17, 20]. Additionally, the studies by Kirmse et al. [19] and Hower et al. [15] were conducted during an exceptional period, shortly after or during the COVID-19 lockdown, which likely influenced the results because of altered working conditions and increased burdens. These limitations minimize the generalizability of findings to the broader nursing population. Furthermore, individual insights are often fragmented and focus on specific health aspects, making a comprehensive analysis of the overall health situation and its causes and impacts challenging.

A scoping review was subsequently identified as the most appropriate method of evidence synthesis for this analysis. Scoping reviews helps clarify concepts, identify knowledge gaps, and evaluate the utility of further research efforts [37]. The primary objective of a scoping review is to collect and summarize relevant evidence on a specific phenomenon of interest, allowing for the examination of a wide range of evidence [38]. Although the methodology typically does not include a critical appraisal of the quality of the included evidence [38], it still requires a thoughtful interpretation of the findings and an informed discussion about their relevance to the review’s objectives and future research [37–40].

An initial search in MEDLINE (PubMed), the Cochrane Database of Systematic Reviews, and JBI (Joanna Briggs Institute) Evidence Synthesis was conducted prior to commencing the scoping review. The results indicated that existing reviews either focus on specific aspects or on particular professional groups within nursing, without providing a holistic picture of the situation [41–44].

The aim of this scoping review was to capture and systematically present the current evidence base to gain a comprehensive understanding of the health situation of nursing staff in Germany. As this review conceptualizes health as a multidimensional construct encompassing physical, mental and behavioral aspects shaped by reciprocal interactions, three overarching objectives were defined:

- To describe the health status of nursing staff in Germany.
- To describe the health behavior of nurses in Germany.
- To describe the perceived work-related stress among nursing staff in Germany.

Conducting this scoping review is particularly important, as it will provide a foundation for developing targeted health promotion and prevention measures within the nursing profession. Furthermore, providing evidence-based insights into the conditions necessary for a healthy and sustainable work environment will contribute to enhancing the long-term attractiveness of nursing. Against this backdrop, this scoping review will not only offer an overview of the literature but also derive practical recommendations and guide future research aimed at promoting and preserving the health and well-being of nursing professionals.

### Key questions of the scoping review

The following questions were key to achieving the aim of the scoping review:

- What empirical surveys on the state of health, health behavior and work-related stress of nurses have been conducted in Germany?
- What scientific findings on the state of health, health behavior and work-related stress of nurses exist for Germany to date?

### Eligibility criteria

The eligibility criteria of the included studies were described on the basis of the Population, Concept, and Context (PCC) framework [45].

#### Population

Sources of evidence describing the role and scope of professional nursing caregivers, including nursing assistants, were considered in this review regardless of their origin or gender. Informal caregivers, such as family members, as well as professionals from related healthcare fields such as medical assistants, midwives and physicians were excluded, as the roles and scopes of these practitioners were not the focus of this review. However, publications addressing multiple healthcare professions, including nursing, were considered if they were conceptually relevant and allowed for the extraction of nursing-specific findings; data extraction focused exclusively on the nursing profession.

#### Concept

This scoping review examines the health, health behavior, and work-related stress of nursing staff. Health and health behavior, as well as the effects of subjectively perceived stress, are shaped and mediated by individual processes and social interactions. The interactions between these constructs are characterized by dynamic, bidirectional feedback loops. A high prevalence of illness can reduce functional capacity and deplete personal resources, thereby intensifying perceived strain. Conversely, strongly perceived work demands may lead to overload, exhaustion, or behavioral adaptations. Such adaptations of lifestyle can in turn promote or exacerbate health problems [34, 46, 47]. These reciprocal processes illustrate the close and dynamic interplay between health status, health behavior, and work-related stress among nursing staff. Due to these interactions, overlaps between the individual dimensions are to be expected, making an integrated analytical perspective essential for this review.

Accordingly, the conceptual framework of this review is regarded as a complex and multilayered construct that can be measured and operationalized via scientific health indicators. These indicators provide insights into health status, health-related behaviors, healthcare utilization, and available resources within a defined population group [48].

The selection of health indicators was guided by the established population-based health survey GEDA (German Health Update) conducted by the Robert Koch Institute [49]. Indicators such as self-rated health status, the presence of mental illnesses, and the prevalence of chronic physical conditions and complaints were included to allow for a differentiated assessment of both subjective and objective aspects of health [49].

To adequately capture health-related behaviors, the risk factors according to the SNAP guidelines (smoking, nutrition, alcohol consumption, physical activity) were considered [50]. These are considered key determinants of health, as they are closely linked to the development of chronic diseases [51].

**Fig. 1:**
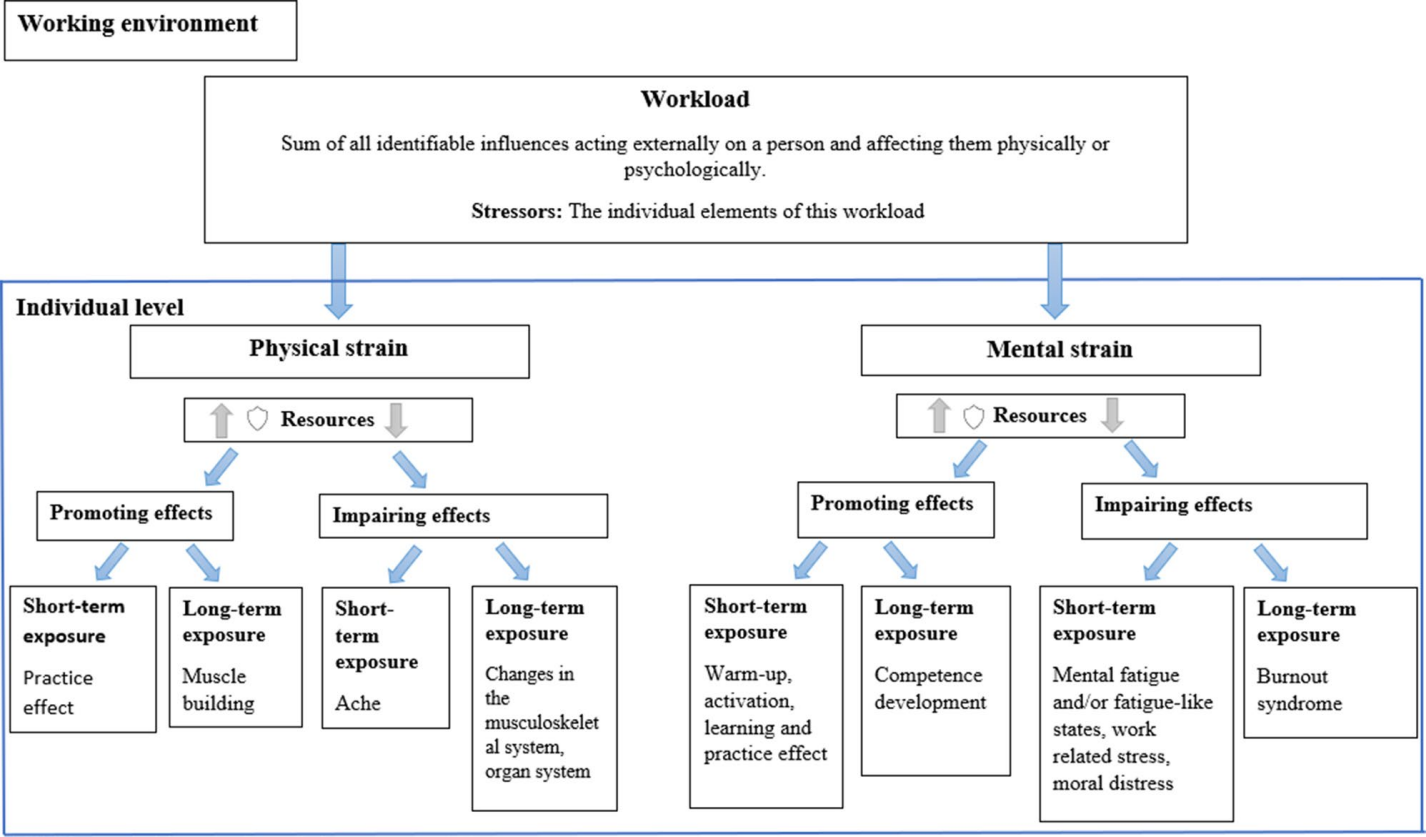
Conceptual interrelations of terminology (own illustration based on [34]). The figure illustrates the interrelations between workload, strain, and their psychological consequences, including stress, burnout, and moral distress. The model distinguishes external work demands from individual responses and depicts how short- and long-term effects emerge

Work-related stress was operationalized on the basis of search terms derived from a systematic review on psychological strain and occupational stress in the healthcare sector [52]. This approach was deliberately expanded to comprehensively capture the multidimensional nature of work-related demands and their potential implications for nursing staff’s health. To further clarify the conceptual framework, Fig. 1 illustrates the theoretical differentiation of key, partially interrelated terms.

#### Context

As this scoping review specifically investigates nurses in Germany, the contextual framework is defined by geographical boundaries. Consequently, only empirical studies that examine health status, health-related behaviors, and perceived stress among nurses in Germany are included. This geographical limitation is methodologically justified, as the working conditions, healthcare infrastructure, and support systems for nursing professionals in Germany differ substantially from those in other countries [53, 54], limiting the generalizability of international findings. For example, data from the RN4CAST (Nurse Forecasting in Europe) study indicate that nurses in Germany are responsible for an average of 13 patients, whereas in the USA, the average nurse-to-patient ratio is 1:5.3 [53]. These structural differences significantly affect occupational stress, workload, and health status [55].

#### Types of sources

Studies written in English or German with empirical data and reviews from Germany were included. This scoping review considered quantitative, qualitative, and mixed methods study designs for inclusion. In addition, systematic reviews were considered for inclusion in this scoping review. Text and opinion contributions and letters were not considered, as these are often based on subjective views and personal experiences and therefore do not appear suitable for answering the objectives of the scoping review.

## Methods

This scoping review was conducted in accordance with the JBI methodology for scoping reviews [39] and in line with the Preferred Reporting Items for Systematic Reviews and Meta-Analyses extension for Scoping Reviews (PRISMA-ScR) [39]. This manuscript is based on the standardized template of the JBI Evidence Synthesis, which is recommended for the preparation of systematic reviews [56]. Adjustments have been made to consider the specific requirements of this research question. The objectives, inclusion criteria and analysis methods of this review were previously developed in an a-priori protocol. This a-priori protocol was registered with the Oppen Science Framework and published in the journal Praev. Gesundheitsf. in April 2025 [57].

### Search strategy and information sources

The search strategy followed a three-stage process and aimed to identify both published and unpublished primary studies and reviews. An initial limited search of CINAHL (EBSCOhost) and MEDLINE (PubMed) was conducted by SP on 11.12.2024 to identify articles on this topic. The text words contained in the titles and abstracts of relevant articles and the index terms used to describe the articles were used to develop a full search strategy. The search strategy, including all identified keywords and index terms, was adapted to other databases, search engines and sources of gray literature, and was subjected to peer review by another reviewer according to the checklist “Peer Review of Electronic Search Strategies (PRESS)” [58]. This search strategy was further refined based on a pilot phase. In addition to the terms originally defined in the protocol (“nurses OR nursing staff OR nurse”), the terms “nurs* care” and “outpatient care” were included to ensure broader coverage of relevant studies.

In the second search phase, a comprehensive search of all relevant information sources was carried out on 05.03.2025. The databases that were searched included: MEDLINE (PubMed), CINAHL and CareLit. Unpublished primary sources and reviews were searched via OpenGrey (DANS Data Station) of the University of London and medRxiv, a free preprint server for health sciences. Published data from search engines and gray literature sources were considered up to 05.03.2025. The full search strategies are provided in Additional file 1. In deviation from the original scoping review protocol, a targeted supplementary search for relevant publications was also conducted on selected pertinent websites as part of the systematic literature review (see Additional file 2). This deviation was deemed necessary to capture potentially high-value publications from key institutions that may not be indexed in bibliographic databases.

In the third and final step, a randomly selected subset (approximately 10%) of the articles included in the full-text review was screened for references to identify potentially additional studies.

In the initial step, the scoping review included publications from the last ten years (March 2015-March 2025) to reflect the most recent evidence and current practice conditions in nursing care. For the final data extraction, however, only studies whose data collection itself was conducted within the last ten years were included, to ensure that the findings are based on the most up-to-date empirical data available.

### Data collection and study selection

Following the search, all identified records were collated and uploaded to the bibliographic software EndNote 21.4 (Clarivate Analytics, PA, USA) and exported to Covidence (Release May 2022; Veritas Health Innovation, Melbourne, Australia) for study selection management [59]. First, all existing duplicates were removed. All remaining records were screened at both the title/abstract and full-text levels by at least two independent reviewers (SP; CM) based on predefined inclusion criteria and keywords relevant to the review question and objectives. This process was preceded by a pilot test involving approximately 5% of the identified studies. Inter-Reviewer agreement was assessed via Cohen’s kappa, reported separately for title/abstract screening and full-text screening. Any disagreements that arose between the reviewers were resolved through discussion or with a third reviewer (IF). For publications that appeared potentially relevant, a detailed examination of the full texts was carried out, considering the predefined inclusion criteria. Sources that did not fulfill these criteria were removed from the literature management programs and not considered further in the review. The reasons for exclusion are presented in Additional file 3.

### Data extraction

Data extraction from the studies included after full-text screening was performed via a data extraction form adapted from the standardized JBI tool [45] (Additional file 4). The results were initially documented as bullet points. A brief descriptive summary of the individual results was then prepared on this basis. In addition, the identified stressors were assigned to the overarching categories provided by DIN EN ISO 10075-1:2018-01 as a way to organize and present them more clearly and comprehensibly. For practical reasons, the data collection was carried out by one reviewer (SP), with at least 20% of the data being reviewed by another reviewer (IF). If differences of opinion arose, a third reviewer was called in to clarify any differences.

### Data analysis and presentation

The aim of this scoping review was to record the available evidence and present it in a visual and narrative summary. To this end, the entire research process was visualized via a flowchart and described in narrative form. The results are presented in tabular form, with a narrative summary again accompanying the tabular results and describing how the results relate to the objective and the review questions.

## Results

### Source of evidence inclusion

**Fig. 2:**
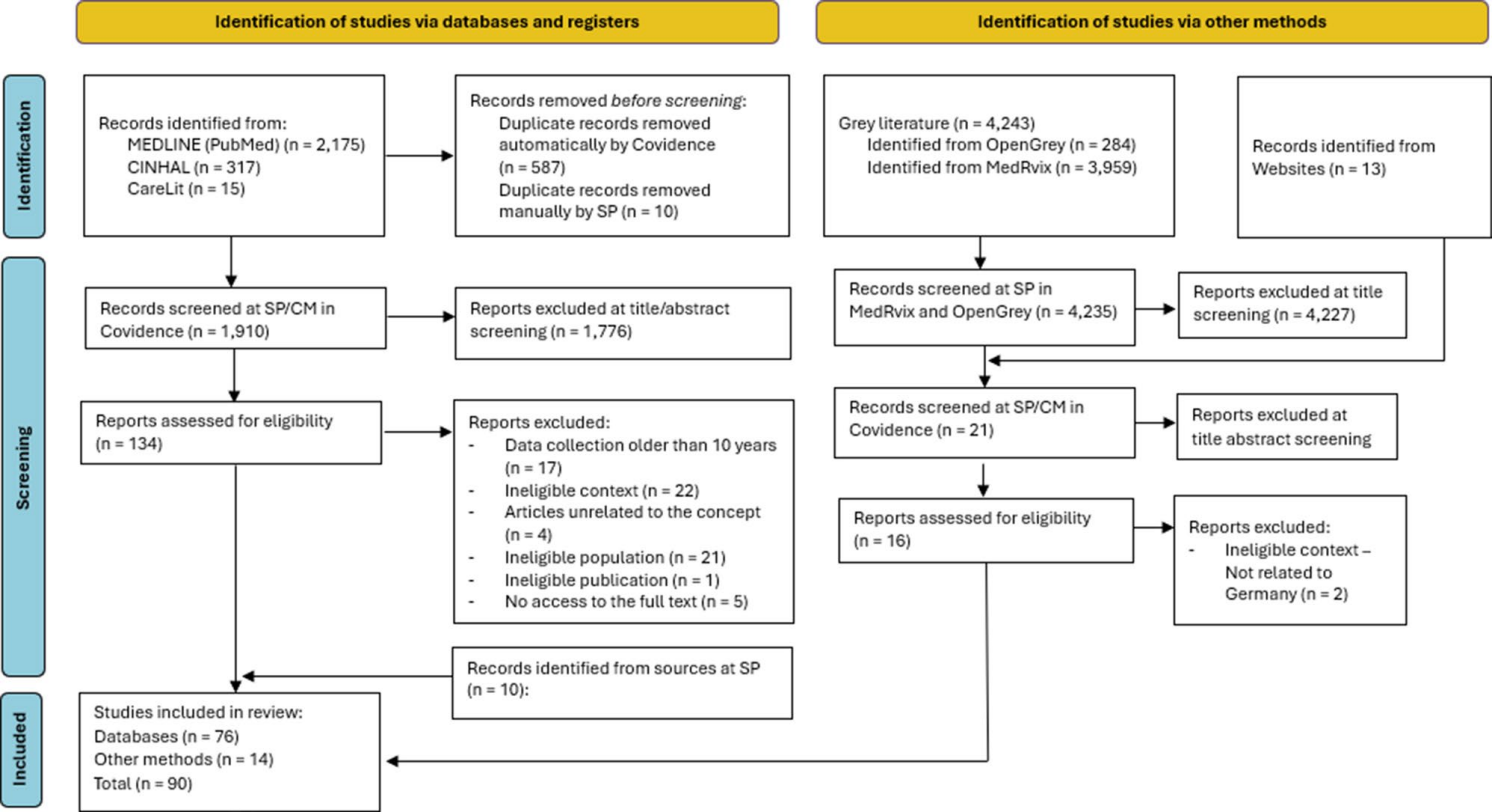
Search results and study selection and inclusion process [60]

The initial search yielded 2,507 titles in the databases MEDLINE (PubMed: 2175), CINAHL (317), and LitCare (15). After removing duplicates, 1,910 titles remained. Additionally, 4,243 entries were identified from preprint servers (medRxiv: 3,959; OpenGrey: 284), along with 13 articles identified through website searches and 10 further studies via cross-references. After reviewing the titles, abstracts, and full texts, a total of 90 studies were included (see Fig. 2). The interrater Cohen’s kappa values for screening titles/abstracts and for screening the full text were 0.623 and 0.947, respectively, indicating substantial and excellent agreement between reviewers, respectively. An overview of the studies excluded after full-text screening, along with the reasons for exclusion, can be found in Additional file 3.

### Characteristics of the included sources

**Fig. 3:**
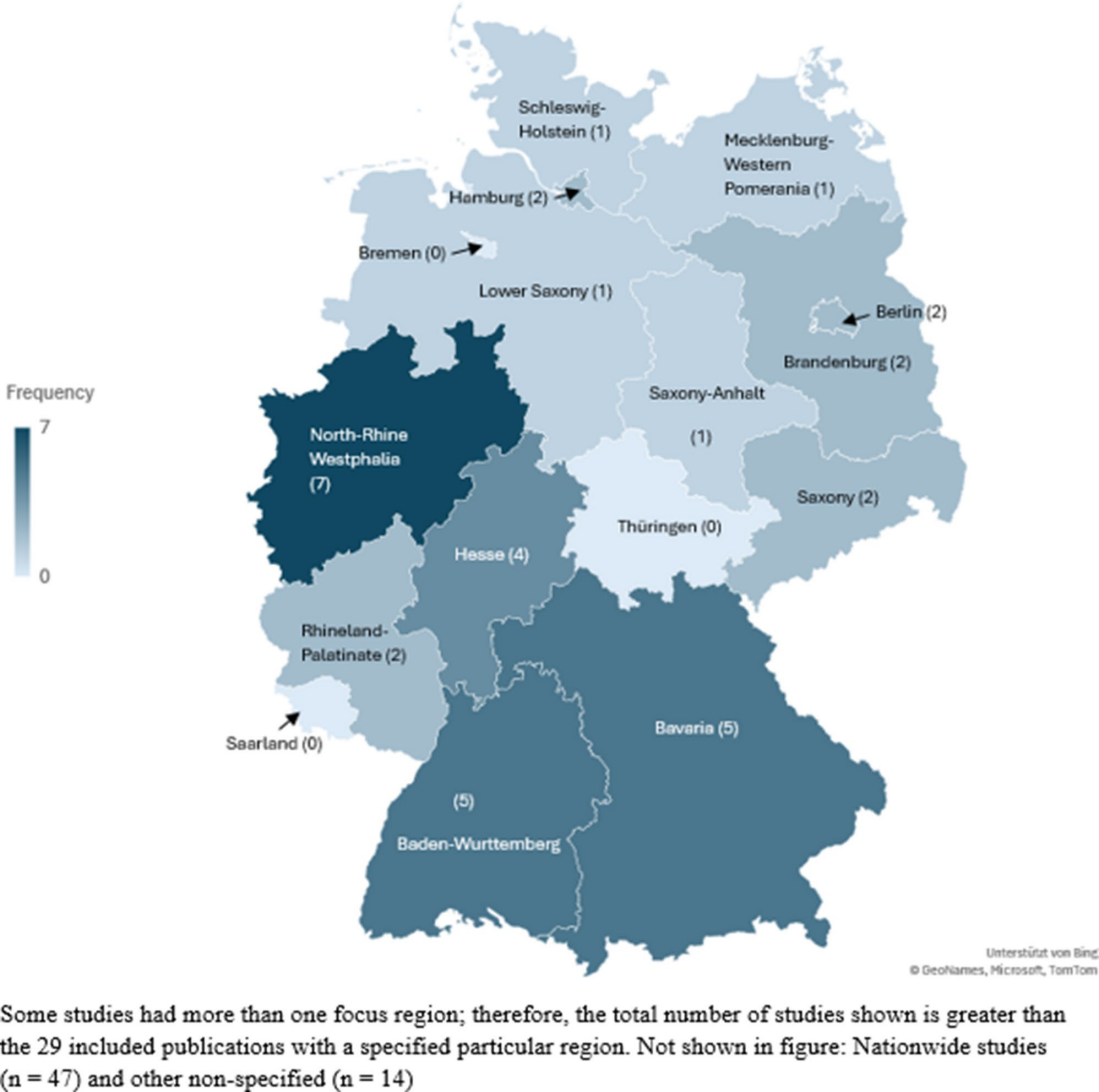
Geographic distribution of the included publications with specified regions

Among the 90 included studies, the majority were primary studies (*n* = 77). Thirteen studies exclusively analyzed secondary data [3, 8, 30, 61–70], and two additional studies used both primary and secondary data [71, 72], resulting in a total of 15 studies involving secondary data analysis. Primary research is understood as studies collecting new, original data, whereas secondary research analyzes data originally collected for other purposes [73]. Among the included studies, 70 were based on quantitative designs, particularly online surveys; eleven were qualitative studies (interview studies); and three followed a mixed-methods design. Additionally, six studies were classified as reviews. Fifty studies followed a setting-specific approach, whereas 40 applied a cross-setting approach. Sixty-six articles provided data on health status, 75 studies focused on workload, and only three studies examined relevant aspects of health behavior. Nearly half (*n* = 40) of the identified studies were related to the COVID-19 pandemic. With respect to geographical focus, 47 studies examined Germany as a whole (nationwide), whereas 43 studies focused on a specific region. However, in 14 of these regionally focused studies, the respective region was not further specified. Study data from studies with a geographical focus (*n* = 29) are displayed in Fig. 3. Studies without a clearly defined region (*n* = 14) were excluded from this figure.

In the primary studies, the sample sizes ranged from 33 to 2,887 participants in the quantitative studies and from 6 to 100 participants in the qualitative studies. The proportion of women ranged between 52% and 93%. Table 1 provides an overview of the characteristics of the included sources.

**Table 1:** Summary of the characteristics of the included evidence sources

Characteristics	References	Number of studies
Study Setting		
Setting-specific	[9, 14–17, 20, 27, 65, 66, 74–114]	50
Cross-setting approach	[3, 8, 10, 13, 19, 30, 62–64, 67–72, 115–139]	40
Geographical Location		
Germany (nationwide)	[3, 8, 9, 13, 15, 16, 19, 27, 30, 61–69, 71, 72, 74–77, 79, 85, 89, 91, 98, 102–104, 106, 107, 109, 110, 116, 119, 123, 128, 130, 131, 133–136, 138]	47
Germany (specifically region)	[10, 14, 17, 20, 70, 78, 80–84, 86–88, 90, 92–97, 99–101, 105, 108, 111–115, 117, 118, 120–122, 125–127, 129, 132, 137, 139]	43
Study Design		
Quantitative design	[3, 8–10, 13–17, 19, 20, 27, 30, 61–64, 71, 72, 74–80, 83, 85, 88–92, 94, 96–100, 102–112, 114–116, 118–123, 125, 127–129, 131, 133–135, 137–139]	70
Qualitative design	[81, 82, 84, 86, 87, 93, 95, 101, 126, 132, 136]	11
Mixed-Methods design	[113, 117, 130]	3
Review	[65–70]	6
Reference to the COVID-19 pandemic		
Without reference to the COVID-19 pandemic	[3, 8, 10, 13, 14, 16, 17, 30, 61–65, 68–72, 74–85, 87–93, 96–98, 115–123, 129]	50
With reference to the COVID-19 pandemic	[9, 15, 19, 20, 27, 66, 67, 94, 95, 98–114, 125–128, 130–139]	40
Related concept*		
State of health	[3, 8–10, 13, 15–17, 19, 20, 27, 30, 61–65, 67–72, 74–77, 83, 85, 86, 88–92, 95–100, 102–105, 107, 108, 110–116, 120–123, 125, 127, 129–131, 134, 135, 139]	66
Mental health**	[3, 8–10, 15, 16, 19, 20, 27, 30, 61–65, 67–72, 74–77, 83, 86, 89–92, 95, 96, 98–100, 102–105, 107, 108, 110–116, 120–123, 125, 127, 129–131, 134, 135, 139]	61
Physical health**	[3, 8–10, 15, 16, 19–21, 24, 55–59, 61–66, 68–71, 77, 80, 83–86, 89, 90, 92–94, 96–99, 101, 102, 104–110, 114–117, 119, 121, 123–125, 128, 129, 133]	27
Health behavior	[71, 104, 130]	3
Work-related stress	[8, 10, 13–17, 19, 20, 30, 61–67, 70–72, 74–84, 86–91, 93, 94, 96, 99–107, 109–119, 121–123, 126, 128–133, 135–139]	75

* Some studies consider health status, work-related stress, and health behavior, which may lead to duplications

** Studies that examined presenteeism or absenteeism were categorized as pertaining to both physical and mental health

A detailed description of the included studies and their results can be found in Additional file 5, which serves as the basis for the subsequent analyses.

### Review findings

#### Health status

Of the 66 included studies examining health status, most focused on mental health (*n* = 61), while fewer addressed physical health (*n* = 27) (see Table 1; multiple entries possible). Mental health was mostly operationalized via established instruments such as the Maslach Burnout Inventory (MBI) [10, 19, 77, 90, 92, 97, 103, 105, 112, 116, 120, 121] or the Copenhagen Psychosocial Questionnaire (COPSOQ) [20, 74–76, 80, 92, 102, 110, 111, 113, 122, 135]. Standardized assessment tools such as the Patient Health Questionnaire (PHQ; 2 to 9 items) [74, 104, 105, 110, 114, 134], the Depression Anxiety and Stress Scale (DASS) [80, 96, 140], and the Copenhagen Burnout Inventory (CBI) [74, 90, 134] were also frequently used.

##### Mental health

Across studies, nursing staff showed high levels of psychological morbidity, particularly mental exhaustion and burnout. Reported burnout prevalence frequently exceeded 40 to 50% across settings, depending on the instrument and cut-off applied [10, 19, 67, 68, 86, 90, 98, 99, 108, 120, 121, 130]. For example, Helaß et al. analyzed data from 83 oncology nurses across Germany and identified burnout in 53% of participants, using a cutoff value of MExh > 2.5 [98]. Depression, anxiety, and related symptoms were also common and consistently reported across studies [3, 13, 19, 71, 72, 74, 83, 104, 110, 129]. Overall, the evidence paints a coherent pattern of substantial psychological burden among nurses.

##### Physical health

Physical morbidity was likewise prevalent. Musculoskeletal disorders were the most frequently documented health problems, with high reported with reported frequencies between 38% and 79% [3, 10, 13, 68, 71, 72, 74, 85, 86, 129]. Cardiovascular diseases were also reported, although with substantial variation between samples, with prevalence estimates ranging from 8% to 39% [13, 129]. Sleep disorders were also repeatedly documented, with rates ranging from 36% [10] to 58% [9], suggesting a possible somatic manifestation of chronic psychological stress. Other relevant somatic symptoms that have been highlighted include digestive issues, headaches [10], and general pain [27]. Analyses of health insurance data further indicated a markedly elevated risk among nurses for chronic diseases such as hypertension, asthma, tobacco dependence, obesity, and type 2 diabetes compared with other occupational groups [71]. Detailed prevalence values are provided in Additional File 5.

##### Setting-specific findings

Setting-specific findings revealed clear differences in health status across nursing sectors: nurses working in outpatient care reported significantly more fear of the future and of failure, higher frustration, and more severe symptoms of exhaustion than those working in inpatient settings did [115]. They also exhibited a higher prevalence of psychosomatic complaints [115]. In contrast, particularly high emotional and physical burdens were observed in inpatient palliative care [83]. On the other hand, despite high work intensity, intensive care nurses reported a lower prevalence of burnout and fewer care omissions possibly due to protective structural factors such as team cohesion or resource availability [131]. Interprofessional comparisons also revealed differences: nurses reported physical complaints such as cardiovascular diseases and obesity, as well as more severe depressive and anxiety symptoms, more frequently than physicians did [100].

##### Consequences of physical and mental health problems among nurses

The reported morbidity rates were also reflected in work-related health indicators such as sickness absence and reduced earning capacity. Average rates of sick leave in nursing professions were higher than those in other occupational groups (7% to 8% vs. approximately 5%, respectively). These percentages refer to the share of employees on sick leave on an average day, calculated as the total number of sick leave days per 100 insured person-years divided by 365 [71]. Similarly, the average number of sickness absence cases (1.38 vs. 1.21) and days (23 vs. 15) per insurance year was likewise significantly higher in the nursing sector, with those working in elderly care being particularly affected [3]. Disability pensions were also more common among nurses: the probability of receiving disability ranged from 4% to 6%, whereas it was approximately 3% for other professions [71]. More recent analyses confirmed this trend, showing that the proportion of early retirements was 6% among nursing staff most recently compared with 4% among non-nursing staff [8, 64].

#### Health behavior

The health behavior of nursing staff has been examined only to a limited extent [71, 104, 130]. According to Rothgang et al. (2020) [71], geriatric care professionals and assistants exhibit a prevalence of tobacco dependence that is more than 20% higher than that of employees in other occupational groups. Furthermore, multiple linear regression analyses by Morawa et al. [104] revealed that higher levels of depressive symptoms are associated with increased alcohol consumption. Heuel et al. (2022) [130] demonstrated that a high level of chronic stress, low self-efficacy expectations, and unfavorable organizational work conditions are associated with generally detrimental health behavior. This includes, among other things, irregular meals, lack of physical activity, and limited use of workplace health promotion programs. Barriers to health-promoting behavior include, in particular, a lack of time, shift work, limited availability and attractiveness of health-related offerings, and individual factors, such as dispositional traits, sleep problems, low levels of social support within the team, dieting behavior, tobacco use, domestic responsibilities, and health-related limitations [130].

#### Work-related stress

Almost all included studies reported high levels of perceived stress among nursing staff, resulting from a complex interaction of organizational, physical, emotional, and interpersonal factors. The qualitative findings indicated a close interconnection between the various dimensions of stress. A systematic overview of the identified stressors, structured according to the components of psychological stress as defined in DIN EN ISO 10075-1:2018-01 [34], is provided in Table 2.

**Table 2:** Stressors clustered according to DIN EN ISO 10075-1:2018-01 (own illustration based on [34])

Category (according to DIN EN ISO 10075-1)	Stressors identified in studies	References	Number of studies	Interpretation/Key patterns
Work task (content-related, quantitative, qualitative)	Psychological			
	High workload	[10, 14, 16, 19, 67, 71, 72, 75, 91, 98, 107, 129]	12	Workload and emotional demands represent the most consistent and cross-setting stressors. Increasing ICT use introduces new cognitive demands.
	Non-nursing tasks	[10, 81, 129]	3	
	Emotional demands (suffering, death)	[16, 67, 74, 75, 91, 102, 107, 129]	8	
	Use of digital information and communication technologies (ICT)	[65]	1	
	Physical			
	Physically demanding work (e.g. lifting, carrying, repositioning patients)	[10, 71, 75, 86, 129]	5	Physically demanding activities remain a central burden linked to musculoskeletal disorders; ergonomic improvements are crucial.
	Work in forced postures	[71]	1	
Work organization (temporal, procedural, regulatory aspects)	Time pressure/lack of time	[10, 14, 16, 17, 19, 71, 74, 82, 90, 91, 115]	11	Organizational stressors are most frequently cited, highlighting systemic workload compression and insufficient staffing as structural drivers of strain.
	Overtime	[10, 14, 16, 17, 61–63, 86, 91, 129]	10	
	Shift work/weekend work	[10, 68, 130]	3	
	Staff shortages	[10, 14, 86, 104, 106, 107, 129]	7	
	Patient endangerment due to inadequate staffing	[10, 14]	2	
	Lack of breaks/recovery times	[16, 19, 86, 91, 129]	5	
	Disruptions/Interruptions	[71]	1	
	Inadequate remuneration	[10, 17, 72, 105, 129]	5	
	Lack of development opportunities	[17, 80]	2	
	Lack of compatibility of family and career	[17, 72, 99, 102, 119]	5	
	Unclear decision-making procedures	[14]	1	
	Lack of a say	[86, 129]	2	
	Pandemic-specific stress factors			
	Hygiene management/lack of protective equipment	[67, 105, 136]	3	Pandemic-related stressors reflect acute organizational deficiencies that heightened uncertainty and psychological exhaustion
Work environment (physical, ergonomic)	Heat exposure	[30]	1	Environmental stressors are context-specific, mainly relevant for outpatient and home-care settings.
	Adverse weather conditions	[20]	1	
	Working with microbiological substances	[71]	1	
Social relationships (leadership, team, patients)	Communication problems, conflicts (with physicians, within the team)	[14, 86, 102]	3	Interpersonal and patient-related challenges amplify emotional strain, especially where team cohesion or leadership support is weak.
	Challenging patients/relatives	[16, 75, 87, 91, 129]	5	
	Sexual harassment	[121, 122]	2	
Social conditions	Moral dilemmas	[14, 67, 84, 102, 117]	5	Moral and societal stressors reveal deeper structural and ethical challenges; lack of recognition and value is a pervasive burden.
	Low societal appreciation	[10, 72, 81, 82, 86, 119, 126, 129, 136]	9	
	Pandemic-specific stress factors			
	Moral conflicts (fear of infection, concern for family)	[105, 110, 114, 136, 137]	5	

Organizational stressors were the most consistently identified burdens. These included high workload and time pressure [10, 14, 16, 17, 19, 67, 71, 74, 82, 90, 91, 115], overtime and insufficient recovery opportunities [10, 14, 16, 17, 61–63, 86, 91, 129], and persistent staff shortages, which intensified work compression and, in critical cases, posed risks to patient safety [10, 14, 86, 104, 106, 107, 129]. These factors were frequently linked to emotional exhaustion and increased burnout risk [10, 14, 16, 19, 71, 72, 76, 86, 102].

Physical demands, particularly lifting, carrying and repositioning patients, were also prominent sources of strain and strongly associated with musculoskeletal complaints [8, 10, 71, 72, 75, 85, 86, 129]. Additional physical burdens included awkward postures and exposure to microbiological hazards [71].

Emotional and ethical stressors played a central role as well. Nurses frequently encountered distressing patient situations, suffering, and challenging interactions with patients and families [16, 67, 74, 75, 87, 91, 102, 107, 129]. Moral conflicts, role ambiguity, and insufficient participation in decision-making processes further contributed to psychological strain [14, 105, 110, 114, 136, 137].

Interpersonal and contextual stressors included workflow disruptions [71], communication problems and team conflicts [14, 86, 102], inadequate compensation [10, 17, 72, 105, 129], limited career development [17, 80], and poor work–family compatibility [17, 72, 99, 102, 119]. Irregular working hours, shift work, and weekend duties negatively affected mental health and work–life balance [9, 68, 79, 129]. A lack of organizational and societal recognition was frequently described as demotivating and burdensome [10, 72, 81, 82, 86, 119, 126, 129, 136]. Additional stressors included sexual harassment [121, 122], environmental burdens such as high ambient temperatures [30], and increasing digital demands associated with information and communication technologies, which required new competencies and could generate stress when support was lacking [65]. Collectively, these stress factors diminished motivation and, over time, weakened attachment to the workplace [10, 79, 84, 119, 129].

Nursing professionals with a migration background faced additional challenges during their integration into the German healthcare system. These included cultural and institutional discrepancies, communication barriers, role uncertainty, and perceived devaluation of competencies, which contributed to increased strain at multiple levels [81, 82].

Setting-specific differences in stress experiences were also evident. Outpatient care workers reported not only organizational uncertainties but also increased time pressure and difficulties in receiving collegial support in mobile work environments [20, 115]. Weather conditions and external regulations also represented specific stressors [20, 123]. In oncology, nurses experienced greater moral distress, especially due to a lack of involvement in treatment decisions and the emotional burden of caring for patients in palliative situations [117]. In intensive care, care omissions were reported less frequently, likely reflecting more favorable working conditions and stronger resource availability [131].

The COVID-19 pandemic substantially intensified stress levels across all settings. Studies consistently reported increased psychological, organizational, and ethical burdens, including fears of infection, lack of personal protective equipment, increased workload, emotional strain in caring for severely ill or dying patients, and the challenge of patient isolation [15, 67, 103, 105, 110, 113, 114, 136, 137]. Pandemic-related pressures amplified pre-existing structural deficits, particularly staffing shortages and workload compression, especially in inpatient care [107]. In addition, several studies documented increases in presenteeism and perceived health loss, further underscoring the wide-ranging effects on both physical and psychological wellbeing [15, 67, 103, 105, 110]. Limited recovery opportunities, inadequate leadership, and ambiguous societal recognition further increased emotional exhaustion and irritation and contributed to a heightened intention to leave the profession [67, 114]. At the same time, some studies documented protective resources such as strengthened team cohesion, shared meaning, and a sense of solidarity, which supported resilience during the pandemic [66, 67].

#### Resources and interventions for health promotion

Several studies identified personal, social and organizational resources that help stabilize psychological wellbeing and support work ability among nursing staff. Team interaction was described as one of the most important protective resources [76, 84, 130, 132]. Recognition from supervisors was perceived as relieving, whereas financial incentives played only a minor role [76, 90]. Personal contact with relatives after the death of a patient was considered helpful for coping by 44% of nurses [76]. Structural factors such as short communication pathways and effective internal communication were likewise associated with reduced work-related stress [87]. Supervisor support and greater professional autonomy were also identified as important buffers [90]. To manage moral stressors, strategies such as team meetings and collegial exchange were used, with collegial exchange rated as the most relevant, though only moderately effective, approach [84].

In addition to these resources, several studies examined health promotion interventions. A self-care training program reduced job stress and emotional exhaustion and improved emotional regulation [92]. A digital intervention grounded in positive psychology supported resilience and stress management [95]. In a randomized trial, digital cognitive behavioral therapy for insomnia improved sleep quality and mental health among shift workers [125]. Measures addressing sexual harassment indicated that a combination of policies, reporting systems and culture-oriented leadership can be effective [93]. Other interventions, such as the DEMIAN program in dementia care, reduced time pressure and increased job satisfaction [94]. The empCARE program demonstrated moderate long-term reductions in psychological strain and burnout [96], with perceived effectiveness strongly influenced by individual attitudes. A process evaluation of workplace health promotion emphasized the importance of contextual sensitivity and effective communication [139].

Low-threshold measures, such as short mindfulness exercises during breaks or after shifts, showed positive effects on recovery and mental detachment – although their practical feasibility in everyday nursing work remained limited [127]. In terms of physical health, the effects of previous interventions are less clear: A combined program of psychosocial coaching and physiotherapy showed no consistent long-term effects, although positive effects on mobility were documented [97].

A systematic literature review [69] highlighted the lack of methodologically sound studies on violence prevention and health promotion – particularly in the outpatient sector. Additionally, information on the practical implementation and acceptance of interventions is often lacking, limiting generalizability. Overall, however, the findings indicated that the success of health-promoting measures strongly depended on structural integration, target group suitability, and communication conditions.

## Discussion

Nursing staff play a critical role in healthcare delivery, making it essential to understand their health status, health behaviors and perceived work-related stress. This scoping review synthesised the available evidence to provide an overview of these dimensions among nurses in Germany. The review also aimed to identify research gaps and derive implications for future studies and health promotion strategies. To our knowledge, this is the first comprehensive synthesis of literature addressing the health status, health behavior and work-related stress of the nursing workforce in Germany.

### Summary of key findings

In total, 66 studies on health status, 75 studies on work-related stress, and three studies on health behavior were analyzed. Quantitative approaches have focused primarily on prevalences and associations, whereas qualitative and mixed-methods studies have provided deeper insights into subjective experiences and contextual mechanisms. Regionally, research activity was concentrated in western and southern Germany, indicating an uneven distribution of evidence.

The findings present a complex and, in part, alarming picture of health impairments and work-related stressors in the nursing profession. In particular, psychological complaints such as symptoms of exhaustion, burnout, depressive moods, and anxiety disorders, as well as physical ailments - especially in the musculoskeletal system and sleep disturbances - indicate a considerable occupational health risk. Additionally, the reviewed studies consistently reported a high prevalence of work-related stressors, including time pressure, staff shortages, physically demanding tasks, and emotional and moral burdens, which are closely associated with health issues and reduced job satisfaction. Tendencies toward setting-specific differences became apparent: Studies focusing on inpatient and intensive care more frequently described psychological and physical strain, whereas research in outpatient care primarily emphasized organizational uncertainty and structural challenges. This interplay between overload and health impairment is also reflected in occupational indicators such as above-average sickness absence, early retirement, and disability pensions.

The evidence also demonstrates strong interrelations between physical health, health behaviors and organizational working conditions. Musculoskeletal problems, fatigue and sleep disturbances were associated with shift work, long working hours and physically demanding tasks that limit recovery. These working conditions also shape behaviors such as physical activity, nutrition and substance use, underscoring that health-promoting behavior cannot be addressed solely at the individual level but requires supportive structural conditions.

Collectively, these factors influence job satisfaction and retention, with chronic overload, mental exhaustion and insufficient recovery resources being associated with stronger intentions to leave the profession. The findings are consistent with international research from more than 30 countries (including, for example, the US, Belgium, China, and Canada) [141, 142], which likewise indicates high morbidity and substantial work-related stress among nurses, with far-reaching consequences for individual health, professional performance and the long-term stability of nursing care.

### Research gaps and recommendations

Nonetheless, despite the broad evidence base, significant blind spots remain. While some studies have examined differences between care settings such as outpatient and inpatient nursing [e.g. 15, 115, 121, 122], they usually do not differentiate between various nursing professions. Conversely, other studies focus on specific settings and analyze profession-related stress profiles [e.g. 14, 74–76, 79, 83, 85], but do not provide comparative data across different care sectors. Consequently, differences in work-related stress and health status among outpatient, inpatient, and specialized nursing care settings remain insufficiently understood. There is a lack of systematic, comparative analyses across care contexts that are based on standardized assessment tools and are capable of adequately capturing underrepresented dimensions of occupational burden, such as technostress [65] and exposure to workplace violence [68].

With respect to health status, current evidence clearly focuses on mental health aspects such as burnout, emotional exhaustion, depression, and symptoms of anxiety. In contrast, the body of research on physical health is considerably less developed. While there are indications of an increased prevalence of musculoskeletal disorders, cardiovascular complaints, and sleep disturbances, systematic and comprehensive data on common chronic illnesses, such as hypertension, elevated blood lipid levels, type 2 diabetes, or obesity, are lacking, particularly with respect to 12-month prevalence rates, as documented, for example, in the GEDA monitoring of the general population by the Robert Koch Institute [49, 143, 144].

Many included studies relied on cross-sectional designs, which capture only single time points and therefore do not allow conclusions about temporal dynamics, causal pathways, or directionality of associations [145]. In addition, much of the evidence is based on self-reported online surveys, which are susceptible to social desirability bias, misreporting, survey fatigue and differential participation. While survey fatigue may reduce both response quality and willingness to participate, self-selection carries the risk of overrepresenting particularly burdened or highly motivated nurses. The healthy worker effect, by contrast, may lead to a systematic underrepresentation of individuals with poorer health or those who have already left the profession [146]. Further biases, such as selection bias or billing-related artefacts in secondary data analyses, may compound these limitations [147]. The evidence base also shows pronounced regional concentration, with a predominance of studies from western and southern Germany and limited data from eastern federal states and rural areas, as well as a focus on specific care sectors such as intensive, palliative, or long-term care, which restricts transferability [148]. Taken together, these methodological, regional, and sectoral imbalances substantially limit the generalizability of the findings to the wider nursing workforce in Germany and reduce their applicability to national-level decision-making and planning processes. Future research could mitigate these limitations by employing more diverse sampling strategies, longitudinal designs, or randomized controlled experimental studies. Findings on the health impact of the COVID-19 pandemic additionally underscore the need to address long-term trends through longitudinal research designs [67].

Health behavior among nursing staff has also been examined to a limited extent, despite the critical role of personal-level resources in coping with work-related stress [34]. These resources are essential for preventing excessive strain, avoiding work-related illness, and promoting health [34]. It is largely unclear to what extent nurses engage in health-promoting behaviors, which barriers they encounter, and how organisational working conditions shape these behaviors. The limited available evidence points to problematic patterns such as increased tobacco use, physical inactivity, alcohol consumption as a coping mechanism, and low uptake of workplace health promotion programs [71, 104, 130], but does not allow conclusions about systematic relationships or behavioral trajectories. This underrepresentation reflects a structural gap in the evidence base. From a public health perspective, the relevance of this gap becomes particularly apparent, as without systematic data on health behavior, important population level developments, such as prevention potential and the distribution of health related risks within this occupational group, cannot be adequately captured. Nursing research has predominantly focused on occupational stressors, whereas behavioral determinants of health have received comparatively little conceptual or methodological attention. One contributing factor may be that health promotion and worker protection are not consistently embedded in nursing practice, limiting both the visibility of these topics and their integration into research agendas. A structured monitoring system, which would be required for the development of effective and context-sensitive prevention strategies, does not yet exist, thereby complicating evidence-informed decision-making in occupational health.

Although preventive health measures are gaining increasing relevance in light of the high occupational burden in the nursing sector, consistent with previous research at both the national and international levels [69, 149], methodologically sound intervention studies specifically targeting the nursing workforce are lacking. The available measures to date have focused predominantly on promoting mental health. Some interventions, such as those addressing self-care, mindfulness, or digitally delivered cognitive behavioral therapy, have shown positive effects on psychological outcomes such as perceived stress or burnout [92, 95, 125]. However, there is still insufficient evidence regarding their long-term effectiveness and sustainable structural implementation. Notably, there is also a considerable lack of data on physical health promotion, despite the high prevalence of musculoskeletal complaints among nursing staff. Interventions targeting physical conditions such as back pain or hypertension have rarely been evaluated or have demonstrated only limited effectiveness [97]. Furthermore, consistent with previous findings [69], no intervention study has addressed the frequently reported experiences of verbal and physical violence or sexual harassment among nurses in the context of health promotion.

### Challenges in conducting research

Conducting research with nursing staff is associated with specific challenges that arise from the structural and organizational conditions of the profession and contribute substantially to the fragmented state of the evidence base. High workloads, unpredictable schedules and limited temporal flexibility substantially reduce opportunities for participation in research activities [150]. Shift work and irregular working hours complicate the planning and coordination of data collection, and participation often competes with recovery time. Access to staff is further constrained by organizational gatekeeping, varying institutional priorities and limited integration of research and occupational health structures [151]. These conditions make it difficult to recruit diverse samples, to implement longitudinal designs and to systematically engage nurses across different care settings.

### Strengths and limitations

This scoping review provides a comprehensive synthesis of existing sources of evidence and offers a broad overview of the health status, health behavior, and perceived stress of nursing staff in Germany. By incorporating a variety of study designs and data sources, such as quantitative, qualitative, and mixed methods studies, and analyses of secondary data, a wide range of perspectives could be considered that may have been overlooked by other methods of evidence synthesis.

At the same time, several limitations should be considered. Despite a systematic approach, the review cannot ensure complete coverage of all relevant studies. The search was limited to three scientific databases. Consequently, potentially relevant studies in other databases may have been missing. We deem the risk of missing relevant studies based on language restrictions (German, English) as unlikely given that the population of interest was based in Germany. Although the search algorithm included a wide range of terms to accurately reflect the concepts, other relevant terms may still exist. Furthermore, the concept of health behavior in this review was primarily defined in alignment with the SNAP framework, which focuses on smoking, nutrition, alcohol consumption, and physical activity, and therefore does not take other health-related behaviors into account. This conceptual focus may partly explain why studies on health behavior are overall less represented than those addressing physical or mental health. The search for gray literature was also constrained. While the preprint servers MedRxiv and OpenGrey were included, OpenGrey has since been discontinued, which may have limited access to certain unpublished or institutional materials. Furthermore, no expert consultation was undertaken to identify additional potentially relevant sources.

As scoping reviews do not involve a formal assessment of the methodological quality of included studies, the extent to which individual findings may be biased remains uncertain. Therefore, the results of this review should be interpreted as a broad mapping of the available evidence and as a foundation for more in-depth future research.

## Conclusions

### Overall conclusion

This review underscores the need for a stronger empirical foundation on the health behavior of nursing staff as well as for more comprehensive and differentiated data on their physical and mental health. Therefore, the establishment of a national, nursing-specific health monitoring system should be considered a priority at the national level. Such evidence is essential for designing targeted and effective measures in both practice and policy. Moreover, health-related strain, subjective perceptions of stress, individual health behavior, and structural conditions are clearly interrelated in a complex dynamic characterized by self-reinforcing feedback loops. This dynamic poses a significant risk to the long-term stability of nursing care provision.

At the same time, the results highlight a discrepancy between the internationally established occupational health standards formulated by organizations such as the ILO, EU-OSHA, and ICOH and the daily realities of nursing practices in Germany.

Addressing this development requires a dual approach: on the one hand, global policy recommendations must be translated into concrete organizational reforms; on the other hand, the profession’s own perspectives and proposals must be systematically integrated to ensure that measures are context-sensitive, practice-oriented, and sustainable. Integrated strategies are needed that go beyond individual-level interventions and include structural reforms such as improved staffing levels, health-promoting working time models, and systematic, profession-specific health monitoring. Only by taking such comprehensive action can the downward spiral of overload, illness, and staff shortages be sustainably interrupted.

Although this review focuses on the German context, the identified themes and challenges can be situated within the broader European discourse on occupational health and nursing policy. The findings thus provide starting points for comparative analyses and for policy strategies at the European level aimed at improving working conditions and promoting the health of nursing professionals.

### Implications for research

The present findings point to a clear need for future research. Methodologically sound studies with representative samples are needed to assess both the physical and mental health as well as the health behaviors of nursing staff comprehensively. Longitudinal studies, in particular, are essential for understanding the dynamics of experienced strain, mapping temporal developments, and reconstructing causal relationships. Ideally, a continuous, nursing-specific health monitoring system should be implemented, modeled after existing population-representative studies, to support political decision-making with data-based evidence.

Despite the organizational challenges associated with conducting research in this workforce, future research should systematically examine how setting-specific working conditions and sociodemographic factors such as age, gender, qualification level and migration background shape nurses’ health and health behaviors. The development and evaluation of evidence-based, practical intervention programs remain key recommendations that have thus far been addressed only sporadically.

### Implications for practice

This review highlights several practical implications for nursing in line with leading international organizations such as EU-OSHA, the ILO and ICOH. Individual-level approaches, such as self-care strategies, stress management training, or low-threshold workplace health promotion activities, can support nurses in coping with daily demands. However, their effectiveness remains limited if they are not accompanied by appropriate structural conditions. Accordingly, sustainable improvements in nurses’ health therefore require, above all, changes in the work environment. These include reducing workloads; expanding staffing levels; improving scheduling practices; and strengthening participation, appreciation, and social support in daily work life.

Leadership plays a central role in shaping health-promoting conditions and fostering a culture of open communication. Workplace health promotion that is flexible, easily accessible, and tailored to the realities of nursing can make an important contribution to prevention - provided that it is firmly embedded within organizational structures.

Finally, broader societal and political recognition of the nursing profession is essential. This should be reflected in adequate pay, reliable career prospects and greater professional autonomy. Only under such conditions can health-related burdens be effectively reduced and the long-term attractiveness of the profession can be maintained.

## Supplementary Information

Below is the link to the electronic supplementary material.

Supplementary Material 1: Additional file 1: File format: .docx. Title of data: Search strategy. Description of data: Detailed description of the search strategy used in the review, including the databases searched, keywords, and search strings

Supplementary Material 2: Additional file 2: File format: .docx. Title of data: List of websites examined as part of the web search. Description of data: A comprehensive list of websites that were included in the web search component of the study

Supplementary Material 3: Additional file 3: File format: .docx. Title of data: Sources ineligible following full-text review. Description of data: List of studies excluded after full-text review, along with reasons for exclusion and the number of records excluded (n = 80)

Supplementary Material 4: Additional file 4: File format: .docx. Title of data: Data extraction instrument based on Peters et al. Description of data: The data extraction form developed for this review, adapted from the framework by Peters et al

Supplementary Material 5: Additional file 5: File format: .docx. Title of data: Detailed study description and results. Description of data: A table presenting the detailed characteristics and findings of the included studies

## Data Availability

The datasets generated during and/or analyzed during the current study are available from the corresponding author upon reasonable request.

## Declarations

### Writing assistance use of AI assistance

During the preparation of this work, the author made use of ChatGPT (ChatGPT-4o) to assist with grammar and spelling checks, as well as to rephrase sentences for improved clarity. Additionally, the AI-based language quality checker Curie (developed by AJE – American Journal Experts) was used to further refine the manuscript’s linguistic quality. All content generated with the help of these tools was carefully reviewed and revised by the authors. The authors assume full responsibility for the final content of this publication. No data analysis or interpretation was conducted using artificial intelligence.

## Abbreviations

CBI: Copenhagen Burnout Inventory
CBT-I: Cognitive Behavioral Therapy for Insomnia
COPSOQ: Copenhagen Psychosocial Questionnaire
DASS: Depression Anxiety and Stress Scale
GEDA: German Health Update
ICTs: Information and Communication Technologies
JBI: Joanna Briggs Institute
MBI: Maslach Burnout Inventory
PHQ: Patient Health Questionnaire
PRESS: Peer Review of Electronic Search Strategies
PRISMA-ScR: Preferred Reporting Items for Systematic Reviews and Meta-Analyses extension for Scoping Reviews
RN4CAST: Nurse forecasting in Europe
SNAP: Smoking, Nutrition, Alcohol consumption, Physical activity
