# Supplementary material for: Health status, health behavior and perceived stress of nursing staff in Germany: a scoping review
Source: BMC Nurs. 2026 Jan 9;25:97. doi: 10.1186/s12912-025-04282-4 (PMC12849386; doi:10.1186/s12912-025-04282-4)
Supplement: Supplementary file 1 — Supplementary Material 1: Additional file 1: File format: .docx. Title of data: Search strategy. Description of data: Detailed description of the search strategy used in the review, including the databases searched, keywords, and search strings [file 12912_2025_4282_MOESM1_ESM.docx]

**Additional file 1: Search strategy**

**Applied search strategy for CINAHL (own illustration)**

Search conducted: March 5, 2025

| **Inclusion criteria** | **Definition** | **Search term** | **Results retrieved** |
| --- | --- | --- | --- |
| **Participants** | | | |
|  | Nursing staff | ‘nurses or nursing staff or nurse or nurs* carers or outpatient care’ [#1]* | 133,059 |
| **Concept** | | | |
|  | *Health*  (Indicators: e.g. 12-month prevalence of certain diseases and symptoms, subjective complaints) | #1 AND (‘health status’ OR ‘subjective health status’ OR ‘musculoskeletal complaint’ OR ‘12 month prevalence’ OR ‘depression’ OR ‘anxiety disorder’ OR ‘memory problems’ OR ‘Burnout’ OR ‘cardiovascular risk’ [#2])* | 12,154 |
|  | *Health behavior*  Indicators: e.g. dietary patterns, exercise patterns, lifestyle) | #1 AND (‘body weight’ OR ‘obesity’ OR ‘overweight’ OR ‘lifestyle’ OR ‘eating behavior*’ OR ‘nutritional habit*’ OR ‘physical activity*’ OR ‘tobacco use’ OR ‘smoking’ OR ‘alcohol*’ OR ‘alcohol use’ OR ‘alcohol consumption’ [#3])* | 6,894 |
|  | *Work-related stress*  *(Indicators: e.g. job satisfaction, stress, self-efficacy)* | #1 AND (‘job satisfaction’ OR ‘wellbeing’ OR ‘welfare’ OR ‘strain’ OR ‘working conditions’ OR ‘work related behavior’ [#4])* | 10,956 |
| **Context** | | | |
|  | Germany | #1 AND ‘germany OR german’ [#5]* | 1,324 |
| Search string 1 | | #1 AND #2 AND #5 | 128 |
| Search string 2 | | #1 AND #3 AND #5 | 55 |
| Search string 3 | | #1 AND #4 AND #5 | 134 |
| *Filter: Abstract available, in the last 10 years | | | |

**Applied search strategy for MEDLINE (PubMed). (own illustration)**

Search conducted: March 5, 2025

| **Inclusion criteria** | **Definition** | **Search term** | **Results retrieved** |
| --- | --- | --- | --- |
| **Participants** | | | |
|  | Nursing staff | ‘nurses or nursing staff or nurse or nurs* care or outpatient care’ [#1]* | 69,860 |
| **Concept** | | | |
|  | *Health*  (Indicators: e.g. 12-month prevalence of certain diseases and symptoms, subjective complaints) | #1 AND (‘health status’ OR ‘subjective health status’ OR ‘musculoskeletal complaint’ OR ‘12 month prevalence’ OR ‘depression’ OR ‘anxiety disorder’ OR ‘memory problems’ OR ‘Burnout’ OR ‘cardiovascular risk’ [#2])* | 20,238 |
|  | *Health behavior*  Indicators: e.g. dietary patterns, exercise patterns, lifestyle) | #1 AND (‘body weight’ OR ‘obesity’ OR ‘overweight’ OR ‘lifestyle’ OR ‘eating behavior*’ OR ‘nutritional habit*’ OR ‘physical activity*’ OR ‘tobacco use’ OR ‘smoking’ OR ‘alcohol*’ OR ‘alcohol use’ OR ‘alcohol consumption’ [#3])* | 7,712 |
|  | *Work-related stress*  *(Indicators: e.g. job satisfaction, stress, self-efficacy)* | #1 AND (‘job satisfaction’ OR ‘wellbeing’ OR ‘welfare’ OR ‘strain’ OR ‘working conditions’ OR ‘work related behavior’ [#4])* | 7,208 |
| **Context** | | | |
|  | Germany | #1 AND ‘germany OR german’ [#5]* | 3,755 |
| Search string 1 | | #1 AND #2 AND #5 | 1,308 |
| Search string 2 | | #1 AND #3 AND #5 | 442 |
| Search string 3 | | #1 AND #4 AND #5 | 425 |
| *Filters: in the last 10 years, Abstract, Full text, Case Reports, Classical Article, Clinical Study, Clinical Trial, Clinical Trial, Phase I, Clinical Trial, Phase II, Clinical Trial, Phase III, Clinical Trial, Phase IV, Clinical Trial Protocol, Comparative Study, Meta-Analysis, Observational Study, Randomized Controlled Trial, Randomized Controlled Trial, Veterinary, Review, Systematic Review, Technical Report, Clinical Trial, Veterinary, Observational Study, Veterinary, German, English, Humans, MEDLINE | | | |

**Applied search strategy for CareLit. (own illustration)**

Search conducted: March 5, 2025

| **Inclusion criteria** | **Definition** | **Search term** | **Results retrieved** |
| --- | --- | --- | --- |
| **Participants** | | | |
|  | Nursing staff | ‘nurses or nursing staff or nurse or nurs* care or outpatient care’ [#1]* | 6,205 |
| **Concept** | | | |
|  | *Health*  (Indicators: e.g. 12-month prevalence of certain diseases and symptoms, subjective complaints) | #1 AND (‘health status’ OR ‘subjective health status’ OR ‘musculoskeletal complaint’ OR ‘12 month prevalence’ OR ‘depression’ OR ‘anxiety disorder’ OR ‘memory problems’ OR ‘Burnout’ OR ‘cardiovascular risk’ [#2])* | 2,780 |
|  | *Health behavior*  Indicators: e.g. dietary patterns, exercise patterns, lifestyle) | #1 AND (‘body weight’ OR ‘obesity’ OR ‘overweight’ OR ‘lifestyle’ OR ‘eating behavior*’ OR ‘nutritional habit*’ OR ‘physical activity*’ OR ‘tobacco use’ OR ‘smoking’ OR ‘alcohol*’ OR ‘alcohol use’ OR ‘alcohol consumption’ [#3])* | 198 |
|  | *Work-related stress*  *(Indicators: e.g. job satisfaction, stress, self-efficacy)* | #1 AND (‘job satisfaction’ OR ‘wellbeing’ OR ‘welfare’ OR ‘strain’ OR ‘working conditions’ OR ‘work related behavior’ [#4])* | 402 |
| **Context** | | | |
|  | Germany | #1 AND ‘germany OR german’ [#5]* | 712 |
| Search string 1 | | #1 AND #2 AND #5 | 179 |
| Search string 2 | | #1 AND #3 AND #5 | 3 |
| Search string 3 | | #1 AND #4 AND #5 | 17 |
| *Filter: from 2015 to 2025 | | | |

**Applied search strategy for medRxiv. (own illustration)**

Search conducted: March 5, 2025

| **Inclusion criteria** | **Definition** | **Search term** | **Results retrieved** |
| --- | --- | --- | --- |
| **Participants** | | | |
|  | Nursing staff | ‘nurses or nursing staff or nurse or nurs* care or outpatient care’ [#1]* | 59,332 |
| **Context** | | | |
|  | Germany | #1 AND ‘germany OR german’ [#2]* | 3,959 |
| *Filter: posted between 05. March 2015 and 05. March 2025 | | | |

**Applied search strategy for OpenGrey (DANS Data Station). (own illustration)**

Search conducted: March 5, 2025

| **DANS Data Station Social Sciences and Humanities** | | | |
| --- | --- | --- | --- |
| **Inclusion criteria** | **Definition** | **Search term** | **Results retrieved** |
| Participants | | | |
|  | Nursing staff | ‘nurses or nursing staff or nurse or nurs* care or outpatient care’ [#1] | 2,650 |
| Context | | | |
|  | Germany | #1 AND ‘germany OR german’ [#2] | 264 |
|  |  |  |  |
| **DANS Data Station Life Sciences** | | | |
| **Inclusion criteria** | **Definition** | **Search term** | **Results retrieved** |
| **Participants** |  |  |  |
|  | Nursing staff | ‘nurses or nursing staff or nurse or nurs* care or outpatient care’ [#1]* | 814 |
| **Context** |  |  |  |
|  | Germany | #1 AND ‘germany OR german’ [#2] | 20 |
