# Supplementary material for: Health status, health behavior and perceived stress of nursing staff in Germany: a scoping review
Source: BMC Nurs. 2026 Jan 9;25:97. doi: 10.1186/s12912-025-04282-4 (PMC12849386; doi:10.1186/s12912-025-04282-4)
Supplement: Supplementary file 2 — Supplementary Material 2: Additional file 2: File format: .docx. Title of data: List of websites examined as part of the web search. Description of data: A comprehensive list of websites that were included in the web search component of the study [file 12912_2025_4282_MOESM2_ESM.docx]

**Additional file 2: List of websites examined as part of the web search**

Search conducted: March 5, 2025

|  | **Organization/Institution** | **Website Address** | **Results**  **Retrieved*** |
| --- | --- | --- | --- |
| 1 | Barmer Institut für Gesundheitssystemforschung (BIFG) | https://www.bifg.de/publikationen/reporte/ | 1 |
| 2 | Bertelsmann-Stiftung | https://www.bertelsmann-stiftung.de/de/startseite | 0 |
| 3 | Berufsgesundheits-Index (BeGX) | https://www.bgw-online.de/bgw-online-de/service/medien-arbeitshilfen/medien-center/fachwissen/begx-2024-114162 | 3 |
| 4 | Dachverband der Betriebskrankenkassen  (BKK Dachverband) | https://www.bkk-dachverband.de/publikationen | 1 |
| 5 | Bundesanstalt für Arbeitsschutz und Arbeitsmedizin (BAuA) | https://www.baua.de/DE/Angebote/Publikationen | 1 |
| 6 | Bundesverband Ambulante Dienste und Stationäre Einrichtungen e.V. (BAD) | https://www.bad-ev.de/ | 0 |
| 7 | DAK Gesundheit | https://www.dak.de/dak/unternehmen/reporte-forschung_28136 | 3 |
| 8 | Deutsche Gesellschaft für Pflegewissenschaft | https://dg-pflegewissenschaft.de/veroeffentlichungen/publikationen/ | 0 |
| 9 | Deutsches Institut für angewandte Pflegeforschung e.V. (DIP) | https://www.dip.de/aktuelles/ | 2 |
| 10 | Deutscher Pflegerat e.V. (DPR) | https://deutscher-pflegerat.de/ | 0 |
| 11 | Kaufmännische Krankenkasse (KKH) | https://www.kkh.de/presse | 0 |
| 12 | Techniker Krankenkasse (TKK) | https://www.tk.de/firmenkunden/service/gesund-arbeiten/gesundheitsberichterstattung-2031464 | 1 |
| 13 | Verband der Ersatzkassen e.V. (VDEK) | https://www.vdek.com/ | 0 |
| 14 | Zentrum für Qualität in der Pflege (ZQP) | https://www.zqp.de/angebote/studien/ | 1 |
| *posted between 5. March 2015 and 5. March 2025 | | | |
