# Supplementary material for: Health status, health behavior and perceived stress of nursing staff in Germany: a scoping review
Source: BMC Nurs. 2026 Jan 9;25:97. doi: 10.1186/s12912-025-04282-4 (PMC12849386; doi:10.1186/s12912-025-04282-4)
Supplement: Supplementary file 3 — Supplementary Material 3: Additional file 3: File format: .docx. Title of data: Sources ineligible following full-text review. Description of data: List of studies excluded after full-text review, along with reasons for exclusion and the number of records excluded (n = 80) [file 12912_2025_4282_MOESM3_ESM.docx]

**Additional file 3: Sources ineligible following full-text review (n=80)**

| **Reason for exclusion:** | **References** | **Number of studies (n)** |
| --- | --- | --- |
| Ineligible context – Not related to Germany | [1-22] | 22 |
| Data collection older than 10 years | [23-39] | 17 |
| Ineligible population – non-professional care in focus | [40-60] | 21 |
| Ineligible concept – no concept-related results | [61-64] | 4 |
| Ineligible Publication – protocol only | [65] | 1 |
| No access to the full text | [66-70] | 5 |
| Duplicate evidence – Standard duplicates as well as reviews in which concept- or context-relevant primary studies have already been included separately, or vice versa | [71-80] | 10 |

**References:**

1. Ackerhans S, Huynh T, Kaiser C, Schultz C. Exploring the role of professional identity in the implementation of clinical decision support systems-a narrative review. Implement Sci. 2024;19(1):11. <https://doi.org/10.1186/s13012-024-01339-x>.

2. Aust B, Leduc C, Cresswell-Smith J, O'Brien C, Rugulies R, Leduc M, et al. The effects of different types of organisational workplace mental health interventions on mental health and wellbeing in healthcare workers: a systematic review. Int Arch Occup Environ Health. 2024;97(5):485-522. <https://doi.org/10.1007/s00420-024-02065-z>.

3. Bohlken J, Schömig F, Lemke MR, Pumberger M, Riedel-Heller SG. COVID-19 Pandemic: Stress Experience of Healthcare Workers - A Short Current Review. Psychiatr Prax. 2020;47(4):190-7. <https://doi.org/10.1055/a-1159-5551>.

4. Elfering A, Grebner S, Leitner M, Hirschmüller A, Kubosch EJ, Baur H. Quantitative work demands, emotional demands, and cognitive stress symptoms in surgery nurses. Psychology, Health & Medicine. 2017;22(5):604-10. <http://dx.doi.org/10.1080/13548506.2016.1200731>.

5. Hegewald J, Berge W, Heinrich P, Staudte R, Freiberg A, Scharfe J, et al. Do Technical Aids for Patient Handling Prevent Musculoskeletal Complaints in Health Care Workers?-A Systematic Review of Intervention Studies. Int J Environ Res Public Health. 2018;15(3). <https://doi.org/10.3390/ijerph15030476>.

6. Hoedl M, Bauer S, Eglseer D. Influence of nursing staff working hours on stress levels during the COVID-19 pandemic: A cross-sectional online survey. HeilberufeScience. 2021;12(3-4):92-8. <https://doi.org/10.1007/s16024-021-00354-y>.

7. Kaihlanen AM, Gluschkoff K, Koskinen S, Salminen L, Strandell‐Laine C, Fuster Linares P, et al. Final clinical practicum shapes the transition experience and occupational commitment of newly graduated nurses in Europe—A longitudinal study. J Adv Nurs. 2021;77(12):4782-92. <https://doi.org/10.1111/jan.15060>.

8. Major A, Hlubocky FJ. Mental health of health care workers during the COVID-19 pandemic and evidence-based frameworks for mitigation: A rapid review. medRxiv. 2021:2021.01.03.21249166. <https://www.medrxiv.org/content/medrxiv/early/2021/01/04/2021.01.03.21249166.full.pdf>. Accessed 01 July 2025

9. Nieuwenhuijze M, Leahy-Warren P, Healy M, Aktaş S, Aydin R, Calleja-Agius J, et al. The impact of severe perinatal events on maternity care providers: a scoping review. BMC Health Serv Res. 2024;24(1):171. <https://doi.org/10.1186/s12913-024-10595-y>.

10. Otto AK, Gutsch C, Bischoff LL, Wollesen B. Interventions to promote physical and mental health of nurses in elderly care: A systematic review. Prev Med. 2021;148:106591. <https://doi.org/10.1016/j.ypmed.2021.106591>.

11. Richter K, Peter L, Rodenbeck A, Weess HG, Riedel-Heller SG, Hillemacher T. Shiftwork and Alcohol Consumption: A Systematic Review of the Literature. Eur Addict Res. 2021;27(1):9-15. <https://doi.org/10.1159/000507573>.

12. Romero Starke K, Friedrich S, Schubert M, Kämpf D, Girbig M, Pretzsch A, et al. Are Healthcare Workers at an Increased Risk for Obstructive Respiratory Diseases Due to Cleaning and Disinfection Agents? A Systematic Review and Meta-Analysis. Int J Environ Res Public Health. 2021;18(10). <https://doi.org/10.3390/ijerph18105159>.

13. Sanfilippo F, Noto A, Foresta G, Santonocito C, Palumbo GJ, Arcadipane A, et al. Incidence and Factors Associated with Burnout in Anesthesiology: A Systematic Review. Biomed Res Int. 2017;2017:8648925. <https://doi.org/10.1155/2017/8648925>.

14. Schlicht L, Wendsche J, Melzer M, Tschetsche L, Rösler U. Digital technologies in nursing: An umbrella review. Int J Nurs Stud. 2025;161:104950. <https://doi.org/10.1016/j.ijnurstu.2024.104950>.

15. Schmitz-Rixen T, Grundmann RT. Hospital nursing personnel staffing and patient outcome. Chirurg. 2020;91(4):329-36. <https://doi.org/10.1007/s00104-019-01076-8>.

16. Schneider A, Weigl M. Associations between psychosocial work factors and provider mental well-being in emergency departments: A systematic review. PLoS One. 2018;13(6):e0197375. <https://doi.org/10.1371/journal.pone.0197375>.

17. Stemmer R, Bassi E, Ezra S, Harvey C, Jojo N, Meyer G, et al. A systematic review: Unfinished nursing care and the impact on the nurse outcomes of job satisfaction, burnout, intention-to-leave and turnover. J Adv Nurs. 2022;78(8):2290-303. <https://doi.org/10.1111/jan.15286>.

18. Suhonen R, Stolt M, Habermann M, Hjaltadottir I, Vryonides S, Tonnessen S, et al. Ethical elements in priority setting in nursing care: A scoping review. Int J Nurs Stud. 2018;88:25-42. <https://doi.org/10.1016/j.ijnurstu.2018.08.006>.

19. Wendsche J, Ghadiri A, Bengsch A, Wegge J. Antecedents and outcomes of nurses' rest break organization: A scoping review. Int J Nurs Stud. 2017;75:65-80. <https://doi.org/10.1016/j.ijnurstu.2017.07.005>.

20. Kern S, Jerg-Bretzke L, Beschoner P. Psychotherapeutic burnout interventions-an umbrella review. Bundesgesundheitsblatt Gesundheitsforschung Gesundheitsschutz. 2024;67(11):1279-87. <https://doi.org/10.1007/s00103-024-03961-y>.

21. Kanstrup M, Singh L, Leehr EJ, Göransson KE, Pihlgren SA, Iyadurai L, et al. A guided single session intervention to reduce intrusive memories of work-related trauma: a randomised controlled trial with healthcare workers in the COVID-19 pandemic. BMC Med. 2024;22(1):403. <https://doi.org/10.1186/s12916-024-03569-8>.

22. Schmitt N, Mattern E, Cignacco E, Seliger G, König-Bachmann M, Striebich S, et al. Effects of the Covid-19 pandemic on maternity staff in 2020 - a scoping review. BMC Health Serv Res. 2021;21(1):1364. <https://doi.org/10.1186/s12913-021-07377-1>.

23. Alameddine M, Otterbach S, Rafii B, Sousa-Poza A. Work hour constraints in the German nursing workforce: A quarter of a century in review. Health Policy. 2018;122(10):1101-8. <https://research.ebsco.com/linkprocessor/plink?id=d5a8180a-2396-3c5c-bc44-f6af06f443a4>.

24. Becker A, Angerer P, Müller A. The prevention of musculoskeletal complaints: a randomized controlled trial on additional effects of a work-related psychosocial coaching intervention compared to physiotherapy alone. Int Arch Occup Environ Health. 2017;90(4):357-71. <https://doi.org/10.1007/s00420-017-1202-6>.

25. Czakert J, Schirmaier A, Blakeslee SB, Stritter W, Koch AK, Kessler C, et al. Scoping review meets expert interviews: Key issues of multimodal programs for workplace health promotion in long-term care facilities –. Health Promotion Perspectives. 2024;14(3):221-37. <https://doi.org/10.34172/hpp.42899>.

26. Frey D, Rieger S, Diehl E, Pinzon LCE. Einflussfaktoren auf chronische Rückenschmerzen bei Pflegekräften in der Altenpflege in Rheinland-Pfalz. Gesundheitswesen. 2018;80(2):172-5. <https://doi.org/10.1055/s-0043-104693>.

27. Gágyor I, Heßling A, Heim S, Frewer A, Nauck F, Himmel W. Ethical challenges in primary care: a focus group study with general practitioners, nurses and informal caregivers. Family Practice. 2019;36(2):225-30. <https://doi.org/10.1093/fampra/cmy060>.

28. Kunzweiler K, Voigt K, Kugler J, Hirsch K, Bergmann A, Riemenschneider H. Factors influencing sleep quality among nursing staff: Results of a cross sectional study. Applied Nursing Research. 2016;32:241-4. <https://doi.org/10.1016/j.apnr.2016.08.007>.

29. Landfeldt E, Edström J, Buccella F, Kirschner J, Lochmüller H. Duchenne muscular dystrophy and caregiver burden: a systematic review. Dev Med Child Neurol. 2018;60(10):987-96. <https://doi.org/10.1111/dmcn.13934>.

30. Maatouk I, Müller A, Angerer P, Schmook R, Nikendei C, Herbst K, et al. Healthy ageing at work- Efficacy of group interventions on the mental health of nurses aged 45 and older: Results of a randomised, controlled trial. PLoS One. 2018;13(1):e0191000. <https://doi.org/10.1371/journal.pone.0191000>.

31. Meraner V, Sperner-Unterweger B. Patients, physicians and nursing personnel in intensive care units: Psychological and psychotherapeutic interventions. Nervenarzt. 2016;87(3):264-8. <https://doi.org/10.1007/s00115-016-0098-9>.

32. Müller A, Heiden B, Herbig B, Poppe F, Angerer P. Improving well-being at work: A randomized controlled intervention based on selection, optimization, and compensation. J Occup Health Psychol. 2016;21(2):169-81. <https://doi.org/10.1037/a0039676>.

33. Pinkert C, Faul E, Saxer S, Burgstaller M, Kamleitner D, Mayer H. Experiences of nurses with the care of patients with dementia in acute hospitals: A secondary analysis. J Clin Nurs. 2018;27(1-2):162-72. <https://doi.org/10.1111/jocn.13864>.

34. Schneider D, Winter V, Schreyögg J. Job demands, job resources, and behavior in times of sickness: An analysis across German nursing homes. Health Care Manage Rev. 2018;43(4):338-47. <https://doi.org/10.1097/HMR.0000000000000157>.

35. Schröder C, Nienhaus A. Intervertebral Disc Disease of the Lumbar Spine in Health Personnel with Occupational Exposure to Patient Handling-A Systematic Literature Review and Meta-Analysis. Int J Environ Res Public Health. 2020;17(13). <https://doi.org/10.3390/ijerph17134832>.

36. Tebest R, Yoon Mee Honervogt F, Westermann K, Samel C, Redaèlli M, Stock S. Hygienebeauftragte in der Pflege auf den Stationen: Erste Erfahrungen zur Einführung im Universitätsklinikum Köln. Pflege. 2017;30(5):271-80. <https://doi.org/10.1024/1012-5302/a000534>.

37. Weigl M, Stab N, Herms I, Angerer P, Hacker W, Glaser J. The associations of supervisor support and work overload with burnout and depression: a cross-sectional study in two nursing settings. J Adv Nurs. 2016;72(8):1774-88. <https://doi.org/10.1111/jan.12948>.

38. Wendsche J, Hacker W, Wegge J, Rudolf M. High Job Demands and Low Job Control Increase Nurses' Professional Leaving Intentions: The Role of Care Setting and Profit Orientation. Research in Nursing & Health. 2016;39(5):353-63. <https://doi.org/10.1002/nur.21729>.

39. Schilgen B, Nienhaus A, Handtke O, Schulz H, Mösko M. Health situation of migrant and minority nurses: A systematic review. PLoS One. 2017;12(6):e0179183. <https://doi.org/10.1371/journal.pone.0179183>.

40. Baumann H, Heuel L, Bischoff LL, Wollesen B. Efficacy of Individualized Sensory-Based mHealth Interventions to Improve Distress Coping in Healthcare Professionals: A Multi-Arm Parallel-Group Randomized Controlled Trial. Sensors (Basel). 2023;23(4). <https://doi.org/10.3390/s23042322>.

41. Chemali S, Mari-Sáez A, El Bcheraoui C, Weishaar H. Health care workers' experiences during the COVID-19 pandemic: a scoping review. Hum Resour Health. 2022;20(1):27. <https://doi.org/10.1186/s12960-022-00724-1>.

42. Garthe N, Hasselhorn HM. Die Erwerbsperspektive in Berufen des Gesundheitsdienstes – Wie lange wollen, können und planen ältere Erwerbstätige (zu) arbeiten? Gesundheitswesen. 2022;85(06):514-21. <https://doi.org/10.1055/a-1915-4324>.

43. Grüne E, Popp J, Carl J, Semrau J, Pfeifer K. Examining the sustainability and effectiveness of co-created physical activity interventions in vocational education and training: a multimethod evaluation. BMC Public Health. 2022;22(1):765. <https://doi.org/10.1186/s12889-022-13133-9>.

44. Härkänen M, Pineda AL, Tella S, Mahat S, Panella M, Ratti M, et al. The impact of emotional support on healthcare workers and students coping with COVID-19, and other SARS-CoV pandemics - a mixed-methods systematic review. BMC Health Serv Res. 2023;23(1):751. <https://doi.org/10.1186/s12913-023-09744-6>.

45. Hoerold M, Gottschalk M, Debbeler CM, Heytens H, Ehrentreich S, Braun-Dullaeus RC, et al. Healthcare professionals' perceptions of impacts of the Covid-19-pandemic on outpatient care in rural areas: a qualitative study. BMC Health Serv Res. 2021;21(1):1-11. <https://doi.org/10.1186/s12913-021-07261-y>.

46. Hoffmann S, Schulze S, Löffler A, Becker J, Hufert F, Gremmels H-D, et al. Did the prevalence of depressive symptoms change during the COVID-19 pandemic? A multilevel analysis on longitudinal data from healthcare workers. International Journal of Social Psychiatry. 2024;70(1):87-98. <https://doi.org/10.1177/00207640231196737>.

47. Jerg-Bretzke L, Karremann M, Beschoner P, de Gregorio N, Schochter F, Janni W, et al. Psychosoziale Arbeitsbelastung und Gesundheit von Beschäftigten einer Universitätsfrauenklinik im Berufsgruppenvergleich. Pflegewissenschaft. 2021;23(2):91-7. <https://doi.org/10.3936/1819>.

48. Kaltwasser A, Pelz S, Nydahl P, Dubb R, Borzikowsky C. Querschnittsstudie zu Arbeitsbedingungen und Versorgungsqualität in der Versorgung von COVID-19-Patienten. Anaesthesist. 2021;70(9):753-60. <https://doi.org/10.1007/s00101-021-00919-6>.

49. Kulikova O, Hering T. Muskuloskelletale Beschwerden bei Studierenden einer Hochschule für Gesundheit und damit verbundene Absichten, das Studium zu wechseln oder abzubrechen. Pravent Gesundh. 2019;14(3):242-7. <https://doi.org/10.1007/s11553-018-0695-5>.

50. Kunzler AM, Helmreich I, König J, Chmitorz A, Wessa M, Binder H, et al. Psychological interventions to foster resilience in healthcare students. Cochrane Database Syst Rev. 2020;7(7):Cd013684. <https://doi.org/10.1002/14651858.CD013684>.

51. Küster D, Haufe E, Rethberg C, Bauer A, Seidler A, Schmitt J. Health utilities for controlled and uncontrolled chronic hand eczema in healthcare employees. Contact Dermatitis. 2018;78(1):18-27. <https://doi.org/10.1111/cod.12863>.

52. Niens C. „Aber ich bin jetzt eben so weit, dass ich mir sage, ich schaffe es so einfach nicht mehr. Pflegewissenschaft. 2019;21(11/12):413-28. <https://doi.org/10.3936/1732>.

53. Orellana-Rios CL, Radbruch L, Kern M, Regel YU, Anton A, Sinclair S, et al. Mindfulness and compassion-oriented practices at work reduce distress and enhance self-care of palliative care teams: a mixed-method evaluation of an "on the job" program. BMC Palliat Care. 2017;17(1):3. <https://doi.org/10.1186/s12904-017-0219-7>.

54. Schneider A, Wehler M, Weigl M. Effects of work conditions on provider mental well-being and quality of care: a mixed-methods intervention study in the emergency department. BMC Emerg Med. 2019;19(1):N.PAG-N.PAG. <https://doi.org/10.1186/s12873-018-0218-x>.

55. van Diepen C, Fors A, Ekman I, Hensing G. Association between person-centred care and healthcare providers' job satisfaction and work-related health: a scoping review. BMJ Open. 2020;10(12):e042658. <https://doi.org/10.1136/bmjopen-2020-042658>.

56. Weber J, Tzivian L, Müller A, Angerer P. Country‐specific differences of age stereotypes towards older hospital staff and their association with self‐efficacy, work ability and mental well‐being. J Adv Nurs. 2020;76(7):1614-26. <https://doi.org/10.1111/jan.14380>.

57. Wirth T, Peters C, Nienhaus A, Schablon A. Interventions for Workplace Violence Prevention in Emergency Departments: A Systematic Review. Int J Environ Res Public Health. 2021;18(16). https:/doi.org/10.3390/ijerph18168459.

58. Frenkel MO, Pollak KM, Schilling O, Voigt L, Fritzsching B, Wrzus C, et al. Stressors faced by healthcare professionals and coping strategies during the early stage of the COVID-19 pandemic in Germany. PLoS One. 2022;17(1):e0261502. <https://doi.org/10.1371/journal.pone.0261502>.

59. Hinzmann D, Haneveld J, Heininger SK, Spitznagel N. Is it time to rethink education and training? Learning how to perform under pressure: An observational study. Medicine (Baltimore). 2022;101(52):e32302. <https://doi.org/10.1097/MD.0000000000032302>.

60. Tisch A, Meyer SC. Risks and opportunities of digitisation in the professional fields of nursing, care and healing. Bundesgesundheitsblatt Gesundheitsforschung Gesundheitsschutz. 2020;63(6):690-7. <https://doi.org/10.1007/s00103-020-03160-5>.

61. Ambrosch A, Wahrburg K, Klawonn F. Bacterial load and pathogenic species on healthcare personnel attire: implications of alcohol hand-rub use, profession, and time of duty. J Hosp Infect. 2019;101(4):414-21. <https://doi.org/10.1016/j.jhin.2018.10.017>.

62. Berger S, Brandauer A, Freywald N, Höppchen I, Kutschar P, Strobl A, et al. Wissensstand und Informationsbedarfe von Pflegefachkräften zum Hygienemanagement in Wohngemeinschaften der außerklinischen Intensivpflege. HeilberufeScience. 2023;14(1):19-27. <https://doi.org/10.1007/s16024-022-00380-4>.

63. Bruyneel L, Lesaffre E, Meuleman B, Sermeus W. Power Distance and Physician–Nurse Collegial Relations Across 14 European Countries: National Culture is Not Merely a Nuisance Factor in International Comparative Research. J Nurs Scholarsh. 2019;51(6):708-16. <https://doi.org/10.1111/jnu.12514>.

64. Przysucha M, Flemming D, Schulte G, Hübner U. Cognitive Performance of Users Is Affected by Electronic Handovers Depending on Role, Task and Human Factors. Stud Health Technol Inform. 2017;243:117-21. <https://doi.org/10.3233/978-1-61499-808-2-117>.

65. Koch AK, Schröter M, Berschick J, Schiele JK, Bogdanski M, Steinmetz M, et al. A custom tailored, evidence-based, theory-informed intervention for healthcare professionals to prevent burnout (LAGOM): study protocol for a pragmatic randomized controlled trial. Trials. 2024;25(1):628. <https://doi.org/10.1186/s13063-024-08491-1>.

66. Bernburg M, Groneberg DA, Mache S. Mental Health Promotion Intervention for Nurses Working in German Psychiatric Hospital Departments: A Pilot Study. Issues Ment Health Nurs. 2019;40(8):706-11. <https://doi.org/10.1080/01612840.2019.1565878>.

67. Buruck G, Dörfel D, Kugler J, Brom SS. Enhancing well-being at work: The role of emotion regulation skills as personal resources. J Occup Health Psychol. 2016;21(4):480-93. <https://doi.org/10.1037/ocp0000023>.

68. Kunz M, Strasser M, Hasan A. Impact of the coronavirus disease 2019 pandemic on healthcare workers: systematic comparison between nurses and medical doctors. Curr Opin Psychiatry. 2021;34(4):413-9. <https://doi.org/10.1097/YCO.0000000000000721>.

69. Wahlster S, Hartog C. Coronavirus disease 2019 aftermath: psychological trauma in ICU healthcare workers. Curr Opin Crit Care. 2022;28(6):686-94. <https://doi.org/10.1097/MCC.0000000000000994>.

70. Schulze S, Sappl I, Uhlenbrock G, Thier A, Rapp M, Spallek J, et al. Belastungen von Pflegenden während der Coronakrise in Deutschland: Ein narratives Review. Pflegewissenschaft. 2022;5:285-94. <https://doi.org/10.3936/22c2c23c>.

71. Schwarzkopf D. Nonbeneficial care - a burden for clinicians and relatives. Med Klin Intensivmed Notfmed. 2019;114(3):222-8. <https://doi.org/10.1007/s00063-019-0531-5>.

72. Wirth LM, Ruppert N, Büscher A, Hülsken-Giesler M. Gesundheitsorientierte Führung in der Pflege–Einflussmöglichkeiten der Führungspersonen auf die Gesundheit von Pflegefachpersonen. Pflege und Gesellschaft 2022;4. <https://doi.org/10.3262/PUG2204322>.

73. Mojtahedzadeh N, Neumann FA, Augustin M, Zyriax B-C, Harth V, Mache S. Das Gesundheitsverhalten von Pflegekräften – aktueller Forschungsstand, Potenziale und mögliche Herausforderungen. Pravent Gesundh. 2021;16(1):16-20. <https://doi.org/10.1007/s11553-020-00792-y>.

74. García-Iglesias JJ, Gómez-Salgado J, Apostolo J, Rodrigues R, Costa EI, Ruiz-Frutos C, et al. Presenteeism and mental health of workers during the COVID-19 pandemic: a systematic review. Front Public Health. 2023;11:1224332. <https://doi.org/10.3389/fpubh.2023.1224332>.

75. Fischer T. Home care in Germany during the COVID‐19 pandemic: A neglected population? J Nurs Scholarsh. 2023;55(1):215-25. <https://doi.org/10.1111/jnu.12851>.

76. Rohwer E, Mojtahedzadeh N, Harth V, Mache S. Stressoren, Stresserleben und Stressfolgen von Pflegekräften im ambulanten und stationären Setting in Deutschland. Zentralbl Arbeitsmed Arbeitsschutz Ergon. 2021;71(1):38-43. <https://doi.org/10.1007/s40664-020-00404-8>.

77. Kunzler AM, Chmitorz A, Röthke N, Staginnus M, Schäfer SK, Stoffers-Winterling J, et al. Interventions to foster resilience in nursing staff: A systematic review and meta-analyses of pre-pandemic evidence. Int J Nurs Stud. 2022;134:104312. <https://doi.org/10.1016/j.ijnurstu.2022.104312>.

78. Engelmann P, Toussaint A, Addo MM, Brehm TT, Lohse AW, Weigel A, et al. Predictors of somatic symptom burden in healthcare professionals during the COVID-19 pandemic: an 8-week follow-up study. Journal of Mental Health. 2023;32(6):1111-21. <https://doi.org/10.1080/09638237.2022.2069709>.

79. Dürr L, Forster A, Bartsch CE, Koob C. Anforderungen, Ressourcen und Arbeitsengagement Pflegender während der zweiten Welle der COVID-19-Pandemie. Pflege. 2022;35(1):5-14. <https://doi.org/10.1024/1012-5302/a000820>.

80. Hartog CS, Hoffmann F, Mikolajetz A, Schröder S, Michalsen A, Dey K, et al. Non-beneficial therapy and emotional exhaustion in end-of-life care : Results of a survey among intensive care unit personnel. Anaesthesist. 2018;67(11):850-8. <https://doi.org/10.1007/s00101-018-0485-7>.
