# Supplementary material for: Health status, health behavior and perceived stress of nursing staff in Germany: a scoping review
Source: BMC Nurs. 2026 Jan 9;25:97. doi: 10.1186/s12912-025-04282-4 (PMC12849386; doi:10.1186/s12912-025-04282-4)
Supplement: Supplementary file 4 — Supplementary Material 4: Additional file 4: File format: .docx. Title of data: Data extraction instrument based on Peters et al. Description of data: The data extraction form developed for this review, adapted from the framework by Peters et al [file 12912_2025_4282_MOESM4_ESM.docx]

# **Additional file 4: Data extraction instrument based on Peters et al. [1]**

| **Study details and characteristics** | |
| --- | --- |
| Author(s) |  |
| Year of publication |  |
| Country of origin (where was the study conducted) |  |
| Participant characteristics: Age, gender, number |  |
| Study design |  |
| Study methods |  |
| **Important results related to the research question of the scoping review** | |
| Concept of health |  |
| Concept health behavior |  |
| Concept work-related stress |  |

**References:**

1. Peters MDJ, Godfrey C, MCInerney P, Munn Z, Tricco AC, Khalil H. Chapter 10. Scoping reviews (version 2020). 2020. In: JBI manual for evidence synthesisis. https://doi.org/10.46658/JBIMES-24-09.
