# Supplementary material for: Health status, health behavior and perceived stress of nursing staff in Germany: a scoping review
Source: BMC Nurs. 2026 Jan 9;25:97. doi: 10.1186/s12912-025-04282-4 (PMC12849386; doi:10.1186/s12912-025-04282-4)
Supplement: Supplementary file 5 — Supplementary Material 5: Additional file 5: File format: .docx. Title of data: Detailed study description and results. Description of data: A table presenting the detailed characteristics and findings of the included studies [file 12912_2025_4282_MOESM5_ESM.docx]

**Additional file 5: Detailed study description and results**

| **Reference**  (author, year, reference number) | **Country of origin**  (where the study was conducted) | **Study design** | **Survey period** | **Measuring instruments/Data collection methods** | **Sample** | **Related Concept** | **Outcomes/measures** |
| --- | --- | --- | --- | --- | --- | --- | --- |
| Studies without reference to the COVID-19 pandemic | | | | | | | |
| Körber M, Schmid K, Drexler H, Kiesel J (2018) [1] | Germany (not specifically defined region) | Cross-sectional study conducted as an anonymous written survey of 3 clinics to record the subjective workload. | not specified | A specially designed questionnaire assesses various aspects of daily professional life, including workload and related stress | Sample size*:  n = 191 nurses  Age: not reported  Gender: Male = 10%, Female = 90%,  *Subgroup of a cross-setting study | Work-related stress /Health | Workload   - 53% feel under time pressure every day or several times a week - 61% are dissatisfied with their remuneration - 57% are dissatisfied with opportunities for professional development - 31% state that their leisure time is negatively affected by work - 30% report difficulties in balancing work and family life - 28% perceive overtime as a significant burden   Health   - 27% feel mentally stressed daily or several times a week - 25% feel exhausted/tired daily or several times a week |
| Graeb F (2019) [2] | Germany (Stuttgart region) | Cross-sectional study conducted as an anonymous written survey to record ethical conflicts and moral distress in intensive care units. | April to June 2017 | Literature-based individual items that assess aspects of everyday professional life, workload and associated stress, the concrete experience of ethical and moral conflicts including their causes, factors for or against institutional ethical support services, demographic information | Sample size*: n = 262 nurses in intensive care units (ICU)  Age: ≤ 30 years = 26%, >30 ≤ 40 years = 26%, 41 ≤ 50 years = 27%, >50 years = 19,5%  Gender: Male = 20.6%, Female = 78.2%, no response = 1.1%  *Subgroup of a cross-setting study | Work-related stress | Workload:   - 79% report a high workload - 48% often work overtime - 71% do not experience clear agreements on work processes in the event of staff shortages - 62% see a patient risk due to insufficient staffing - 41% feel strongly or very strongly burdened by intrapersonal conflicts. - 31% feel strong or very strong stress from interpersonal conflicts - 63% experience moral stress as a moderate to severe burden - 67% complain about unclear decision-making procedures - 61% cite a lack of time and personnel as a stress factor - 53% see problems in communicating with doctors |
| Breinbauer M (2020) [3]  Breinbauer M (2020) [3] | Germany (Rhineland-Palatinate) | Cross-sectional study conducted as an online survey to record working conditions and workloads in nursing. | November 2016 to February 2017 | - Items based on the Maslach burnout inventory - Literature-based individual items to measure workload, coping strategies, and job satisfaction | Sample size: N = 830 nurses  Age:  20 to 29 years = 15%  30 to 39 years = 20%  40 to 49 years = 23%  50 to 59 years = 34%  ≥60 = 8%  Gender: Male = 25%, Female = 75% | Work-related stress  Health | Working hours and workload:   - Almost three-quarters of nurses report that there is insufficient staffing in their work area to adequately complete the required tasks - 43% of respondents stated that they frequently must cover extra shifts or take over duties from colleagues - On average, the respondents worked approximately 61 hours of overtime (standard deviation [SD] 52.8) in the last six months before the survey - There is a tendency for nurses in outpatient (70 hours) and inpatient/semi-residential care (69 hours) to be more frequently affected by overtime than those working in acute care (58 hours) - Lack of time is a central issue in acute care as well as in inpatient and semi-residential care settings - 75% of nurses in acute care and 70% in inpatient care report not having sufficient time for patient care. From the nurses perspective, too much time is spent on administrative tasks at the expense of core nursing duties   Gratification:   - 80% of respondents considered their remuneration to be inadequate   Subjective perception of stress:   - 52% of nurses reported feeling highly burdened by time pressure and increased workload - 30% of nurses reported high levels of stress due to conflicts with supervisors - 27% of nurses reported being heavily burdened by physically demanding tasks (e.g., lifting, carrying, repositioning patients) - 26% of nurses felt significantly burdened by a lack of appreciation and recognition - 26% of nurses reported being strongly affected by conflicts among colleagues - 25% of nurses experienced considerable strain due to shift work - 22% of nurses felt highly burdened by overtime hours   Impact on retention in the profession:   - Two-thirds of respondents have considered leaving the nursing profession prematurely - This is particularly evident among younger respondents (aged 30 to 39), who are already contemplating an early exit from the profession - The main reasons cited were staff shortages, high psychological and physical demands, and a lack of prospects for financial improvement   Impact on quality of nursing care:   - 41% of inpatient and day-care nurses stated that shift staffing with qualified staff is not sufficient to ensure nursing security for patient care - In acute care, around one in two nurses stated that the staffing of shifts with qualified staff is not sufficient to ensure nursing security for patient care   Self-perceived health status:   - On average, nurses rated their subjective health status as satisfactory   Specific health issues:   - 58% of nurses reported experiencing back, neck, or joint pain daily or frequently - 36% reported daily or frequent sleep problems - 35% reported feeling inner tension and restlessness on a daily or frequent basis - 27% reported being irritable daily or frequently - 25.% reported suffering from concentration problems daily or frequently - 21% reported experiencing stomach pain or digestive issues daily or frequently - 21% reported suffering from headaches daily or frequently   Burnout   - One third of respondents were identified as being at high risk of burnout - Nurses working in acute care and inpatient/semi-residential care settings appear to be more frequently affected by burnout compared to those in outpatient care   Relationships between workload and health:   - The higher the workload, the higher the risk of burnout - Higher job satisfaction is associated with lower perceived workload, a reduced self-assessed risk of burnout, and a more positive evaluation of coping strategies - The more helpful coping strategies are perceived to be, the lower the risk of burnout |
| Ehegartner V, Kirschneck M, Frisch D, Schuh A, Kus S (2020) [4] | Germany (nationwide) | Cross-sectional study conducted as an anonymous paper- and web-based expert survey on subjective work ability based on the Work Ability Index. | March to end of May 2017 | - Literature-based individual items to assess demographic information, and the need for training and prevention programs - Short form of Work Ability Index (WAI) | Sample size: N = 1,381 nurses and nursing assistants  Age: Mean = 40,1 years (SD = 12,0)  Gender: Male = 19%, Female = 81% | Work-related stress | Workload:   - The nurses surveyed estimate their subjective ability to work with an average WAI score of 37.2 (SD=7.1), which corresponds to a rather moderate ability to work   Need for prevention:   - Over 80% want training on stress management - Over 70% would like theoretical input on the topics of communication, conflict management and teamwork |
|  |  |  |  |  |  | Health | - 50% of the nurses stated that they had at least one medically diagnosed illness. - Diseases of the musculoskeletal system were reported most frequently (80%) - 39% reported diagnoses of cardiovascular diseases - 32% reported mental impairments - 30% reported accidental injuries - 26% reported respiratory diseases |
| Diehl E, Rieger S, Letzel S, Schablon A, Nienhaus A, Escobar Pinzon LC, Dietz P (2021) [5] | Germany (nationwide) | Cross-sectional study conducted as an anonymous paper and online survey to record the working conditions of nurses in general palliative care. | 2017 | - Parts of Copenhagen Psychosocial Questionnaire (COPSOQ) - Literature-based individual items to assess demographic information, occupational stress, health status, and personal resources | Sample size: N = 437 palliative care nurses  Age: Mean = 42.8 years (SD = 11.8)  Gender: Male = 10%, Female = 89% | Work-related stress /Health | Stress factors   - 54% of nurses said they felt burdened by caring for too many patients - 62% of nurses said they often/always have to work fast - 55% reported that their work was high/very emotionally demanding - 52% felt that patients who die alone were a burden - 45% found depressive patients stressful - 59% cited physical demands (lifting and carrying patients) as a stress factor - 54% found relatives who cause unrest to be stressful   Support and training needs   - 77% of the nurses surveyed found the support of specially trained palliative care nurses helpful - 25% of those surveyed wanted to expand their own palliative care skills but were not supported by their employer in this regard. - 11% reported that they felt overwhelmed with palliative care work |
| Diehl E, Rieger S, Letzel S, Schablon A, Nienhaus A, Escobar Pinzon LC, Dietz P (2021) [6] | Germany (nationwide) | Cross-sectional study conducted as an anonymous paper and online survey. Comparative Analysis of Burdens, Resources, Health and Well-Being of nurses in General and Specialized Palliative Care. | 2017 | - Parts of Copenhagen Psychosocial Questionnaire (COPSOQ) - Patient Health Questionnaire (PHQ-2) - Resilience Scale Questionnaire (RS-13) - A single question on back pain from the Robert Koch Institute Health Survey - Literature-based individual items to assess demographic information, and occupational stress | Sample size: N = 1753 (n = 437 general palliative care [GPC] and n = 1316 specialised palliative care [SPC])  Age GPC / SPC:  < 30 = 17% / 8%  30–39 = 24% / 18%  40–49 = 22% / 28%  ≥ 50 = 36% / 45%  Gender GPC / SPC: Male = 10% / 13%, Female = 90% / 87% | Work-related stress  Health | - Nurses in specialised palliative care (SPC) report higher emotional demands (Difference in Mean: 7.0) as well as greater stress from caring for patients (Difference in Mean: 9.9) and interacting with relatives (Difference in Mean: 6.1) - Nurses in general palliative care (GPC) report higher quantitative demands (Difference in Mean: 12.7) - SPC nurses more frequently report experiencing organizational and social support - SPC nurses also more often cite religiosity and spirituality, meaningfulness of work, self-reflection, physical activity, self-care, hobbies, professional detachment, gratitude from relatives, and positive thinking as helpful in managing work-related stress compared to GPC nurses   Professional loyalty:   - Nurses in GPC reported a higher intention to leave the profession than nurses in SPC (Difference in Mean: 7.8) - General practitioner nurses report a poorer state of health and report chronic back pain (52% vs. 38%) and major depressive disorder (12% vs. 3%) more often than SPC nurses |
| Diehl E, Rieger S, Letzel S, Schablon A, Nienhaus A, Escobar Pinzon LC, Dietz P (2020) [7] | Germany (nationwide) | Cross-sectional study conducted as an anonymous paper and online survey to record the effects of quantitative requirements in nursing. | 2017 | - COPSOQ - Quantitative Requirements Scale - RS-13 - Literature-based individual items to assess demographic information | Sample size: N = 1371 palliative care nurses  Age:  ≤ 39 years = 27%  40–49 years = 28%  ≥ 50 years = 45%  Gender: Male = 13%, Female = 87% | Work-related stress  Health | Resources:   - 44% of nurses said that meeting relatives after the death of patients helped them to better cope with professional demands - 68% of respondents felt valued in their work by their managers, while 27% received this recognition through their salary - 85% of nurses emphasized that they would not be able to do their job without a well-functioning team - 29% of the nursing staff had a low resilience, 21% a medium capacity and 50% a high resilience - Negative association between self-assessed health and quantitative requirements - Recognition through salary and a good work team act as moderators for self-assessed health - Positive correlation between burnout and quantitative requirements - Workplace engagement and a good work team reduced the negative impact of quantitative demands on burnout - Higher quantitative demands increased the likelihood of leaving the profession – the strongest increase was observed with a low degree of freedom - The intention to leave the profession increased with increasing demands – more so among nurses without recognition by superiors - Resources can mitigate the negative influence of quantitative demands on the intention to leave the profession |
| Schwarzkopf D, Rüddel H, Thomas-Rüddel DO, Felfe J, Poidinger B, Matthäus-Krämer CT, Hartog CS et al. (2017) [8] | Germany (nationwide) | Cross-sectional and multicenter paper-pencil survey on personal and work-related characteristics, perceived unfavorable treatment, burnout, and intent to quit among intensive care nurses and assistant and senior physicians. | not specified | - Literature-based individual items to assess demographic information - Safety Attitudes Questionnaire - Collaboration about Care Decisions Scale (4 Items version) - Subscale of the Maslach Burnout Inventory - 3-Item-Short form of Turnover Intentions Scale | Sample size*:  n = 574 intensive care nurses  Age (nurses):  < 30 years = 30%  30–39 years = 31%  40–49 years = 28%  ≥ 50 years = 11%  Gender (nurses): Male = 27%, Female = 73%  *Subgroup of a cross-setting study | Work-related stress /Health | - 50% of nurses reported an intention to leave their job - The perception of non-conducive (nonbeneficial) treatment is associated with increased emotional exhaustion (β = 0.34; p ≤ 0.001) and greater intention to leave the job (OR = 1.68; p ≤ 0.001) - In multivariate analysis, both nurses and resident physicians reported significantly higher levels of perceived non-conducive treatment than senior physicians - A poor work environment - particularly high workload and inadequate collaboration with other departments - predicts both emotional exhaustion and intention to leave (OR = 0.82; p = 0.034 for collaboration) - A moderation analysis showed that nurse-physician collaboration influences the perception of non-conducive treatment among nurses (β = –0.32) and residents (β = –0.49), but not among senior physicians (β = 0.04; p = 0.753). While nurses and residents typically follow decisions made by senior physicians, the latter are not necessarily reliant on collaboration with them in their own decision-making. |
| Werner NS, Bültmann M, Möckel L (2023) [9] | Germany (North-Rhine Westphalia) | Cross-sectional study conducted as an anonymous online survey to compare the psychological stress situation of inpatient vs. outpatient nurses. | November 2019 to January 2020 | - Short version of Perceived Stress Questionnaire (PSQ20) - Questionnaire on health-influencing workload in nursing - Questionnaire Psychosomatic Complaints in a Non-Clinical Context | Sample size:  n = 80 outpatient nurses of a single regional care provider  n = 78 inpatient nurses of a single regional care provider  Age: Mean = 44.11 years (SD = 12.84)  Gender: Male = 6%, Female = 94% | Work-related stress | - Outpatient nurses reported a higher workload compared to inpatient nurses (t(155) = 3.72, d = 0.59) - This was due to coordination and information problems such as a vague division of labor and work processes, role conflicts and time pressure, but also, due to psychophysical overload, which was accompanied by exhaustion due to a lot of work and the assumption of responsibility (η² = .13 for coordination/information; η² = .04 for psychophysical overload) |
|  |  |  |  |  |  | Health | - Outpatient nurses reported a higher subjective experience of stress than inpatient nurses (t(156) = 2.02, d = 0.32) - They reported more worries such as fears of the future or fear of failure and more frustration (η² = .04) - In addition, the outpatient nurses felt more tense and exhausted (η² = .03) as well as less secure and joyful (η² = .04) than colleagues who worked in inpatient care - In addition, outpatient nurses reported more psychosomatic complaints such as headaches, fatigue or musculoskeletal complaints compared to inpatient nurses (mean = 2.52 [SD = 0.73] vs. mean = 2.30, [SD = 0.69]) |
| Hartog CS, Hoffmann F, Mikolajetz A, Schröder S, Michalsen A, Dey K, Riessen R et al. (2018) [10] | Germany (nationwide) | Cross-sectional study conducted as an anonymous paper-based survey that investigated whether the work environment "End-of-life care" (EOLC) is associated with the perception of overtherapy or burn-out. | September 2015 to June 2016 | - Subscale of the Maslach Burnout Inventory - Literature-based individual items to assess demographic information, work environment, and perceived frequency of overtreatment | Sample size*: n = 325 nurses in ICU; n = 91 physicians  Age:  < 30 years = 32%  30–39 years = 29%  40–49 years = 22%  ≥ 50 years = 17%  Gender: Male = 30%, Female = 70%  *Subgroup of a cross-setting study | Work-related stress/ Health | - In this survey, the working environment was found to influence both the perception of non-beneficial treatment and the experience of stress - Collaboration in the end-of-life (EOL) context was negatively correlated with the perception of non-beneficial treatment (r = –0.46) and with emotional exhaustion (r = –0.19) - In this sample, deficits in interdisciplinary collaboration (β = –0,38), a subjectively perceived high workload (β = 0,17), and a higher number of weekend working days per month (β = 0,12) were significantly associated with a more frequent perception of non-beneficial treatment - Nurses reported experiencing non-beneficial treatment significantly more often than physicians (β = –0,19) - Emotional exhaustion, a key dimension of early-stage burnout, was particularly associated with high workload (β = 0,33) and stressful interactions with patients' relatives (β = 0,17) - In contrast, specialization in intensive care (β = –0,15) and perceived emotional support within the team (β = –0,24) were found to be protective factors against emotional exhaustion |
| Knape C, Teubner A, Benkenstein A (2018) [11] | Germany (Saxony) | Cross-sectional study conducted as an anonymous online survey to investigate the work situation with a rolling working time model in outpatient care. | November 2016 to January 2017 | - Work Ability Index - Literature-based individual items to assess demographic information, work environment, and occupational stress | Sample size: N = 39 outpatient nurses with a rotating shift schedule model  Age: not reported  Gender: Male = 10%, Female = 90% | Work-related stress | - 50% of respondents state that they rarely or never feel under time pressure, whereas only 13% experience this frequently or constantly - 61% of nurses report that they rarely or never have to care for too many clients; only 13% perceive this as a frequent or permanent burden - 56% of participants feel frequently or always sufficiently informed about their work tasks, while only 8% perceive a lack of information - 71% of respondents indicate that the wishes and needs of clients are often or always considered - 79% of nurses rarely or never fear legal consequences from making wrong decisions; only 11% experience this fear frequently or continuously - 95% report a very good subjective state of health - Overall, a large proportion of the nurses surveyed rated the work requirements, their satisfaction, the duty roster model, the team atmosphere, and the leadership behavior of the managers as positive and less stressful - Nevertheless, only a third of those surveyed can imagine continuing to carry out the job until retirement age - A high level of satisfaction with the rotating shift schedule model was significantly associated with positive team dynamics, supportive leadership, reduced psychological strain, a stable social work environment, and improved work–life balance |
| Isfort M (2017) [12] | Germany (nationwide) | Cross-sectional study to investigate the work situation, staffing, job and job satisfaction and patient care. | January to February 2017 | Literature-based individual items to assess demographic information, work environment, and occupational stress | Sample size: N = 2233 ICU  Age:  26–30 years = 21%  31–40 years = 29%  41-50 years = 20%  Gender: Male = 35%, Female = 65% | Work-related stress | - This study found that shift staffing in German intensive care units was predominantly above a nurse-to-patient ratio of 1:2 - Findings suggest that essential aspects of nursing care were more frequently left undone when higher nurse-to-patient ratios (≥1:3) were reported - Lower staffing goes hand in hand with higher intention to leave - All measured indicators of job and professional satisfaction showed significantly lower agreement in the group working under higher patient loads (≥1:3 ratio) |
| Mehlis K, Bierwirth E, Laryionava K, Mumm FHA, Hiddemann W, Heußner P, Winkler EC (2018) [13] | Germany (Munich) | Mixed-methods study to investigate the prevalence and intensity of moral stress (MD) as well as possible causes experienced by oncology physicians and nurses in situations of treatment limitation decision (DLT situations). | Not specified | - Moral Distress Thermometer (MDT) - Literature-based individual items to assess demographic information, work environment, and Nurses' involvement into decision‐making | Sample size*: n = 50 nurses and n = 39 physicians  Age (nurses): Mean = 34.2 years (SD = 8.8)  Gender (nurses): Male = 40%, Female = 60%  *Subgroups of a cross-setting study | Work-related stress | - Oncologists (67%) and oncology nurses (74%) experience moral stress (MD) in most cases of treatment limitations - Nurses report greater MD intensity than physicians (mean 2.3; SD 2.3 vs. mean 1.5; SD 1.4) even though the responsibility for end-of-life decisions lies with physicians   The causes of MD differ depending on the occupational group:   - Physicians experience MD primarily because of the challenging decision-making - Nurses experience MD through compassion for patients and an unmet need to participate in end-of-life decisions |
| Wagner A, Rieger MA, Manser T, Sturm H, Hardt J, Martus P, Lessing C et al. (2019) [14] | Germany (not specifically defined region) | Cross-sectional study conducted as an employee survey using a standardized, paper-based questionnaire. Comparative analysis of the perception of hospital staff (doctors and nurses) of psychosocial working conditions, leadership, patient safety climate and occupational safety climate. | April 2015 to July 2015 | - COPSOQ - Adaptation of Copenhagen Burnout Inventory (CBI) - Transformational Leadership Inventory (TLI short) - Hospital Survey on Patient Safety Culture (HSPSC-D) - TWINS Occupational Safety - Literature-based individual items to assess demographic information, and work environment | Sample size*:  n = 381 physicians  n = 567 nurses from 2 university hospitals  n = 19 other  Age and gender were not reported for the subgroup of nurses  *Subgroup of a cross-setting study | Work-related stress | Psychosocial working conditions:   - The study showed significant differences between the two occupational groups in 12 out of 17 scales - Physicians rated 9 scales significantly more positively, including: impact at work, degree of freedom, development opportunities, purpose of work, engagement, social relationships, and job satisfaction - Nurses rated 3 scales more positively: quantitative demands, work-privacy conflict, and social support - The results imply that doctors rated their psychosocial working conditions more positively overall than nurses   Management:   - Leadership quality and transformational leadership were rated moderately - Nurses rated leadership quality more positively than doctors, which may be related to more direct supervisor structures   Patient safety climate:   - Physicians rated 7 scales more positively, including staffing, teamwork, management support, and overall patient safety - Nurses rated 2 scales more positively: openness of communication and handovers and transitions - Poor working conditions for nurses may affect their perception of patient safety   Occupational health and safety climate:   - Physicians rated management more positively than nurses, while nurses rated their direct supervisors more positively - Nurses considered occupational health and safety measures to be more important than doctors and reported occupational risks more often   Potential for improvement:   - Measures to improve conditions for nurses are necessary, especially with regard to workload, role conflicts and work-life balance - Improvements in leadership quality appear necessary - It should be intensified in communication between management and nurses in order to promote trust and a safety climate |
| Lauxen O, Blattert B (2021) [15] | Germany (Wiesbaden, Offenbach/Main) | Qualitative study conducted using narrative interviews to investigate irritations and ruptures in the professional identity of these nurses during their induction. | November 2018 to January 2019 | Narrative interviews | Sample size: N = 14 immigrant nurses from hospitals  Age and gender were not reported. | Work-related stress | The study shows that international nurses in Germany experience irritations and fractures in their professional identity on four levels:  Action Level:   - Nurses from abroad rarely perform close-contact care in their home countries, which leads to confusion in Germany - The restriction of certain tasks (e.g., drawing blood, inserting feeding tubes) is perceived as a limitation of their professional role   Knowledge level:   - Foreign nurses are largely unfamiliar with nursing theories and the extensive documentation required in Germany - The high technical standards are seen positively, while bureaucratic requirements are perceived as burdensome   Normative Level:   - The high degree of patient autonomy in Germany contrasts sharply with the nurses’ home countries, where physicians’ instructions tend to dominate - Appreciation for nurses is often greater in their countries of origin than in Germany   Role expectations:   - Nurses are unsettled by the wide range of duties in Germany, which includes tasks perceived as "non-nursing" (e.g., serving meals) - High workload and lack of time for individual patient care lead to frustration |
| Schilgen B, Handtke O, Nienhaus A, Mösko M (2019) [16] | Germany (Hamburg) | Qualitative interview study according to the guidelines "Consolidated criteria for reporting qualitative research" (COREQ) to investigate work-related barriers and resources of migrant and local nurses in home care. | February to July 2017 | Interviews | Sample size: N = 48 outpatient nurses (n = 24 migrant nurse^1^ and n = 24 Autochthonous^2^)  Age:  ^1^ Mean = 42.9 years (SD = 12); ^2^ Mean = 45.2 years (SD = 9.8)  Gender:  ^1^Male = 12%, Female = 88%; ^2^Male = 17%, Female = 83% | Work-related stress | - Regardless of their origin or cultural background, nurses find time pressure, lifting patients, lack of recognition and the personal fates of clients stressful - In intercultural teams, different perceptions of care and behavior as well as a lack of communication make cooperation difficult - Language differences are a major stressor that hinders functioning team collaboration as well as a positive care-patient relationship - Nurses with a migrant background or from minorities are also often exposed to prejudice as well as verbal and sexual harassment by clients. Nevertheless, they do not address these experiences to superiors or colleagues - Personal contact with people, a respectful exchange with colleagues and managers as well as the experience of meaningfulness in the workplace contribute to better coping with professional stress |
| Gencer D, Meffert C, Herschbach P, Hipp M, Becker G (2019) [17] | Germany (Baden-Wurttemberg) | Cross-sectional study conducted as an anonymous paper-based survey to investigate the stresses and strains of palliative care nurses in their everyday work. | July to October 2015 | - Questionnaire on the Stress of Doctors and Nurses (FBAS1) - Scale "Mental Health" from the IRES-24 questionnaire - ALLBUS (A German General Social Survey) question on self-assessment of the state of health - Literature-based individual items to assess demographic information, and work environment | Sample size: N = 167 palliative care nurses  Age: Median (min-max) = 48 years (23-62)  Gender: Male = 10%, Female = 90%, | Work-related stress /Health | - The results show a high psychological burden on the study population: 28% of the nurses surveyed were "conspicuously" highly stressed, and a further 28% were even "severely" highly stressed - There was a significant difference between inpatient and outpatient palliative care nurses in terms of the type and intensity of stress - The total stress value determined was significantly worse for nurses in palliative care units compared to the outpatient setting - Higher stress levels led to lower satisfaction with the professional situation. - Dissatisfied nurses who had chosen scores of 5 or worse when asked about satisfaction were significantly more likely to report a poorer state of health - Nevertheless, the majority of nurses (64%) stated that they were in very good or good health - There was a clear correlation between psychological well-being and overall stress score: low psychological stress occurred in nurses with low overall stress, while high psychological stress was associated with high overall stress |
| Claaßen AC, Jeiler K, Martens D, Oetting-Roß C (2021) [18] | Germany (Münster) | Cross-sectional study conducted as an anonymous online survey to investigate the retention of the profession after completing nursing studies. | February 2019 | Literature-based individual items to assess demographic information, work environment, and job satisfaction | Sample size: N = 30 graduates of a BSc in Nursing at a university of applied sciences  Age: Mean = 26,4 years (Range: 20-34)  Gender: Male = 17%, Female = 83% | Work-related stress | - More than half of the respondents (55% of n = 29) expressed the concrete intention to change their professional situation - One reason for this was, among other things, the lack of opportunity to integrate study content into nursing practice (21% of n = 14) - In addition, 57% cited the desire to escape the challenging everyday care routine as a motive |
| Petersen J, Rösler U, Meyer G, Luderer C (2024) [19] | Germany (selected federal states) | Qualitative Study Conducted Using Semi-Structured Interviews to Investigate Moral Burdens in Home Care. | April to August 2023 | Semi-structured interviews | Sample size: N = 20 outpatient nurses  Age: Mean = 43.2 years (Range: 28-58)  Gender: Male = 30%, Female = 70% | Work-related stress | The research highlights that the tension between the ethical requirements of nursing and the practical limitations of the system leads to moral stress that affects both the health of nurses and the quality of patient care.  Situations that lead to moral stress:   - Inadequate care due to financial constraints or patients' refusal to accept support - Inability to protect one's own health and increased responsibility for all care - Conflicts between work and private life as well as between care ethics and the specifications of the benefit and billing system   Consequences of moral burdens:   - The nurses reported destructive cognitions (e.g., desire to leave the profession), negative emotions (e.g., helplessness, anger, sadness), and physical symptoms (e.g., stomach pain) - Long-term health consequences such as rumination, absenteeism, burnout and depression were also named   Systemic and individual challenges:   - Care system conditions that require objectively limited care time and economic pressure exacerbate moral burdens - Home care nurses are caught between the demands of the health service, the needs of patients, and their own family obligations   Coping:   - At the institutional level, nurses used team meetings and teamwork to deal with the moral burden - Exchange with colleagues was the main method of coping with moral stress (but showed limited effectiveness) |
| Ibenthal E, Hinricher N, Nienhaus A, Backhaus C (2024) [20] | Germany (nationwide) | Cross-sectional study conducted as an anonymous online survey to investigate the prevalence, severity and occupational associations of hand and wrist complaints among dialysis nurses. | October 2022 to March 2023 | - BORG-CR10 scale - Nordic Musculoskeletal Questionnaire (NMQ) - Symptom scale of the Boston Carpal Tunnel Questionnaire (BCTQ) | Sample size: N = 122 dialysis nurses  Age: Mean = 45 years (SD =12)  Gender: Male = 15%, Female = 85% | Health | - The results showed a high prevalence of hand and wrist complaints among dialysis nurses (59%) - 16% were diagnosed with carpal tunnel syndrome (occupational disease) - The severity of hand and wrist discomfort increased with the number of setups performed by dialysis nurses per day - The severity of symptoms increased significantly with the number of dialysis machine preparations performed by a dialysis nurse per day - Musculoskeletal complaints in the hands and wrists of dialysis nurses are primarily due to repetitive activities, while work equipment and the forces used are secondary factors |
| Helaß M, Greinacher A, Genrich M, Müller A, Angerer P, Gündel H, Junne F et al. (2025) [21] | Germany (not specifically defined region) | Qualitative interview study with focus group discussion to investigate the perception of stress and resilience by nursing staff and supervisors. | August to November 2018 | Literature-based semistructured interviews | Sample size: N = 50 nurses of a German maximum care hospital  Age: Mean = 50.1 years (SD =12.5)  Gender: Male = 48%, Female = 52% | Work-related stress | The study shows that nurses are exposed to high stresses, which are exacerbated by a lack of resources and structural deficits.   - Nurses experience a high workload due to short patient stays, high patient numbers, time pressure and increasing medical requirements, long working hours, on-call duty and lack of recovery periods - Nurses report little freedom of decision, a lack of a say and a high level of heteronomy - Lack of personnel and resources, high effort of documentation and economic pressure increase the stress - Poor interprofessional cooperation, a lack of appreciation by superiors and doctors, and a negative working atmosphere exacerbate psychological stress |
|  |  |  |  |  |  | Health | - 64% of nurses report physical complaints (e.g., back pain, exhaustion) - 85% of nurses cite psychological stress (e.g., burnout, emotional exhaustion) as the main problem |
| Raiber L, Kaluscha R, Tepohl L (2024) [22]  Raiber L, Kaluscha R, Tepohl L (2024) [22] | Germany (Karlsruhe) | Cross-sectional survey by means of a written survey and subsequent intervention (job-related over-45 health check) in the event of a negative Work Ability Index (WAI). | Beginning in spring 2018 | - WAI - Literature-based individual items to assess demographic information, and work environment - In the course of the health check, a general, social and occupational anamnesis was taken (including on resources and risk factors) | Sample size:  N = 202 nurses (of which n = 49 participants in the health check)  Age: between 45 and 52 years  Gender: Male = 10%, Female = 90% | Work-related stress | Stress factors:   - 59% of the nurses surveyed felt that organizational factors (e.g. understaffing, overcrowding) were stressful - 59% of those surveyed felt burdened by the high pace of work and constant time pressure - 57% of those surveyed felt that the inadequate remuneration for their work was stressful - 53% of respondents felt burdened by many different tasks that they have to cope with at the same time - 46% of those surveyed found physically strenuous activities (e.g. heavy lifting, carrying) to be stressful - 43% of those surveyed felt burdened by the lack of recognition of their profession - 43% of those surveyed found dealing with difficult patients stressful - 43% of respondents felt burdened by weekend work - 42% of those surveyed found dealing with cognitively impaired patients stressful - 41% of respondents felt burdened by administrative activities - 40% of those surveyed found the lack of recovery breaks to be stressful - 40% of respondents felt burdened by dealing with suffering or dying patients - 36% of those surveyed found overtime stressful - 30% of those surveyed felt burdened by non-nursing activities - 36% of those surveyed found the constant shift change stressful   Assessment of the ability to work:   - 26% of nurses considered it very unlikely and 32% rather unlikely that they would work in the care sector until they reached retirement age   Subjective need for over-45 health check:   - 63% saw a personal need for the offer of an over-45 health check |
|  |  |  |  |  |  | Health | The nurses most frequently reported diseases or complaints of the musculoskeletal system (e.g. back, head and neck pain) and the psyche (e.g. inner restlessness, irritability, sleep disorders)   - 48% were severely or very severely affected by musculoskeletal disorders - 26% reported mental illness - 8% reported an existing cardiovascular disease - Other diseases played a smaller role - 17% saw their mental health as being severely endangered by their current professional activity and 11% as very strongly endangered - 12% saw their physical health as being severely endangered by their current professional activity, and 14% as very strongly endangered |
| Baumann A-L, Kugler C (2019) [23] | Germany (nationwide) | Cross-sectional study conducted as an anonymous online survey to investigate career prospects of graduates of undergraduate nursing courses. | June to July 2016 | Literature-based individual items to assess demographic information, work environment, and job satisfaction. | Sample size: N = 237 graduates of nursing courses qualifying for a profession  Age: Mean = 27.9 years (SD = 5.8)  Gender: Male = 15%, Female = 73%, no response 12% | Work-related stress | Situational satisfaction with qualification-related room for maneuver:   - 51% of respondents are very satisfied with their work content - There is a high level of dissatisfaction in the areas of income level (39%), use of qualifications (39%) and promotion opportunities (35%)   Satisfaction with the situational conditions:   - Two-thirds of those surveyed stated that they were very satisfied with job security and the working atmosphere - The other items were also rated as very satisfactory by the majority - The respondents experienced the most dissatisfaction in connection with the appreciation by superiors (20%) and the equipment of the workplace (18%)   Satisfaction with work-life balance:   - With the aspects related to work-life balance (leisure opportunities, working hours, work-life balance), around a third of those surveyed were not satisfied, medium or very satisfied   Group comparison of job satisfaction:   - Nurses with direct patient contact (health, pediatric nursing and geriatric care) are significantly less satisfied than those in extended nursing practice fields   Future prospects for nursing staff:   - 78% of those surveyed cannot imagine staying in their current field of practice in the long term - 54% want to look for a new position in the medium term - 23% plan to change at short notice - 31% are already in secondary studies - 40% plan to start a course of study   The results show that the majority of nurses with direct patient contact are dissatisfied with their work situation, especially in terms of income, career opportunities and use of their qualifications. Many nurses are looking for further training opportunities to improve their professional situation, and a large proportion are planning to change careers in the medium term |
| Seemann A-K, Fischer H (2017) [24] | Germany (not specifically defined region) | Qualitative interview study to investigate the working conditions of nursing service managers. | not specified | Interview | Sample size*: n = 100 managers of outpatient care  Age and gender were not reported.  *Subgroup of a cross-setting study | Work-related stress | - The managers see the greatest stress factors in dealing with relatives, bureaucracy, disorganized workflows and negative feedback - Time pressure plays a rather subordinate role - Short official channels and improved communication can make work easier |
| Weigl M, Schmuck F, Heiden B, Angerer P, Müller A (2019) [25] | Germany (not specifically defined region in southern Germany) | Multi-source and cross-sectional study that combined self-reporting methods with medical examinations by a physician to investigate the associations between staff shortages and cardiovascular health of hospital care providers. | Not specified (presumably before 2013) | - Literature-based individual items - Work Analysis Instrument for Hospitals - Self Report Version (TAA-KH-S) - Standardized medical examination - Cardiovascular information on the health parameters blood pressure and blood cholesterol[,](https://www.sciencedirect.com/topics/nursing-and-health-professions/cholesterol-blood-level) as well as the SCORE classification | Sample size: N = 273 nurses at a university hospital  Age: not reported for the overall sample  Gender: Male = 15%, Female = 85% | Work-related stress /Health | - Multivariate analyses revealed significant associations between perceived staff shortages and elevated blood pressure [OR = 1.60, 95% CI: 1.05–2.43] and elevated total cholesterol [OR = 1.42, 95% CI: 1.04–1.95] - In addition, LDL cholesterol levels were associated with staff shortages - In addition, there was a correlation between high autonomy and elevated cholesterol levels |
| Thomas Schramm TJ, Schröder H (2017) [26] | Germany (Bavaria) | Cross-sectional study (paper-based and online) to investigate burnout in the workplace of Bavarian nurses. | not specified | - Maslach Burnout Inventory (MBI) - Organizational Climate Measure (OCM) - Practice Environment Scale of the Nursing Work Index (NWI-PES) | Sample size: N = 561 nurses  Age: not reported for the overall sample  Gender: Male = 12%, Female = 88% | Health | - The data showed a burnout prevalence of 15% among the participants - Three hospital departments were considered to be exposed to burnout (anesthesiology, intensive care, surgery and internal medicine - It could be shown that the prevalence of burnout varies significantly between individual hospital departments - Each working environment and organizational climate dimension had a preventive effect on burnout in at least one subcategory (emotional exhaustion, depersonalization, personal performance) - Job satisfaction reduces burnout in all dimensions and promotes resilience and personal performance - Finally, it was shown that female nurses have higher chances of becoming a non-DP than their male colleagues |
| Korbus H, Hildebrand C, Schott N, Bischoff L, Otto AK, Jöllenbeck T, Schoene D et al. (2023) [27] | Germany (nationwide) | Cross-sectional survey conducted as an anonymous paper-based survey to examine the state of health, resources and work requirements in geriatric care. | August 2018 to February 2020 | - Slesina Questionnaire - Nordic Questionnaire - SF-12 - Screening Subscale (SSCS) of the Trier Inventory for Chronic Stress (TICS) - AVEM - Literature-based individual items to assess health-related resources | Sample size: N = 854 geriatric nurses  Age: Mean = 41.0 years (SD = 12.7)  Gender: Male = 19%, Female = 81% | Work-related stress /Health | - According to the participants, the physical and mental workload in geriatric care is extremely high, with 75% of nurses reporting chronic stress - The overall model showed that occupational and personal resources have a stronger influence on mental health than on physical health, while occupational demands influenced both areas of health equally - A pattern of behavior and experience that is hazardous to health is more strongly associated with a lower state of health than a pattern of behavior that promotes health - Results of the multi-group test showed that work-related behavioral and experiential patterns significantly influence the relationship between physical and mental health - Only 43% of the nurses had a health-promoting coping pattern |
| Weigl M, Schneider A (2017) [28] | Germany (not specifically defined region) | Cross-sectional study conducted as an anonymous paper-based survey to investigate the relationships between job characteristics, employee stress and self-perceived quality of care in emergency departments. | Not specified | - German version of Maslach Burnout Inventory - Literature-based individual items to assess demographic information, and work environment | Sample size*: n = 13 Nursing staff in an emergency department  Age and gender were not reported.  *Subgroup of a cross-setting study | Work-related stress | - Patient-related stress factors and time pressure were rated as high by the respondents - Compared to other occupational groups in the emergency sector, nurses worked less overtime (still at a high level) - The perception of unfavorable working conditions, in particular the inadequate perception of staffing, was accompanied by a lower perception of the quality of care - Supervisor support and autonomy have been identified as potential resources to cushion the burden |
|  |  |  |  |  |  | Health | - 54% of nurses surveyed scored above the cut-off for emotional exhaustion - 69% of nurses showed high irritation scores, indicating significant work stress - Perceived time pressure contributed to increased emotional exhaustion (a major component of burnout) and irritability |
| Vaupel C, Vincent-Höper S, Helms L, Adler M, Schablon A (2021) [29] | Germany (Schleswig-Holstein,  Hamburg, Hesse and Rhineland-Palatinate) | Cross-sectional survey conducted as an anonymous written survey to investigate how often sexual harassment and violence against employees in nursing and care professions occur. | Not specified | - Literature-based individual items to assess demographic information, and work environment - WHO Well-being Index - MBI | Sample size:  N = 901 employees with regular contact with people in need of care or support  Age: Mean = 42,6 years  Gender: Male = 20%, Female = 80% | Work-related stress | Stress factors:   - 63% of inpatient and 48% of outpatient nursing staff reported having been affected by nonverbal sexual harassment at least once in the last twelve months - 69% of inpatient and 71% of outpatient nursing staff experienced verbal sexual harassment or violence at least once during this period - 53% of inpatient and 51% of outpatient nurses said they had experienced physical sexual harassment or violence at least once in the last twelve months - In the last twelve months, 9% of inpatient nursing staff "often" and 13% "very often" thought about quitting - In the last twelve months, 6% of outpatient nursing staff "often" and 8% "very often" thought about quitting - 33% and 34% of respondents in the care sector were not aware of any measures against sexual violence |
|  |  |  |  |  |  | Health | Inpatient nursing staff:   - Exhaustion: 58% feel exhausted at least once a month - Depressive mood: 10% occasionally feel depressive feelings, 3% often to almost always - Psychosomatic complaints: 25% suffer from it at least every few weeks, 19% even every few days to almost daily - Positive well-being: 32% experience it sometimes, 13% rarely to almost never   Outpatient nursing staff:   - Exhaustion: 50% feel exhausted at least once a month - Depressive mood: 10% feel depressed occasionally to often - Psychosomatic complaints: 26% complain of it at least every few weeks, 17% even every few days to almost every day - Positive well-being: 36% experience it sometimes, 8% rarely to almost never   Associations:  Sexual harassment and violence in the workplace are significantly associated with psychological impairments, especially emotional exhaustion, depression and psychosomatic complaints |
| Schablon A, Wendeler D, Kozak A, Nienhaus A, Steinke S (2018) [30] | Germany (Bavaria, Berlin, Mecklenburg-Western Pomerania and North Rhine-Westphalia) | Cross-sectional survey conducted as an anonymous written survey to investigate the prevalence and consequences of aggression and violence against nurses in Germany. | 2017 | - Staff Observation Aggression Scale-Revised (SOAS-R) - Single items of the WAI - Parts of Copenhagen Psycho-Social Questionnaire (COPSOQ) - Literature-based individual items to assess demographic information, and work environment | Sample size:  N = 1984  n = 884 nurses  n = 413 peripheral health occupations (e.g., nurse aides, assistant caregivers, or staff without professional nursing qualifications)  n = 687 teachers, social workers, educators, special education professionals and other  Age and gender were not reported for the subgroup of nurses. | Work-related stress | - 80% of respondents reported experiencing violence within the past 12 months. Of those, 94% indicated verbal violence, and 70% reported physical violence. - Compared to outpatient care, working in hospitals, inpatient geriatric care or in occupational therapy/residential facilities for people with disabilities is associated with a higher risk of experiencing physical violence - Violent and aggressive acts mainly included insults, pinching and scratching, beatings and threats - Sexual harassment was reported in all areas, with elderly care having the highest incidence at 18% - Violent attacks are risk factors for reduced ability to work |
|  |  |  |  |  |  | Health | Consequences of violent attacks:   - Of the n = 1,522 individuals who had experienced verbal and/or physical violence and aggression in the past twelve months, one third reported that this had led to high levels of stress. - The highest levels of strain were reported by employees in hospitals (44%) and residential facilities for people with disabilities (40%), followed by those working in occupational therapy settings (33%). In inpatient and outpatient geriatric care, 27% reported feeling highly burdened as a result. - Most respondents reacted with anger, anger, fear, helplessness or disappointment - As a result, those affected became more cautious, attentive and tense, and enjoyed their work and dealing with patients/clients/residents less - Effective workplace preparation is associated with a significantly lower perceived stress odds ratio (OR) 0.6, 95% CI 0.4–0.8) |
| Isfort M, Rottländer R, Weidner F, Gehlen D, Hylla J, Tucman D (2018) [31]  Isfort M, Rottländer R, Weidner F, Tucman D, Gehlen D, Hylla J (2016) [32] | Germany (nationwide) | Cross-sectional survey conducted as an anonymous paper-based survey of managers to investigate developments in inpatient long-term care. | November 2017 to December 2017 | Literature-based individual items to assess demographic characteristics, health, and work environment factors | Sample size: N = 1067 nurses in leadership positions  Age and gender were not reported | Work-related stress | Workload:   - 55% report an increased workload from 2016 to 2017 due to challenging behaviors - 52% report an increased workload from 2016 to 2017 due to basic and treatment care requirements for residents - 52% report an increased workload from 2016 to 2017 due to time-consuming work with relatives/conflicts with relatives - 38% report an increased workload from 2016 to 2017 due to stepping in at freely scheduled times - 30% report an increased workload from 2016 to 2017 due to deaths/end-of-life care - 28% report an increased number of overtime hours |
|  |  |  |  |  |  | Health | Effects of the workload:   - 41% report an increase in sick days among nurses - 43% report an increased duration of illness among nurses - 31% of managers observe increasing disease severity |
| Petersen J, Melzer M (2022) [33] | Germany (nationwide) | Partial evaluation of the BIBB/BAuA Employment Survey 2018: Panel study based on guideline-based telephone interviews  (CATI survey) to investigate the stress and strain situation in outpatient care. Comparative analysis of the stress situation of outpatient care compared to  inpatient setting. | October 2017 to April 2018 | Self-developed survey instrument to assess demographic characteristics, health, and work environment factors | Sample size:  N = 1425 nurses in outpatient and inpatient care (n = 242 nurses in outpatient care; n = 1183 nurses in inpatient care)  Age:  15–34 years = 21%  35–54 years = 50%  >55 years = 29%  Gender: Male = 20%, Female = 80% | Health | The stress situation of employees in outpatient care (number of musculoskeletal and psychosomatic complaints) does not differ from that of nurses in inpatient areas. |
|  |  |  |  |  |  | Work-related stress | Differences in the load spectrum (outpatient vs. inpatient):   - Compared to the inpatient setting (37%), outpatient nurses (60%) say they have to comply more often with requirements for minimum quantity/service or time - Work in forced postures is more often necessary in outpatient care (45%) than in clinics and rehabilitation facilities (30%), but comparable to old people's and nursing homes (46%) - Outpatient nurses (33%) are more often exposed to the weather than employees in an inpatient setting (14-19%) - Outpatient nurses (71%) experience collegial help less often than employees in an inpatient setting (80-86%) |
| Rothgang H, Müller R, Preuß B (2020) [34]  Rothgang H, Müller R, Preuß B (2020) | Germany (nationwide) | Cumulative evaluation of the BIBB/BAuA employment surveys 2006, 2012 and 2018 (telephone survey) comparing workloads and workloads between nursing staff and other occupational groups. | 2006, 2012 and 2018 | Self-developed survey instrument to assess demographic characteristics, health, and work environment factors | Sample size*:  Employment survey (n = 2887 nursing staff**)  Age: Nursing staff aged 15 to 64  Gender information was not reported in the employment survey.  *Subgroup of a cross-setting study | Work-related stress | **Results of the Employment Survey**  Physical strain   - Nursing staff * (88-94%) often work standing up (47% in other cases). Vocations) - Nursing staff (51-76%) often lift and carry heavy loads (15% in other professions) - 31-45% often work in forced postures (11% in other positions). Vocations) - 44-59% of nurses work with microbiological substances (9% in other cases). Vocations) - In total, nursing staff (36–46%) are more frequently dissatisfied with physical working conditions compared to other professions (15%)   Psychological stress:   - 28-40% of nurses feel emotionally burdened by their work (13% in other cases). Vocations) - 35-56% find deadline and performance pressure stressful (32% otherwise). Vocations) - 21-28% often work to their limits (12% in other cases). Vocations) - 22-40% experience strict regulations on minimum performance as burdensome (12% in other cases). Vocations) - 28-53% experience frequent disruptions and interruptions as stressful (28% experience frequent disruptions and interruptions). Vocations) - 29-37% feel overwhelmed by the amount of work (20% in other cases). Vocations) - 47%-62% report that stress and work pressure have increased (42% in other cases). Vocations)   **Nursing staff = geriatric nurses, geriatric nursing assistants, nursing professionals, nursing assistants |
|  |  | Data analysis of insured data (BARMER data 2016 to 2018) | 2016 to 2018 | ICD-10 Codes | Insured data (n = 723,701 years of insurance) | Health | State of health:   - Nurses report pain in the musculoskeletal system more often than other professions - 58-62% report tiredness and exhaustion during or immediately after work (46% in other cases). Vocations) - 18-25% report skin irritation (8% in other cases). Vocations) - 33-46% suffer from sleep disorders (25% in other cases). Vocations) - 28-35% report feeling depressed (20% in other countries). Vocations)   **Results of insured data analysis**  Diagnoses:  Ten of the 30 most common diagnostic groups occur more than 20% more frequently among geriatric nurses and geriatric care assistants than in other areas. Occupations, including: back pain, high blood pressure, depression, obesity, asthma, disc damage, stress disorders, spondylosis, tobacco addiction and type 2 diabetes  Sick leave:  Nursing staff* (7% - 8%) are sick more often than other professions (5%)  Probability of occurrence of reduced earning capacity pension:  Nursing staff (4% - 6%) enter the reduced earning capacity pension more often than usual. Occupations (3%)  *Nursing staff = geriatric nurses, geriatric nursing assistants, nursing professionals, nursing assistants |
| Techniker Krankenkasse (2019) [35] | Germany (nationwide) | Analysis of insured data of the Techniker Krankenkasse. Based on data on incapacity for work and drug prescriptions of employed insured persons, the health situation of employees in nursing professions is examined in order to identify specific health burdens of this occupational group. | 2018 | ICD-10 Codes | Sample size:  n = 181,000 insured persons with a nursing or geriatric care profession  Age: Nursing staff aged 15 to 64 | Health | Sick leave:   - Absenteeism in the care sector is above average   (On average, 1.38 cases of incapacity for work and 22.9 days of incapacity for work per year of insurance, compared to 1.21 cases of incapacity for work and 14.9 days of incapacity for work per year of insurance for other years. Vocations)   - Employees in geriatric care professions have even higher values than employees in nursing   Diagnoses:   - Nurses suffer more frequently from mental disorders and diseases of the musculoskeletal system than those in employment (across all occupations, employees receive an average of 247 sick days per 100 insurance years (previous year) with mental disorders and 261 sick days per occupational disability. - 100 years of insurance with musculoskeletal disorders vs. nursing staff with 463 AU days per 100 VJ with diagnoses of mental disorders and 478 AU days with diseases of the musculoskeletal system - On average, nurses receive more medication and higher daily doses than other occupational groups |
| Knieps F, Pfaff H (2022) [36] | Germany (nationwide) | Cross-sectional survey (employee survey) carried out on behalf of the BKK umbrella organization as an online survey to investigate the workload and workload of nursing staff. | May to June 2022 | Self-developed survey instrument to assess demographic characteristics, health, and work environment factors | N = 6000 social security employees (no information on how many nurses)  Age and gender were not reported for the subgroup of nurses | Work-related stress  Health | **Results of the Employee Survey**  Load:   - 44% of nurses report a lack of appreciation of the nursing profession by society - 86% of nurses consider the nursing profession to be very demanding - 66% of nurses consider the salary to be insufficient - 21% of geriatric nurses and 26% of health and nursing staff are dissatisfied with the compatibility of work and family life (compared to 8% in other professions) - 91% of nurses find the nursing profession to be very physically demanding - 88% of nurses perceive the nursing profession as psychologically very stressful - Nurses generally rate job satisfaction worse than other Professions   State of health:   - 18% of geriatric nurses and 14% of health and nursing staff assess their current physical health as very poor (vs. 9% otherwise professions) - 17% of geriatric nurses and 13% of health and nursing staff rate their mental health as very poor (vs. 9% otherwise professions) - The coronavirus pandemic practiced among nurses compared to usual. occupational groups have an above-average negative impact on their physical and mental health. |
| Knieps F, Pfaff H (2022) [36] | Germany (nationwide) | Analysis of BKK policyholder data. Based on data on incapacity for work and drug prescriptions of employed insured persons, the health situation of employees in nursing professions is examined in order to identify specific health burdens of this occupational group. | BKK data reporting year 2021 |  | Sample size: n = 175,934 insured nurses | Health | **Results Data Analysis Health Insurance Data**  Disablement:   - Nursing staff have significantly higher rates of sick leave than employees overall, primarily due to mental and musculoskeletal disorders - On average, health and nursing staff each have approximately 2 more sick days than usual. Total number of employees per year - The duration of the case is also longer than with the usual. Employees   Diagnoses:   - Compared to working people, nursing staff suffer more often from mental disorders and diseases of the musculoskeletal system |
| Vollbracht B, Gorgels S (2024) [37];  Vollbracht B, Görgels S, Stuckert M (2023) [38];  Vollbracht B, Gorgels S, Hombücher V (2022) [39] | Germany (nationwide) | Occupational Health Index Care, an instrument for industry monitoring for geriatric and nursing care.  Evaluation of data from the statutory health and accident insurance, the German Pension Insurance Federation, the Socio-Economic Panel (SOEP) and Media Tenor from 2013 to 2022 | 2013 to 2022 | Self-developed survey instrument in 4 dimensions (resources, working conditions, ability to work and earn a living, media opinion climate) | Sample size*:  SOEP (n = 554 nursing staff)  Media Tenor (n = 2104 nursing staff)  Other data sources (sample size not specified)  Age and gender were not reported  *Subgroup of a cross-setting study | Work-related stress | The core results of the BeGX 2024 show that occupational health in geriatric and nursing care has fallen sharply since 2013, with the index reaching its lowest level since the survey began in 2022.  Dimension Ressources:   - Decline in job satisfaction - Decline in continuing education   Dimension working conditions:  Increasing number of overtime hours |
|  |  |  |  |  |  | Health | Dimensions of work and earning capacity:   - Increase in days of incapacity for work and many reports of suspected occupational diseases |
| Hildebrandt-Heene S, Dehl T, Zich K, Nolting H-D (2023) [40]  Dehl T, Hildebrandt-Heene S, Zich K, Nolting H-D (2024) [41]  Klie T (2024) [42] | Germany (nationwide) | Data analysis of billing data from the statutory health insurance of DAK-Gesundheit to investigate the health situation of employees in nursing professions. | 2017 to 2022 | - Self-developed survey instrument to assess demographic characteristics, health, and work environment factors - ICD-Codes | Sample size Employment survey: N = 7052 social security employees (no information on how many nurses)  Injured data:  N = 60062 insured nurses  Analysis of employment histories:  n = 18,115 insured nurses | Work-related stress | Physical strain   - Nursing staff feel strongly burdened by heat twice as often as the average employee (49% vs. 23% otherwise professions) - Nurses are also more likely to report health problems as a result of the heat (37%) |
|  |  |  |  |  |  | Health | Sick leave:   - Nursing staff are sick more often than other professions   State of health:   - High number of sick leave days in nursing professions due to musculoskeletal and connective tissue diseases, which indicates high physical stress in these professions. - Also high number of sick leave days due to mental and behavioral disorders, which indicates psychosocial stress.   Reduced earning capacity:   - Slight differences in the number of early retirements (6% nursing staff vs. 4% non-nursing staff) - Slight differences in the number of occupational and occupational disability (4% of nursing staff vs. 3% of non-nursing staff) |
| Reviews without reference to the COVID-19 pandemic | | | | | | | |
| Schaller A, Klas T, Gernert M, Steinbeisser K (2021) [43] | Germany (nationwide) | Systematic literature review to record health problems and experiences of violence among nursing staff in acute care hospitals, long-term care facilities and outpatient long-term care in Germany. | January 2010 to January 2021 | Review according to PRISMA | n = 29 studies  (Of these, n = 23 studies examined mental health, n = 12 physical health and n = 9 experiences of violence)  Of these 29 studies, n = 8 studies are included in this overview as individual studies [4, 7, 14, 17, 28-30, 34]. 1 more study [44] was identified in the Scoping Review, but excluded in full-text screening due to the age of data collection | Health | - In terms of physical health, musculoskeletal complaints were most frequently recorded - The included studies show medium to high levels of burnout and a high prevalence of burnout - The high prevalence of burnout is usually explained by the circumstances of the nursing profession (unfavorable working hours, the routine management of mandatory alternating shifts, work overload due to staff shortages, time pressure, interface problems with other professional groups and high social responsibility - The available results indicate that mental health problems are most common among nurses in acute care hospitals - In terms of experiences of violence, nursing staff in long-term care facilities seem to be affected most often |
| Bail C, Marquardt B, Harth V, Mache S (2025) [45]  Kräft J, Wirth T, Harth V, Mache S (2024) [46] | Germany (nationwide)  Germany (nationwide) | Systematic literature review on the prevalence and manifestation of technostress experiences in inpatient medical care  Cross-sectional study conducted as an anonymous online survey to record the experience of technostress. | 2013 to 2024  April 2023 to June 2023 | - Subscale of the German version of the Digital Stressors Scale - Techno-Invasion in derivative of Ragu-Nathan's Technostress Creators Scale - Literature-based individual items - Subscale: Employee support for external assessment by employees of the HoL instrument by Franke et al. - Subskale of Copenhagen Burnout Inventory (CBI) - Work-Family Conflict Scale von Netemeyer et al. | 1 concept-relevant study (Kräft et al.)  Sample size: N = 239 nurses from hospitals  Age:  20–29 years = 14%  30–39 years = 21%  40–49 years = 30%  50–59 years = 28%  ≥ 60 years = 7%  Gender: Male = 26%, Female = 73% | Work-related stress/ Health | see Kräft et al.  The use of digital information and communication technologies (ICT) can be associated with increased technostress for nurses, which in turn can be associated with health consequences.   - Significant positive associations were found between technological invasion and health outcomes (emotional exhaustion) - Health-oriented leadership can mitigate these effects   Medical software such as the electronic patient record or decision support systems were not taken into account, which means that it can be assumed that the perception of technostress will continue to increase with increasing mechanization |
| Wirth LM, Ruppert N, Büscher A, Hülsken-Giesler M (2022) [47] | Germany (not specifically defined region) | Scoping Review for the Investigation of Occupational Health and Safety and Health Promotion in the Context of Personnel Assessment. | January 2015 to April 2020 | Not further specified | 1 context-relevant study [48] with Sample size*: n = 351 nurses of a university hospital  Age (min/max): 41.1 years (24/61)  Gender: Male = 17%, Female = 83%  *Subgroup of a cross-setting study | Work-related stress/  Health | - There are correlations between the objectively measured workload of the nurses and the perceived work-related psychosocial stress/strain - Overall, the findings support the hypothesis that an increased objective workload can lead to more work-related stress and strain, which in turn affects the well-being of staff and affects patient outcomes |
| Research related to health promotion interventions | | | | | | | |
| Bernburg M, Groneberg D, Mache S (2020) [49] | Germany (not specifically defined region) | Two-arm randomized control study (exploratory pilot study) regarding an intervention for mental health. | not specified | - Perceived Stress Questionnaire (PSQ) - COPSOQ - MBI - Emotion Regulation Skills Questionnaire (ERSQ-27) | Sample size*: n = 94 nurses in hospital departments  Age IG: Mean = 23.1 years (SD = 2.5)  Age CG: Mean = 23.6 years (SD = 2.4)  Gender IG: Female = 78%  Gender CG: Female = 71%  *Subgroup of a cross-setting study | Health | - Nurses in the intervention group showed a significant reduction in perceived work stress at time points T1 (F = 16.62, p < 0.001), T2 (F = 12.02, p < 0.001), and T3 (F = 9.42, p < 0.01), with large to medium effect sizes (T1: d = 1.0; T2: d = 0.7; T3: d = 0.6). - In addition, significant improvements were found in emotional exhaustion and in specific emotion regulation skills (p < 0.05), with small to medium effect sizes (d = 0.3–0.5) - The intervention was evaluated with high satisfaction scores - Training in mental health self-care can be a supportive approach for young nurses - However, replication studies are needed |
| Jenner SC, Djermester P, Oertelt-Prigione S (2022) [50] | Germany (Berlin) | Qualitative study conducted as semi-structured interviews to shed light on the breadth of prevention options against sexual harassment in the university medical context from the perspective of employees. | April to November 2015 | Semi-structured interviews | Sample size*: n = 15 nurses  Age:  20–29 years = 20%  30–39 years = 20%  40–49 years = 33%  50–59 years = 26%  Gender:  Female = 100%  *Subgroup of a cross-setting study | Work-related stress | The study highlights the importance of combining individual and system-wide measures to prevent sexual harassment  Prevention measures outlined by the participants:   - Personal safety measures and individual protection strategies towards patients, colleagues and superiors - Policies/Workplace Policies - Structured complaint and reporting procedures - formal training opportunities - Strategies for organizational development and leadership |
| Becker A, Angerer P, Weber J, Müller A (2020) [51] | Germany (Paderborn region) | Randomized control trial to investigate the long-term effects of work-related psychosocial coaching intervention compared to physiotherapy alone. | March to October 2016 | - Screening examination according to Spallek - Elements of systematic orthopedic medical examinations according to Cyriax - NMQ - Subscales (pain intensity, pain-related impairment) of the West Haven–Yale Multidimensional Pain Inventory - WAI - German version of the irritation scale according to Mohr - MBI | Sample size: N = 44 nurses from hospitals  Intervention group (IG): n = 24; Control group (CG): n = 20  Age: Mean = 43.98 years (SD = 9.59)  Gender: Male = 14%, Female = 86% | Health | - Study provides only marginal evidence that a combined intervention of work-related individual psychosocial coaching and physiotherapy is superior to standard treatment (physiotherapy only) for the prevention of non-specific musculoskeletal disorders in the long term - There is evidence that the combined intervention can support the increase in spinal mobility - However, a clear long-term effect of the intervention on MSK could not be demonstrated |
| Roth M, Altmann T (2024) [52] | Germany (North-Rhine Westphalia) | Evaluation study in longitudinal design with four measurement points to investigate the interplay of acceptance and effectiveness of a training to reduce burnout and distress symptoms (empCARE). | not specified | - Symptom-Checklist (SCL-90-R) - CBI - HEXACO-60 Modell - Toronto Empathy Questionnaire (TEQ) - Literature-based individual items to assess the acceptability of and satisfaction with the training | Sample size: N = 309 nurses from university hospitals (IG: n = 172; CG: n = 137)  Age IG: Mean = 39.9 years (SD = 10.9)  Age CG: Mean = 39.3 years (SD = 11.3)  Gender IG/CG: Female = 81%/82% | Work-related stress /Health | - Significant reduction of psychological stress and burnout in the intervention group - Long-term effects measurable over 12 months, but rather moderate effect sizes - No correlation between effectiveness and acceptance could be found - Acceptance ratings depend more on personality factors than on the success of the intervention |
| Research related to health promotion interventions with reference to the COVID-19 pandemic | | | | | | | |
| Berendonck C, Caspar R, BR M, Hobe M (2019) [53] | Germany (not specifically defined region) | Cluster randomized trial in 20 long-term care facilities to implement person-centered mini-intervention plans for dementia patients. | not specified | - Screening tool for occupational stress in the social sector (BHD) - modified version of Task and Job Analysis Tool – Residential LTC Version (TAA-A) | Sample size: N = 180 nursing staff from long-term care facilities  Age IG: Mean = 41.8 years (SD = 10.2)  Age CG: Mean = 38.5 years (SD = 11.9)  Gender IG/CG: Female = 88%/85%, Male = 12%/15% | Work-related stress | Nursing intervention (DEMIAN) in routine care and its impact on job satisfaction, motivation, and workload of nursing staff:   - The implementation of person-centered mini-intervention plans for people with dementia can lead to a reduction in time pressure at the workplace and to increased job satisfaction |
| Hoffmann A, Pilger S, Olbrecht T, Claassen K (2023) [54] | Germany (not specifically defined region) | Qualitative research design to investigate an online intervention of positive psychology. Guideline-based interviews were qualitatively analyzed using Mayring's summary qualitative content analysis. | Participant acquisition 5 to 25 July 2021 | Problem-centred partially standardized guided interviews | Sample size: N = 6 nurses from hospitals (min. 29 years to max. 33 years)  Age (min/max):  23 to 39 years  Gender: Male = 17%, Female = 83% | Health | Online-Intervention in positive psychology:   - The promotion of self-management skills with the help of positive psychology techniques has the potential to strengthen mental resilience and reduce the long-term effects of stress, burnout and depressive moods. It can also contribute to increasing job and life satisfaction as well as general well-being - Follow-up studies are needed to verify this assumption |
| Ell J, Brückner HA, Johann AF, Steinmetz L, Güth LJ, Feige B, Järnefelt H et al. (2024) [55] | Germany (Southern Germany) | Randomized controlled trial investigating the effectiveness of digital cognitive behavioral therapy for insomnia in a population suffering from shift work disorder with insomnia. | October 2021 to November 2022 | - One-week sleep diary - one-week actigraphy - Reduced Morningness–Eveningness Questionnaire (rMEQ) - Insomnia Severity Index (ISI) - Beck Depression Inventory-II (BDI-II) - State Trait Anxiety Inventory (STAI) - Perceived Stress Scale-10 (PSS-10) - Work Ability Single-Item-Question (WAI) - Subscales of the Recovery Experience Questionnaire (REQ) | Sample size: N = 46 nurses with shiftwork disorder  Age: Mean = 39.7 years (SD = 12.1)  Gender: Male = 20%, Female = 80% | Health | - The intervention group showed a significant reduction in the severity of insomnia compared to the control group - Improvements were observed in sleepiness, dysfunctional ideas about sleep, sleep effort, sleep efficiency, and latency to fall asleep - Mental health (depressive symptoms, anxiety, cognitive irritability) and ability to work also improved significantly - Future research should be done with larger samples and active control conditions |
| Roth C, Wensing M, Breckner A, Mahler C, Krug K, Berger S (2022) [56] | Germany (Baden-Wurttemberg) | A qualitative study (semi-structured face-to-face or telephone interviews) to investigate the perception of German nurses with regard to push and pull factors for leaving or remaining in the profession. | September 2019 to August 2020 | Semi-structured face-to-face or telephone interviews | Sample size: N = 21 nurses from hospitals  Age: Mean = 40.4 years (SD = 11.4)  Gender: Male = 19%, Female = 81% | Work-related stress | Factors contributing to the decision of nursing staff to leave the profession:   - Limited opportunities for career advancement - Intergenerational differences within the workforce - Negative societal perception of the nursing profession - High levels of occupational stress and workload   Factors that may enhance retention of nursing staff within the profession:   - A strong sense of professional pride - Improved remuneration - Increased recognition and appreciation - Advancement of professionalization within the field - Enhancement of the public image of the nursing profession |
| Lützerath J, Bleier H, Gernert M, Schaller A (2024) [57] | Germany (North-Rhine Westphalia) | Process evaluation based on the guidelines of the Medical Research Council for the implementation and reporting of process evaluations of the implementation of workplace health promotion in nursing. | Implementation of the Workplace health promotion (WHP) approach took place between April 2021 and October 2022 | Process evaluation based on the guidelines of the Medical Research Council for the implementation and reporting of process evaluations | Sample size: N = 432 nurses  Age and gender were not reported. | Work-related stress /Health | Demand and scope of WHP interventions:   - The measures implemented have been strongly adapted to the respective context and the available resources of the care facilities - In facilities with an already established health promotion system, measures were often shortened, while in facilities with higher levels of sick leave, they were implemented with fewer participants - Team building and stress management trainings were in greater demand regardless of the context - Relaxation offers and action days were the most frequently used measures, but served primarily to raise awareness and less to change behavior in the long term   Evaluation by the nursing staff:   - Measures were predominantly rated as useful and of (very) good quality - Important factors influencing satisfaction were a good announcement of the measures, the fit of the interventions to shift times and personal communication in addition to e-mail/bulletin boards   Result:   - A high workload can inhibit participation in WHP measures - Measures were assessed positively, but the focus was more on raising awareness rather than sustainable change |
| Riedl EM, Perzl J, Wimmer K, Surzykiewicz J, Thomas J (2024) [58] | Germany (not specifically defined region) | Two ecological momentary intervention studies (Study 1: Break Study; Study 2: After-Work Study) examining whether short audio-guided mindfulness meditations promote recovery during breaks and psychological detachment after work. | Study 1: May 2021 and July to August 2022  Study 2: December 2022 to January 2023 | - Literature-based single item analysis - Short scale by Wilhelm and Schoebi - Four-part scale of the Recovery Experience Questionnaire - Sustained Attention to Response Task | Sample size study 1: N = 38 geriatric nurses  Age: ≤45 years = 40%; >45 years = 60%  Gender: Male = 13%, Female = 87%  Sample size study 2: N = 26 hospital nurses  Age: ≤45 years = 44%; >45 years = 56%  Gender: Male = 12%, Female = 88% | Work-related stress /Health | - In both studies, the short mindfulness meditations were associated with a better recovery experience (recovery after the breaks, psychological distance) - Mediated by the recovery benefit, break and after-work meditations were associated with higher valence and rest after breaks and after sleep - Only under certain limitations did mindfulness meditations predict a lower rate of attention deficits - Short mindfulness meditations, during the breaks, could have a positive effect on attention after the break, but not independently of the self-reported mood variables - Feasibility of break meditations in care currently limited in view of the highly problematic status quo of break times in geriatric care |
| Reviews that deal with health promotion interventions | | | | | | | |
| Schaller A, Gernert M, Klas T, Lange M (2022) [59] | Germany (nationwide) | Systematic literature search on measures of workplace health promotion in nursing with presentation of results according to the RE-AIM framework. | January 2010 to January 2021 | Review according to the RE-AIM framework | n = 11 The study  (5 randomized controlled trials; 3 quasi-experimental studies; 3 single-group longitudinal studies) | Health | Workplace health promotion measures (WHP interventions) for nursing staff:   - Despite its social relevance, there are only a few studies on WHP interventions for nurses - No identified intervention study on the prevention or handling of experiences of violence - No studies on health promotion in the outpatient setting - The most common health goal of WHP interventions was mental health, but often without taking into account care-specific stresses (e.g., musculoskeletal complaints, violence) - According to the RE-AIM criteria, the reporting of most studies had several limitations, in particular a lack of reporting on implementation and acceptance - Most studies did not show a statistically significant effect on the respective endpoints (effectiveness) - Four studies reported results on maintaining effectiveness, suggesting continued effectiveness |
| Investigations with data collection period during the COVID-19 pandemic | | | | | | | |
| Early phase of the COVID-19 pandemic before the official declaration of the pandemic | | | | | | | |
| Bölsch-Peterka R, Thielmann B, Nübling M, Böckelmann I (2024) [60] | Germany (Saxony-Anhalt) | Cross-sectional study conducted as an anonymous online survey to record stress structures of outpatient nurses in 2 age groups. | August 2019 to January 2020 | - Short version of COPSOQ - Literature-based individual items to assess mobile and flexible work | Sample size: N = 33 outpatient nurses  Age and gender not reported | Work-related stress /Health | - Outpatient care is characterized by its mobile way of working and poses special challenges compared to inpatient care (lone work, changing weather conditions, increased risk of accidents in road traffic, changing locations) - Older nurses (≥45 years) usually have higher stress responses than younger employees - Younger nurses (<45 years), on the other hand, perform worse on items on their sense of community |
| Studies attributable to the first COVID-19 wave (March to May 2020) and the summer plateau (May to September 2020) [61] | | | | | | | |
| Helaß M, Maatouk I (2024) [62] | Germany (nationwide) | Cross-sectional study conducted as an anonymous online survey to estimate the prevalence of burnout among oncological nurses. | April to July 2020 | - 16-Items Oldenburg Burnout Inventar (OLBI) - Literature-based individual items to assess demographic characteristics, and work environment factors | Sample size: N = 83 oncological nurses  Age: Mean = 40.3 years (SD = 10.7)  Gender: Male = 17%, Female = 83% | Health | - Using a cut-off value of MExh > 2.5 (burnout criterion), the prevalence of burnout in the sample was 53% - 21% of respondents showed a high level of disengagement – defined as a high degree of psychological distance from one’s work, expressed through negative attitudes and behaviors towards the work itself - The risk of a high disengagement ratio was three times higher among nurses over 50 years of age than among nurses under 50 - 18% of respondents showed a high risk of psychological distress |
| Koskinen S, Brugnolli A, Fuster-Linares P, Hourican S, Istomina N, Leino-Kilpi H, Löyttyniemi E et al. (2023) [63] | Finland, Germany, Lithuania, Spain | Cross-national cross-sectional study conducted as an online survey to investigate the job satisfaction of newly graduated nurses (NGNs). | February 2019 to September 2020 | - Nurse Competence Scale (NCS) - Literature-based individual items to assess demographic characteristics, job satisfaction, and work environment factors | Sample size*: n = 63 NGNs from Germany  Age: Mean = 25.0 years (SD = 5.5)  Gender: Male = 25%, Female = 75%  *Subgroup of an international study | Work-related stress | - A positive correlation between satisfaction with the nursing education program and job satisfaction of NGNs - Satisfaction with nursing education influences satisfaction with the current job, the quality of care in the workplace and the nursing profession - Newly graduated nurses (NGNs) from Finland, Lithuania and Spain were more satisfied with the nursing profession than German NGNs - The satisfaction of Finnish, German, Lithuanian and Spanish NGNs with nursing education after graduation was statistically significantly associated with their job satisfaction, i.e. their satisfaction with their current job, the quality of care and the nursing profession |
| Heuel L, Lübstorf S, Otto A-K, Wollesen B (2022) [64] | Germany (nationwide) | Mixed-methods study using qualitative and quantitative data collection and analysis methods to investigate chronic stress, behavioral tendencies, and determinants of health behavior among nurses. | February to May 2020 | - 12-Item Screening Scale for Chronic Stress (SSCS) of the Trier Inventory for Chronic Stress (TICS) - AVEM-44 (work-related behavior and experience patterns) - Semi-structured interviews with questions about work stress, use of workplace health promotion programs and determinants of health behavior | Sample size: N = 43 nurses from different areas  Age: Mean = 40.1 years (SD = 13.3)  Gender: Male = 23%, Female = 77% | Work-related stress /Health  /Health behavior | - 84% of the nurses surveyed were chronically stressed - 49% showed unhealthy behavior patterns in the work environment - Stress levels were significantly higher among nurses with unfavorable work behavior patterns - Chronic stress was associated with higher perceptions of barriers to health-promoting behavior - Chronic stress was associated with lower levels of self-efficacy, which had a negative impact on health behaviors - Nurses with chronic stress showed lower resource use and poorer health behaviors - Burnout symptoms occurred in 6 of the 43 participants   Health-promoting behaviors and influencing factors:   - Self-efficacy was the strongest predictor of health-promoting behavior - Health-promoting resources (e.g., social support, private recreation) were used more frequently by non-stressed nurses - Organizational barriers such as high workloads, unfavorable shift work and the low attractiveness or lack of availability of health promotion programmes made it difficult to participate in health promotion measures - Dispositional character traits, sleep problems, team social support, diet and smoking, domestic duties and injury/illness were identified as personal barriers |
| Roth C, Berger S, Krug K, Mahler C, Wensing M (2021) [65] | Germany (Baden-Wurttemberg) | Multicenter cross-sectional study conducted as an anonymous paper-based survey to investigate the perception of safety culture, work-life balance, burnout and work requirements of internationally trained nurses and their guest nurses in German hospitals. | August 2019 to April 2020 | - German version of the SCORE questionnaire - Literature-based individual items to assess demographic characteristics, and work environment factors | Sample size:  N = 167 nurses from university hospitals (n = 64 internationally trained nurses and n = 103 host nurses)  Age:  Host nurses were distributed over all age categories, while internationally nurses tended to be younger (between 18 and 29 years (55%))  Gender host nurses: Male = 18%, Female = 81%  Gender international nurses:  Male = 25%, Female = 70% | Work-related stress | - Nurses who migrated to Germany were primarily looking for better working conditions, a higher standard of living and career development opportunities. - All nurses reported a high workload and a work-life balance in need of optimization - Nurses trained in Germany experienced a heavier workload (mean value 4.06 (SD 0.65)) (Engagement Assessment Tool) and a worse work-life balance (mean value 2.31 (SD 0.66)) than their internationally trained colleagues (mean value 3.67 (SD 0.81) and mean value 2.02 (SD 0.86)) |
|  |  |  |  |  |  | Health | - Nurses trained abroad reported a lower work-related burnout climate (mean 55.4 (SD 22.5)) than nurses in the host countries (mean 66.4 (SD 23.5), but still at a moderately high level - Internationally trained nurses with language skills of B1 perceived a higher burnout climate - It is possible that the abovementioned expectations of immigrant nurses will not be met, which in turn could have a negative impact on the integration process and their retention |
| Studies attributable to the second COVID-19 wave (September 2020 to February 2021) [61] | | | | | | | |
| Möckel L, Hönl A-K, Gräfe S, Jantz F, Werner NS (2022) [66] | Germany (nationwide) | Post hoc analysis of the data of a cross-sectional study conducted as an anonymous online survey to record pain, painkiller intake and mental health of intensive care nurses. | November 2020 to January 2021 | - Literature-based individual items to assess demographic characteristics, work environment factors, and the prevalence of sleep disorders - Depression, anxiety and stress scales (DASS-21) | Sample size: N = 432 intensive care nurses  Age:  20-29 years = 33%  30-39 years = 32%  40-49 years = 23%  50-59 years = 11%  >60 years = 1%  Gender: Male = 17%, Female = 83% | Health | - 58% of nurses reported sleep problems - Sleep problems were significantly associated with advanced age, particularly among those aged 50–59 years compared to those aged 20–29 years (OR: 2.05; 95% CI: 1.00–4.21), as well as with type of cohabitation, with nurses living in families showing significantly lower odds of sleep problems compared to those living alone (OR: 0.50; 95% CI: 0.27–0.93) - Sleep problems were also associated with higher levels of depressive symptoms (OR: 1.09; 95% CI: 1.06–1.12), anxiety symptoms (OR: 1.10; 95% CI: 1.06–1.14), and stress (OR: 1.09; 95% CI: 1.06–1.12) - No significant associations were found between sleep problems and body mass index or different shift work models |
| Hönl A-K, Jantz F, Möckel L (2023) [67] | Germany (nationwide) | Cross-sectional study conducted as an anonymous online survey to record pain, painkiller intake and mental health of intensive care nurses. | November 2020 to January 2021 | - Literature-based individual items to assess demographic characteristics, work environment factors, and the prevalence of sleep disorders - Depression, anxiety and stress scales (DASS-21) | Sample size: N = 432 intensive care nurses  Age:  20-29 years = 33%  30-39 years = 32%  40-49 years = 23%  50-59 years = 11%  >60 years = 1%  Gender: Male = 17%, Female = 83% | Health | - 57% said they suffered from pain (of any kind) - 37% suffered from recurrent - 19% of chronic pain - Among nurses with chronic/recurrent pain (n = 230), the lumbar spine (59%), the head (57%), the cervical spine (44%), the lower extremities (23%), the thoracic spine (20%), the gastrointestinal area (19%) and the upper extremities (17%) were most frequently affected - Significantly associated with chronic pain were body weight, and gender, - Significantly associated with recurrent pain were gender and the presence of sleep problems - 53% of painkillers consumed with chronic/recurrent pain (most common active ingredient: ibuprofen [82%]) - Nurses with chronic/recurrent pain showed significantly higher levels of depression, anxiety, and stress compared to those without pain. |
| Bruyneel A, Dello S, Dauvergne JE, Kohnen D, Sermeus W (2025) [68] | Six European countries (Belgium, England, Germany, Ireland, Norway and Sweden) | Cross-sectional study conducted as an anonymous online survey to estimate the prevalence and risk factors for burnout, missed care services and intentions to quit among nurses in intensive care and general wards. | November 2020 to July 2021 | - 12-Items version of Burnout Assessment Tool (BAT) - Literature-based individual items to assess demographic characteristics, work environment factors, and missing nursing care | Sample size:  N = 6655 nurses  n = 1641 from Germany (n = 534 intensive care nurses and n = 1107 general ward nurses)  Age and gender were not reported for the subgroup of German nurses | Work-related stress /Health | - The prevalence of burnout was significantly lower among nurses in intensive care units (27% vs. 30%) - Nursing absences were significantly less common among nurses in intensive care units (66% vs. 75%) - In both work environments and all countries, "comforting/talking to patients" was most often neglected - In all groups, "appropriate patient monitoring" was the most frequently missed clinical aspect - The intention to quit was similarly high in both groups (28% vs. 29%) - Nurses working in better work environments and experiencing lower workloads reported significantly lower levels of burnout and intention to quit compared to those in poorer environments with higher workloads - Country-specific analyses showed higher levels of burnout and intention to quit among nurses in Germany, Ireland, Scandinavia, and England compared to Belgium |
| Studies attributable to the third COVID-19 wave (March to June 2021) or a later COVID-19 wave [61] | | | | | | | |
| Laferton JaC, Schiller S, Conrad D, Fischer D, Zimmermann‐Viehoff F (2024) [69] | Germany (Potsdam) | Cross-sectional survey to investigate the effects of stress perceptions on depression, anxiety and stress symptoms. | May to June 2021 | - Literature-based individual items to assess demographic characteristics, work environment factors, and COVID-19 related increase in work stress - 21-item short form of the Depression, Anxiety, and Stress Scales (DASS) - Beliefs About Stress Scale (BASS) | Sample size*: n = 199 nurses  Age:  18-29 years = 12%  30-39 years = 38%  40-49 years = 30%  50-59 years = 19%  ≥60 years = 2%  Gender: Male = 26%, Female = 74% *Subgroup of a cross-setting study | Work-related stress | - 26% of nurses described an increased workload due to the current situation (Covid-19) as "rather true", 22% as "fully applicable" |
|  |  |  |  |  |  | Health | - 16% reported cardiovascular disease (vs. 6% of physicians). - 20% were overweight (vs. 5.5% of doctors). - 3% stated that they had a diagnosed mental illness   (It is unclear, however, how these results were collected)   - Nurses showed higher depressive symptoms with a mean score of 8.49 (SD = 8.41) than physicians, (M = 6.37 (SD = 7.36) - Anxiety symptoms were also more pronounced among nurses, with a mean score of 6.33 (SD = 6.89) compared to 3.13 (SD = 4.81) for physicians - In terms of psychological distress, there was no significant difference between the two occupational groups. - Negative stress beliefs increased the negative effects of work stress on mental health |
| Walter N, Wimalan B, Baertl S, Lang S, Hinterberger T, Alt V, Rupp M (2022) [70] | Germany (not specifically defined region) | Qualitative interview study to investigate the effects of periprosthetic joint infections (PJI) on the well-being of nurses and to identify challenges. | March 2021 and June 2021 | Semi structured interviews | Sample size: N = 20 nurses  Age (min–max):  21–55 years  Gender: Male = 15%, Female = 85% | Work-related stress | - The psychological stress of patients as well as their lack of adherence to therapy due to insufficient perception of the severity of the disease are central challenges in dealing with PJI. - Collegial support and exchange in case discussions were perceived as effective coping strategies. - Involving psychologists in treatment, implementing measures to improve compliance, and providing professional counseling could be beneficial approaches to reducing stress |
| Özkaytan Y, Kukla H, Schulz-Nieswandt F, Zank S (2024) [71] | Germany (not specifically defined region) | Qualitative study using semi structured interviews to investigate the perception of health professionals in rural long-term care facilities in Germany with a focus on integrated health systems. | April 2022 to May 2023 | Semi structured interviews | Sample size: N = 20 nurses from rural long-term care facilities  Age (min–max):  23-55 years  Gender: Male = 30%, Female = 70% | Work-related stress | - Within the study sample, nursing staff in long-term care settings were frequently the first to respond in acute situations but reported delegating responsibility due to uncertainty or perceived hierarchical constraints - Some participants expressed concerns regarding potential legal repercussions and reported feelings of insecurity or inferiority when working in interdisciplinary teams, particularly with emergency medical personnel - Night shifts were described as particularly challenging, with participants indicating feelings of being overwhelmed and insufficiently empowered to act independently due to limited competencies or authority - Incomplete or unavailable patient information was perceived as a barrier to adequate care and was associated with potentially avoidable hospital transfers - Participants noted that flat hierarchical structures facilitated interprofessional collaboration, whereas rigid organizational hierarchies were perceived as obstructive - The data reflect a perceived need for increased interdisciplinary training opportunities and the promotion of a culture of mutual respect among professional groups - Additionally, some participants articulated a desire for sustained political and media support for the nursing profession, particularly in light of the temporary recognition received during the COVID-19 pandemic |
| Petersen J, Melzer M (2023) [72] | Germany (nationwide) | Cross-sectional study conducted as an anonymous online survey to investigate predictors and consequences of moral distress in home care. | May 2022 to June 2022 | - Moral Distress Scale - COPSOQ III | Sample size: N = 976 outpatient nurses  Age: Mean = 46.8 years (SD = 10.5)  Gender: Male = 18%, Female = 82% | Work-related stress | Morally stressful situations:   - 46% of nurses experienced high or very high distress when they were unable to prevent a patient's suffering due to inappropriate medical orders - 36% felt heavily burdened when they had to work with unqualified colleagues, which jeopardized the quality of care - 34% felt that staffing was inadequate and morally burdensome   Predictors of moral distress:   - High emotional demands, frequent work-life conflicts, low influence in the workplace and low social support are associated with moral distress - Organizational factors, such as limited time with patients, contribute to the moral burden - Higher scores in the degree of disorder caused by MD meant significantly higher chances of indicating the intention to leave the profession (OR = 1.48) |
|  |  |  |  |  |  | Health | - High moral stress increases the risk of burnout, with an increase of one unit increasing the burnout score by 0.251 points. - Health deteriorated with increasing moral strain, with health status falling by 0.450 points. - No direct correlation between moral stress and absenteeism due to illness could be proven, but presenteeism could play a role |
| Studies investigating the effects of the COVID-19 pandemic (survey period: during the pandemic) | | | | | | | |
| Studies attributable to the first COVID-19 wave (March to May 2020) and the summer plateau (May to September 2020) [61] | | | | | | | |
| Hower KI, Pfaff H, Pförtner T-K (2020) [73] | Germany (nationwide) | Cross-sectional study conducted as an anonymous online survey of managers to record pandemic-related challenges in nursing. | April 2020 | Literature-based individual items to assess demographic characteristics, work environment factors, and COVID-19 related increase in work stress | Sample size: N = 525 nursing managers from outpatient and inpatient care and palliative care facilities  Age:  26-35 years = 9%  36-45 years = 26%  46-55 years = 38%  56-65 years = 26%  >65 years = 1%  Gender: Male = 66%, Female = 33%, non-binary = 1%) | Work-related stress | - Leaders are facing a variety of pandemic-related challenges - The biggest challenges include concerns about infection among people in need of care (70% rated as highly to very highly stressful) and employees (71% rated as highly to very highly stressful), as well as the procurement and consumption of equipment to protect against infection (68% rated as highly to very highly stressful) - Work intensification and states of overload already existed before the pandemic and are generally disproportionate to the perceived lack of social and financial recognition - The COVID19 pandemic has led to an additional increase in challenges and burdens |
|  |  |  |  |  |  | Health | - Increased incidence of presenteeism (both among managers and nursing staff in general - Managers assessed their state of health worse at the present time during the pandemic than in retrospect before the outbreak of the pandemic. - The respondents stated that they came to work more often during the pandemic than before the outbreak of the pandemic despite existing illness or a feeling of illness (increase in presenteeism) |
| Eggert S, Teubner C (2021) [74] | Germany (nationwide) | Nationwide cross-sectional survey conducted as computer-assisted telephone interviews (CATI) to investigate the extent to which nursing staff were confronted with additional requirements in the context of the Corona pandemic. | August to September 2020 and July 2020 | Details not specified | Sample size:  N = 1000 nursing service managers, quality officers or managing directors **of outpatient services**  N = 950 nursing service managers, quality officers or managing directors **of inpatient care facilities**  Age and gender were not reported | Work-related stress | - In the wake of the pandemic, 40% of outpatient services report an increase in physical stress - In the wake of the pandemic, 58% of outpatient services report an increase in psychological stress - In the course of the pandemic, 39% of inpatient nursing homes report an increase in physical stress - In the course of the pandemic, 65% of inpatient nursing homes report an increase in psychological stress - Total increased mental and physical stress during the pandemic |
| Bartzik M, Aust F, Peifer C (2021) [75] | Germany (nationwide) | Cross-sectional study conducted as an anonymous online survey to investigate buffer factors in dealing with pandemic-related stress to protect the mental health of nursing staff. | June to July 2020 | - Literature-based individual items to assess demographic characteristics, work environment factors, and COVID-19 related increase in work stress - Subscale for emotional exhaustion of the German version of the Maslach Burnout Inventory (MBI-D) - Flow frequency scale by Bartzik and Peifer - Sense of Humor Scale (SHS-P) | Sample size: N = 174 registered nurses  Age: Mean = 40.5 (SD = 10.8)  Gender: Male = 7%, Female = 93% | Work-related stress | - 66% reported an increase in workload (18% unchanged, 16% decrease) - Participants were most concerned about the health of their families and friends, less about their own health and least concerned about their economic future - During the pandemic, nurses experienced more appreciation from society, but less from their patients - During the COVID-19 pandemic, nurses felt less flow (satisfaction with work, life, work performance, well-being) than before |
|  |  |  |  |  |  | Health | - The COVID-19 pandemic affected the stress levels of nurses - During the COVID-19 pandemic, nurses experienced more stress, had higher scores of emotional irritation, and felt more emotionally exhausted than in the pre-COVID-19 era.   Coherences:   - Humor overall was associated with lower exhaustion and more frequent flow experiences - Certain facets of humor such as enjoyment of humor, humor in everyday life and humor under stress were also associated with higher job and performance satisfaction as well as less emotional irritation during the pandemic - Appreciation by patients was accompanied by less exhaustion, more flow and higher job satisfaction - Appreciation by society was particularly associated with more frequent flow experiences |
| Morawa E, Schug C, Geiser F, Beschoner P, Jerg-Bretzke L, Albus C, Weidner K et al. (2021) [76] | Germany (nationwide) | Cross-sectional study conducted as an anonymous online survey to investigate psychological stress and working conditions during the COVID-19 pandemic in Germany. | April to July 2020 | - PHQ4 - Literature-based individual items to assess demographic characteristics, work environment factors, and COVID-19-related factors | Sample size*: n = 1275 nurses  Age:  18-30 years = 29%  31-40 years = 25%  41-50 years = 21%  51-60 years = 22%  >61 years = 4%  Gender: Male = 25%, Female = 75%  *Subgroup of a cross-setting study | Work-related stress | - There was no specific evaluation of the stress factors for the nursing staff - Nurses reported sufficient staffing less often than doctors and medical technicians |
|  |  |  |  |  |  | Health/ Health behavior | - The prevalence of clinically significant depressive symptoms for nurses was 23% - The prevalence of clinically significant anxiety symptoms for nurses was 20% - Compared to the German general population, nurses had lower levels of depression/anxiety during the COVID-19 pandemic - Multiple linear regression analyses showed that higher levels of depressive symptoms were associated with insufficient recovery in leisure time, increased alcohol consumption, and lower trust in colleagues in difficult work situations - Increased anxiety scores were associated with an increased fear of COVID-19 infection |
| Kirmse KA, Pietrzyk U, Hacker W, Saifoulline R, Fuchs K, Haubold A-K (2021) [77] | Germany (nationwide) | Cross-sectional study conducted as an anonymous online survey to record pandemic-related challenges in nursing. | August 2020 | - Self-developed screening instrument based on DIN EN ISO 10075-1 - Mohr's Irritation Scale - MBI-D - Short-Form-Health Survey (SF-12) | Sample size: N = 451 nurses  Age and gender were not reported | Work-related stress | - Overall high levels of workload and strain (The mean value for work overload was 3.58 [SD = 0.69] on a 5-point Likert scale) - In particular, work intensity represents a central problem area, reflected in above-average values for working under time pressure, multitasking, and interrupted breaks - Pronounced strain-related consequences were also found in connection with emotional dissonance |
|  |  |  |  |  |  | Health | - Associated health impairments were mainly reflected in the extent of emotional exhaustion (MBI-D) and mental health (SF-12) and point to the urgency of reducing stress in nursing |
| Skoda E-M, Teufel M, Stang A, Jöckel K-H, Junne F, Weismüller B, Hetkamp M et al. (2020) [78] | Germany (nationwide) | Cross-sectional study conducted as an anonymous online survey to compare the psychological stress situation of health professionals (doctors, nurses, rescue workers) vs. non-health professionals (nHP). | March 2020 | - Pandemic-related   Worries/Anxiety   - Generalized Anxiety Disorder Scale (GAD-7) - PHQ-2 - EuroQoL/EQ-5D-3L - Literature-based individual items to assess demographic characteristics, work environment factors, and COVID-19-related factors | Sample size:  n = 1511 nursing staff  Age: not reported  Gender nursing staff: Male = 13%, Female = 87%  n = 492 physicians  n = 221 paramedics  n = 639 nHPs | Health | - Health professionals (Physicians, nursing staff, paramedics) show less generalized anxiety, depression, COVID-19-related anxiety than the nHPs - Physicians exhibit lower levels of generalized anxiety compared to both nursing staff and nHPs. The differences are of moderate effect size (physicians vs. nursing staff: d = −0.213, CI = [−0.315 to −0.111]; physicians vs. nHPs: d = −0.342, CI = [−0.432 to −0.251]). - Among all healthcare professionals, nursing staff report the highest levels of generalized anxiety - Differences in depression levels are also evident between the various health professional (HP) groups as well as between HPs and non-health professionals (nHPs) (η² = 0.009, CI = [0.006–0.012]). - Physicians show lower depression scores than nursing staff and nHPs (physicians vs. nursing staff: d = −0.21, CI = [−0.312 to −0.108]; physicians vs. nHPs: d = −0.377, CI = [−0.468 to −0.287]). Nursing staff show lower depression scores than nHPs (nursing staff vs. nHPs: d = 0.167, CI = [0.113–0.221]) - Nurses exhibit poorer health than physicians and paramedics; however, their health status is only slightly better rated than that of non-health professionals (d = −0.115, CI = [−0.169 to −0.061]) - HPs show less COVID-19-related anxiety than nHPs (all ds > 0.2 with confidence intervals excluding 0). Physicians and nursing staff exhibit similar levels (η² = 0.01, CI = [0.008–0.015]; d = −0.003, CI = [−0.105 to 0.10]) - Overall, participants reported a high subjective level of information regarding COVID-19, with mean values above 5.5 in all groups (HPs and nHPs) - A high subjective level of information regarding COVID-19 was overall negatively correlated with a high level of generalized anxiety symptoms |
| Zerbini G, Ebigbo A, Reicherts P, Kunz M, Messman H (2020) [79] | Germany (Augsburg) | Cross-sectional study to record psychosocial stress of employees in the health care system during the COVID-19 pandemic. | March and April 2020 | - PHQ - MBI (Burnout) - Literature-based individual items to assess demographic characteristics, and COVID-19-related factors/ | Sample size*: n = 75 nurses at Augsburg University Hospital  Age and gender were not reported for the subgroup  *Subgroup of a cross-setting study | Work-related stress | Stress factors:   - 38% cited the workload in general - 30% cited uncertainty - 24% care for people themselves - 16% reported psychosocial stress - 13% saw the risk of infection as a burden   Key Resources:   - 64% named psychosocial support from family and friends - 45% named time off - 23% named psychosocial support in the workplace - 13% named personal resilience factors - 6% benannten Religion   Desired improvement measures:   - The most common suggestion for improvement related to better adaptation of the infrastructure to COVID-19 in the hospital: 51% of the participants wanted more staff and/or rooms, better organization and planning (e.g. staff scheduling, standardized approach to COVID-19 patients) and the maintenance of stable teamwork. - 20% wanted better communication - 20% wanted higher pay - 14% would like to see more compensatory time off - 20% see sufficient protective equipment as potential for improvement - 20% named better psychosocial support as an improvement measure |
|  |  |  |  |  |  | Health | - Fear of COVID-19 infection correlated positively with the MBI fatigue scale and with the three PH-Q subscales (depression; Fear; Stress) - Likewise, participants who stated that they currently felt more stressed at work showed increased burnout symptoms and psychological stress. - Nurses in the COVID-19 wards reported more often of depressed mood, exhaustion and lower fulfillment than nurses in the normal wards |
| Studies attributable to the second COVID-19 wave (September 2020 to February 2021) [61] | | | | | | | |
| Hower KI, Pfaff H, Pförtner TK (2021) [80] | Germany (nationwide) | Repeated cross-sectional survey of Hower et al. [73] conducted as an anonymous online survey of managers to record the changes in pandemic-related challenges in nursing. | December 2020 to January 2021 | Literature-based individual items to assess demographic characteristics, and COVID-19-related factors/ COVID-19 related increase in work stress | Sample size: N = 294 nursing managers from outpatient and inpatient care and palliative care facilities  Age and gender were not reported for the subgroup | Work-related stress | - The burdens have increased over the course of the pandemic, especially in outpatient facilities, as well as with regard to general demands on staff (e.g. staff shortages and overload) and work organization (e.g. compliance with working time regulations or staffing ratios) - The concern about infections of people in need of care and employees remains the greatest burden in the course of the pandemic |
| Schaps V, Hower KI, Pfaff H, Pförtner T-K (2024) [81] | Germany (nationwide) | Cross-sectional study conducted to investigate irritations, general and pandemic-related requirements and the importance of workplace health promotion in long-term care. | December 2020 to January 2021 | - Irritation Scale by Mohr - Literature-based individual items to assess demographic characteristics, availability of workplace health promotion, and COVID-19-related factors/ COVID-19 related increase in work stress | Sample size: N = 207 nursing staff with management position  Age and gender were not reported | Work-related stress /Health | Relationship between stress and irritation:   - There is a significant positive association between general and pandemic-related stress as well as irritation (psychological stress in the form of cognitive and emotional tension, e.g. persistent brooding, irritability or emotional exhaustion) among nursing staff in management functions - Higher loads lead to higher irritation   Stress factors in inpatient and outpatient care:   - The pandemic-related burdens (including the procurement of infection protection material, isolation of infected persons in need of care, compliance with contact restrictions for relatives) were higher in inpatient care facilities (67%) than in outpatient services (61%). - General stress (including high workload, emotional stress due to illness and death, lack of staff) was also higher in inpatient facilities (62% vs. 56%). - On average, irritation was slightly higher among managers in outpatient care (46% vs. 44%).   Influence of workplace health promotion (WHP)   - WHP was more frequently implemented in inpatient care facilities (90% vs. 62% in outpatient services) - Facilities with WHP services showed significantly lower average irritation values (43% with WHP vs. 53% without WHP). - However, WHP could not significantly moderate the association between stress and irritation |
| Mai T, Todisco L, Schilder M, Franke V, Ristau J (2022) [82] | Germany (Hesse) | Cross-sectional study conducted as an anonymous online survey to record the situation of nurses in acute care hospitals in Hesse during the second wave of the COVID-19 pandemic. | December 2020 to January 2021 | - Literature-based items to assess sociodemographic characteristics, physical and psychosocial stressors, as well as perceived support measures from employers and policymakers - BAT - Work-SOC (work-related – Sence of Coherence) - perceived organizational Support – short version (POSs) | Sample size*: n = 595 nurses working at N = 81 hospitals in Hesse  Age: <35 years = 39.5%, ≥35 years = 60.5%  Gender: Male = 24%, Female = 76%  *Subgroup of a cross-setting study | Health | - 48% of respondents were at moderate or high risk of burnout - Nurses working in COVID or transitional (gray) areas had significantly higher burnout risk than those in non-COVID areas - There was a significant correlation between burnout and the intention to leave the job - The higher the sense of coherence and perceived organizational support, the lower the burnout risk - Factors positively influencing Work-SoC included access to training, older age, and a feeling of contributing to the pandemic response - Factors negatively influencing Work-SoC included lack of employer support, inability to provide adequate patient care, and declining personal engagement - 21% reported wanting to change jobs after the pandemic - 55% disagreed that their employer was doing everything possible to support them - Only 25% reported having access to training opportunities |
| Bauer J, Kocks A, Luboeinski J, Fischer U (2021) [83] | Germany (nationwide) | Cross-sectional study conducted as an anonymous online survey  of the Network for Nursing Science and Practice Development in the Association of Nursing Directors of the University Hospitals and Medical Schools of Germany (VPU) in cooperation with the Federal Association of Nursing Management e.V. | Oktober 2020 to November 2020 | Literature-based individual items to assess demographic characteristics, and COVID-19-related factors/COVID-19 related increase in work stress | Sample size: N = 128 nursing managers  Age and gender were not reported | Work-related stress | Changed job profile   - Support of the transformation process by nursing managers - Increased need for communication at all levels - Active participation in control and decision-making processes   General concerns of care managers during the Corona pandemic   - 81% were worried about Covid-19 infection among employees - 87% were worried about physical and mental overload of employees - 73% reported high work intensity and intensification among employees - 85% were worried about sufficient (protective) material   Personnel:   - 44% reported an increase in the number of staff in the nursing service due to the corona pandemic   Measures to protect the health of employees   - 95% reported that additional hygiene training was carried out - To minimize psychosocial risks, 56% of respondents said psychosocial support was provided to employees. - 25% reported the use of case discussions or supervision - 14% reported on the offer of health coaching |
| Dürr L, Forster A, Bartsch CE, Koob C (2022) [84] | Germany (nationwide) | Cross-sectional study conducted as an anonymous online survey to investigate the requirements, resources and work commitment of nurses during the second wave of the COVID-19 pandemic. | November to Dezember 2020 | - COPSOQ (abridged) - Literature-based individual items to assess demographic characteristics, and COVID-19-related factors - Utrecht Work Engagement (UWES-9) - Global Transformational Leadership Scale (GTL) | Sample size: N = 1027 nurses  Age:  < 20 years = 2%  20 – 25 years = 29%  26 – 35 years = 37%  36 – 55 years = 26%  > 55 years = 4%  Gender: Male = 17%, Female = 83% | Work-related stress /Health | - The overload of work was perceived by nurses as the strongest demand (M = 3.58, SD = 0.69), significantly higher than work-life interference (M = 2.96, SD = 0.83) and lack of formal reward (M = 2.88, SD = 0.78) - Workloads were perceived as even more intense than before the COVID-19 pandemic, with significantly higher values compared to pre-pandemic reference data (M = 3.58, SD = 0.69 vs. M = 3.48, SD = 0.64; t = 4.39, p < 0.001), while work-life conflicts were less pronounced (M = 2.96, SD = 0.83 vs. M = 3.22, SD = 1.12; t = –8.48, p < 0.001) - The basic structure of resources remained unchanged, but interpersonal relationships (M = 4.12, SD = 0.60) and autonomy (M = 2.89, SD = 0.80) were perceived more positively than before the pandemic (compared to pre-pandemic values: M = 3.79 and M = 2.62 respectively; both p < 0.001) - Nurses in outpatient care rated both job demands and resources more favorably than those in hospitals or nursing homes (e.g., autonomy: M = 3.38 vs. 2.62; p < 0.001). - The care of COVID-19 patients led to a more critical assessment of job conditions: significantly higher work overload (M = 3.75 vs. 3.41; p < 0.001), greater work-life interference (M = 3.10 vs. 2.82; p < 0.001), and a stronger sense of lack of formal rewards (M = 2.96 vs. 2.79; p < 0.01) - During the second wave, work engagement was higher than during the first (M = 4.41, SD = 1.12 vs. M = 4.28, SD = 1.20; t = 2.26, p < 0.05), but still below the reference value for a “high” level (cutoff 5.20; t = –20.78, p < 0.001) |
| Hering C, Gangnus A, Budnick A, Kohl R, Steinhagen-Thiessen E, Kuhlmey A, Gellert P (2022) [85] | Germany (nationwide) | Cross-sectional study conducted as an anonymous online survey to investigate psychosocial stress and related factors among nursing home nurses during the COVID-19 pandemic. | November 2020 to February 2021 | - CoPaQ - SDASS-21 - GAD-2 - PHQ-2 - COPSOQ - Literature-based individual items to assess demographic characteristics, and COVID-19-related factors/COVID-19 related increase in work stress | Sample size: N = 811 nursing home nurses  Age: Mean = 39.5 years (SD = 10.7)  Gender: Male = 9%, Female = 91%, | Work-related stress | - 94% of nurses surveyed said their work demands have increased since the start of the pandemic - 42% showed dissatisfaction with the COVID-19 management of the nursing home manager - A multiple regression analysis showed significant associations between COVID-19-related stress and qualification, dissatisfaction with the COVID-19 management of the home management, COVID-19-related anxiety and dementia as a care focus |
|  |  |  |  |  |  | Health | - 39% of nurses showed moderate to extremely severe stress symptoms according to the Stress Scale (with the occurrence of COVID-19 cases among residents associated with increased stress among nurses) - 37% and 41% of nurses showed clinically relevant symptoms of anxiety and depression, respectively - Overall, 59% of nurses had a positive impact in at least one of the three screening instruments used (SDASS-21, GAD-2, PHQ-2) - Stress, depression and anxiety showed associations with COVID-19-related stress at work, COVID-19-related anxiety, social support and a sense of community - Stress was also associated with the number of COVID-19 cases among nursing home residents and the size of the nursing home |
| Studies attributable to the third COVID-19 wave (March to June 2021) or a later COVID-19 wave [61] | | | | | | | |
| Lücker P, Henning E, Kästner A, Hoffmann W (2024) [86] | Germany (nationwide) | Qualitative interview study conducted using semi structured focus group interviews to investigate the willingness of inactive nurses to return to active care during the COVID-19 pandemic. | October to November 2021 | Semi structured focus group interviews | Sample size: N = 18 nurses  Age and gender were not reported | Work-related stress | - Inactive nurses often have negative memories of their previous work, especially heavy workloads, understaffing, and low appreciation, which reduces their willingness to return - The fear of patient safety risks, lack of knowledge and being overwhelmed increases uncertainties and can lead to the rejection of a return - The legal and organizational complexity of a temporary return makes it difficult to return to work and means that many inactive nurses cannot be reactivated - Many inactive nurses fear that their skills are no longer sufficient and that they will no longer be able to move safely in everyday care - Especially in times of pandemic, the fear of infection and a lack of protective equipment is a central issue - Nurses experience ethical dilemmas, especially when they can't provide the care they feel is necessary. This can have long-term psychological consequences and make a return unlikely - The lack of social and financial respect for the nursing profession is perceived as frustrating and contributes to the decision not to return   Measures to promote the willingness to return:   - Transparent crisis communication and clear expectations of returnees are crucial to reduce uncertainty - Regular training or online courses can increase confidence in one's own abilities and increase readiness for action - Flexible deployment models that allow a temporary return without jeopardizing the current job - A structured introduction as well as psychological support can help to reduce the burden and enable a successful return   Conclusion:   - The "hidden reserve" of nurses is limited: only a third of inactive nurses would be willing to return in a crisis, which is why strategic planning is necessary - Structural improvements are crucial - Unbureaucratic procedures, financial incentives and further training opportunities are essential to motivate inactive nurses to return in the event of a crisis |
| Kuhlmann E, Behrens GMN, Cossmann A, Homann S, Happle C, Dopfer-Jablonka A (2022) [87] | Germany (Hannover) | Cross-sectional survey conducted as an online survey to investigate perceived risks, protective and preventive measures in the pandemic. | November 2020 to February 2021 | Literature-based individual items on the risk of infection, fear of infection, actual infection, protection/prevention measures | Sample size*: n = 461 nurses  Age and gender were not reported for the subgroup  *Subgroup of a cross-setting study | Work-related stress | The nurses surveyed in this study showed a significantly higher personal risk perception and greater fear of infection compared to other occupational groups in the healthcare sector (p = 0.004) |
| Bußmann A, Pomorin N (2023) [88] | Germany (North-Rhine Westphalia) | Longitudinal cohort study to record changes in psychosocial stress in connection with the Corona pandemic. | Before the pandemic: Oktober to November 2019  During the pandemic: April to June 2022 | COPSOQ | Two independent samples  Sample size 1: N = 55 nursing staff from two palliative care nursing homes  Gender: Male = 18%, Female = 76%, non-binary/missing = 6%  Age:  Most participants were between 45 and 54 years old  Sample size 2: N = 58 nursing staff from two palliative care nursing homes  Gender: Male = 22%, Female = 76%. non-binary/missing = 2%  Age:  Most participants were between 45 and 54 years old | Work-related stress /Health | As part of this study, positive and negative changes in psychosocial stress were identified.   - The four categories "resolution", "role conflicts", "role clarity" and "burnout symptoms in connection with relatives" were perceived more strongly in 2022 than in 2019 - Positive changes from 2019 to 2022 were seen in the categories: "Quantitative requirements", "Influence in the workplace", "Job insecurity", "Uncertainty about working conditions", "Burnout symptoms in connection with residents", "Degrees of freedom (breaks/holidays)" and "Presenteeism" - Only the changes in the categories "degrees of freedom (breaks/holidays)" and "presenteeism" proved to be statistically significant in the t-test - Other studies show sometimes contradictory results, including with regard to presenteeism and emotional distress |
| Winnand P, Fait Y, Ooms M, Bock A, Heitzer M, Laurentius T, Bollheimer LC et al. (2023) [89] | Germany (not specifically defined region) | Cross-sectional study conducted as an anonymous paper-based survey to assess mental and physical stress factors among nurses in different functional areas before and during the COVID-19 pandemic. | December 2022 | - MBI - Literature-based individual items to assess demographic characteristics, and COVID-19-related factors | Sample size: N = 224 nurses of a maximum care hospital  Age and gender were only reported for subgroups | Work-related stress | - During the COVID-19 pandemic, ward and operating room nurses were exposed to a significantly increased workload and time pressure - Ward and operating room nurses experienced a deterioration in the working atmosphere with increased mutual recriminations - After considering factors such as age, gender, shift work, the care of COVID-19 patients and the impact of the pandemic on private life, the feeling of "working with pleasure" was significantly reduced among ward and OR nurses compared to OR nurses - In addition, ward and surgical nurses were significantly more likely to say they wanted to give up their profession than surgical nurses |
|  |  |  |  |  |  | Health | - Regardless of the field of activity, nurses showed an increased tendency to develop subclinical affective symptoms. - 24-41% of nurses showed increased feelings of frustration and burnout as a result of the COVID-19 pandemic |
| Cross-wave studies according to assignment according to Tolksdorf et al. [61] | | | | | | | |
| Schulze S, Merz S, Thier A, Tallarek M, König F, Uhlenbrock G, Nübling M et al. (2022) [90] | Germany (Brandenburg) | Mixed-methods study with qualitative and quantitative data collection and analysis methods to record the psychosocial burden of nursing home nurses during the Covid-19 pandemic. | August to October 2020; Interviews took place in June 2021 | - COPSOQ III - Semi-structured interviews | Cross-sectional study:  Sample size: N = 177 nurses from nursing homes  Age: Mean = 44.1 years (SD = 11.7)  Gender: Female = 83%  Interviews:  Sample size: N = 15 nurses from nursing homes  Age: not reported  Gender: Male = 20%, Female = 80% | Work-related stress /Health | - The survey sample deviated significantly from the pre-pandemic reference group (aggregated data from geriatric nurses collected in 2015–2019) in 14 out of 31 COPSOQ scales - Most of these discrepancies indicated a deterioration, especially in the scales "quantitative requirements", "emotion concealment", "work-private life conflicts", "role conflicts", "leadership quality", "support at work", "recognition", "physical stress", "job abandonment thoughts", "burnout", "presenteeism" and "inability to relax" - The interviews revealed six central topics related to psychosocial stress among nursing staff: "general working conditions", "concern for residents", "dealing with relatives", "difficult end-of-life care", "tension between self-infection and external infection" and "mechanization of care" - The positive effects of the pandemic were a "stronger community cohesion" in the interviews and an "increased sense of purpose" and an "increase in social contacts" in the COPSOQ III survey |
| Studies that cannot be attributed to a COVID-19 wave due to a lack of information on the survey period | | | | | | | |
| Krieger H, Rhein C, Morawa E, Adler W, Steffan J, Lang-Richter N, Struck M et al. (2024) [91] | Germany (Erlangen, Ebermannstadt) | Prospective observational study to investigate physical and mental stress as subjective stress parameters in nurses during the COVID-19 pandemic and heart rate variability (HRV) as an objective stress indicator. | no exact information | - Stress diary - HRV (24-hour measurement) by means of shirts with integrated ECG electrodes - PHQ-9 - Literature-based individual items to assess demographic characteristics, and COVID-19-related factors | Sample size: N = 41 nurses from hospitals  Age: 18–63 years  Gender: Female = 61% | Work-related stress | The pandemic stressor:   - 10% of the participants were afraid of infection - 61% of the participants reported an above-average occupancy rate of the stations - 31% were afraid of infecting relatives |
|  |  |  |  |  |  | Health | - 81% of nurses were unable to recover sufficiently in their free time, but this was not significantly related to HRV - 37% of participants showed clinically significant depressive symptoms - 32% of participants showed clinically significant anxiety - When comparing working days with a day off, a significant difference was observed in terms of physical load, mental load, emotional exhaustion, and overload with higher values on working days - In summary, the analysis revealed a discrepancy between subjective and objective stress levels. The participants felt subjectively recovered on a day off, but biologically, measured by HRV, there was no difference between work and leisure - Further studies are needed to evaluate and differentiate the influence of work stress and other types of stress on heart rate variability |
| Wildgruber D, Frey J, Seer M, Pinther K, Koob C, Reuschenbach B (2020) [92] | Germany (nationwide) | Cross-sectional study conducted as an anonymous online survey to record pandemic-related challenges in nursing. | No information on the survey period | - Literature-based individual items to assess demographic characteristics, and COVID-19-related factors - UWES | Sample size*: n = 855 nursing staff  Age and gender were not reported for the subgroup  *Subgroup of a cross-setting study | Work-related stress | - 54% of nursing staff reported that they feel stressed in their everyday work due to the current Corona pandemic - Direct contact with COVID-19 patients increases stress (concern about the risk of infection) - 39% were concerned about their own well-being (but worries about the well-being of others were at the forefront) - A multiple regression analysis showed that the - Work engagement decreases with increasing pandemic-related stress |
| Reviews in the context of the Corona pandemic | | | | | | | |
| Benzinger P, Kuru S, Keilhauer A, Hoch J, Prestel P, Bauer JM, Wahl HW (2021) [93] | Germany (nationwide) | Systematic review to investigate the psychosocial impact of the pandemic on nursing home staff and residents and their relatives. | January 2020 to November 2020 | Study selection was carried out in the same way as the "Preferred Reporting Items for Systematic Reviews and Meta-Analyses (PRISMA) statement" for systematic reviews | 1 concept-relevant study [94] with sample size: n = 20 nurses | Work-related stress | - Employees report an increased workload in the wake of the pandemic - In addition, in contrast to other surveys, the respondents reported improved cohesion within the teams, fewer absences due to illness and a quieter working atmosphere |
| Arndt D, Hering T (2025) [95] | Germany (nationwide) | Scoping Review to Record Workload, Resources, Stress Consequences in the COVID-19 Pandemic for Nursing Professionals. | June 2023 to February 2024 | JBI-Methodologie | n = 50 studies  (Of these, n = 36 studies are based on quantitative designs, in particular online surveys with a sample size of 55 to 2689 participants; n = 14 studies were qualitative interview studies with a sample size of 8 to 510 participants)  Of these 50 studies, n = 12 studies are included as individual studies in the present overview [73, 75, 76, 78, 79, 82, 84, 85, 87, 88, 90, 92]  One additional study [96] was identified in the scoping review, but it was not explicitly included again as an individual study, since all relevant findings were already covered by the statements of the scoping review. | Work-related stress | - The most common stress factors in the wake of the pandemic include workload, hygiene and information requirements, qualification requirements and emotional demands - Social stress results from communication and relationships between professionals and patients or relatives, within teams and between employees and managers - Team aspects were mostly considered as a resource (e.g. support, cohesion) - The qualitative data point to close links between the stress factors - Cross-sectional studies show that the workload has increased since the beginning of the pandemic, with an increasing increase as the pandemic progresses - In the first phase of the pandemic and retrospectively up to April 2020, shortages of personal protective equipment (PPE) and disinfectants as well as increased labor intensity due to procurement effort were reported, with PPE shortages in hospitals being lower than in outpatient or inpatient long-term care. - Emotional demands included pandemic-related worries and fears, the confrontation with the death and suffering of patients, stigmatization, and moral burdens - The psychological stress of nursing staff was usually higher than that of doctors - at the beginning of 2020, anxiety and depression levels in the nursing industry were almost twice as high - At the end of 2020, 20% of nurses were often thinking about changing careers - In mixed samples, 38% expressed thoughts of quitting or changing jobs at least monthly, while this proportion was 18.9% in hospital samples |
|  |  |  |  |  |  | Health | - 40% of nurses reported high stress levels in the first year of the pandemic - In spring 2021, 60% of respondents reported high scores for moral stress - Conspicuous anxiety scores were found in 11% to 37% of nurses, and conspicuous depression scores were found in 14% to 42% - Nurses in mixed samples as well as in inpatient long-term care had higher anxiety and depression scores than hospital staff - A medium to high risk of burnout was found in 40–56% of respondents - Employees in COVID-19 areas were more stressed compared to those in non-COVID areas   Coherences:   - Pandemic-related anxiety was associated with more stress, burnout, anxiety and depression, as well as a higher risk of more than 10 sick days - Contact with COVID-19 patients was associated with more moral stress, less engagement, and higher burnout scores - The workload was related to more anxiety, a higher chance of conspicuous depression scores, and moral stress - The more moral stress was reported, the higher the levels of anxiety, depression, and burnout - Higher overall stress levels during the pandemic were associated with less engagement at work - Intentions to change/quit were reported above all by part-time nurses, who felt more stressed by pandemic-related and general stress factors and had higher burnout and depression scores - Depression and the feeling of being a burden to others were associated with suicidal thoughts |
| Abbreviations: AVEM = Work-Related Behavior and Experience Pattern, BASS = Beliefs About Stress Scale, BAT = Burnout Assessment Tool, BCTQ = Symptom scale of the Boston Carpal Tunnel Questionnaire, BHD = Screening Tool for Occupational Stress in the Social Sector, BORG-CR10 = Borg CR scale, CBI = Copenhagen Burnout Inventory, CG = Control Group, CoPaQ = COVID-19 Pandemic Mental Health Questionaire, COPSOQ = Copenhagen Psychosocial Questionnaire, DASS = Depression, anxiety and stress scales, ERSQ-27 = Emotion Regulation Skills Questionnaire, FBAS1 = Questionnaire on the Stress of Doctors and Nurses, GAD = Generalized Anxiety Disorder Scale, GPC = general palliative care, GTL = Global Transformational Leadership Scale, HRV = heart rate variability, ICU = intensive care unit, IG = intervention group, MBI-D = German Version of the Maslach Burnout Inventory, MDT = Moral Distress Thermometer, NCS = Nurse Competence Scale, NGNs = newly graduated nurses, NMQ = Nordic Musculoskeletal Questionnaire, NWI-PES = Practice Environment Scale of the Nursing Work Index, OCM = Organizational Climate Measure, OLBI = Oldenburg Burnout Inventar; PSQ= Perceived Stress Questionnaire; PHQ = Patients-Health-Questionnaire, POS-s = perceived organizational Support – short version,, PPE = personal protective equipment, REQ = Subscales of the Recovery Experience Questionnaire, RKI = Robert Koch Institute, RS-13 = Resilience Scale Questionnaire, SCL-90-R = Symptom-Checklist, SD = standard deviation, SDASS = Stress Scale DASS, SF-12 = Short Form Health Survey (12 Items), SHS-P = Sense of Humor Scale, SOAS-R = Staff Observation Aggression Scale-Revised, SOC = Sence-of-Coherence-Scale, SPC = specialised palliative care, SSCS (TICS) = 12-Item Screening Scale for Chronic Stress of the Trier Inventory for Chronic Stress, TAA-A = Task and Job Analysis Tool – Residential LTC Version, TAA-KH-S = Task and Job Analysis Tool – Hospitals-Version, TEQ = Toronto Empathy Questionnaire, UWES-9 = Utrecht Work Engagement Scale (9 Items), WAI = Work-Ability-Index, WHP = Workplace health promotion, Work-SOC = work related - Sense-of-Coherence-Scale | | | | | | | |

**References**

1. Körber M, Schmid K, Drexler H, Kiesel J. Subjective Workload, Job Satisfaction, and Work-Life-Balance of Physicians and Nurses in a Municipal Hospital in a Rural Area Compared to an Urban University Hospital. Gesundheitswesen. 2018;80(5):444-52. http://dx.doi.org/10.1055/s-0042-121596.

2. Graeb F. Ergebnisse. Ergebnisse. In: Ethische Konflikte und Moral Distress auf Intensivstationen: Eine quantitative Befragung von Pflegekräften. Best of Pflege. Springer, Wiesbaden. 2019. https://doi.org/10.1007/978-3-658-23597-0_5.

3. Breinbauer M. Arbeitsbedingungen und Arbeitsbelastungen in der Pflege: Eine empirische Untersuchung in Rheinland-Pfalz. 2020. https://dx.doi.org/10.1007/978-3-658-32021-8.

4. Ehegartner V, Kirschneck M, Frisch D, Schuh A, Kus S. Arbeitsfähigkeit von Pflegekräften in Deutschland – welchen Präventionsbedarf hat das Pflegepersonal: Ergebnisse einer Expertenbefragung. Das Gesundheitswesen. 2020;82(05):422-30. https://dx.doi.org/10.1055/a-0905-3007.

5. Diehl E, Rieger S, Letzel S, Schablon A, Nienhaus A, Escobar Pinzon LC, et al. Arbeitsbedingungen von Pflegekräften in der allgemeinen Palliativversorgung in Deutschland: Eine Querschnittbefragung. Pflege. 2021;34(2):80-91. https://doi.org/10.1024/1012-5302/a000791.

6. Diehl E, Rieger S, Letzel S, Schablon A, Nienhaus A, Escobar Pinzon LC, et al. Burdens, resources, health and wellbeing of nurses working in general and specialised palliative care in Germany – results of a nationwide cross-sectional survey study. BMC Nurs. 2021;20(1):1-16. https://doi.org/10.1186/s12912-021-00687-z.

7. Diehl E, Rieger S, Letzel S, Schablon A, Nienhaus A, Escobar Pinzon LC, et al. Health and intention to leave the profession of nursing - which individual, social and organisational resources buffer the impact of quantitative demands? A cross-sectional study. BMC Palliat Care. 2020;19(1):1-13. <https://doi.org/10.1186/s12904-020-00589-y>.

8. Schwarzkopf D, Rüddel H, Thomas-Rüddel DO, Felfe J, Poidinger B, Matthäus-Krämer CT, et al. Perceived Nonbeneficial Treatment of Patients, Burnout, and Intention to Leave the Job Among ICU Nurses and Junior and Senior Physicians. Crit Care Med. 2017;45(3):e265-e73. https://doi.org/10.1097/CCM.0000000000002081.

9. Werner NS, Bültmann M, Möckel L. Perceived stress, workload and psychosomatic complaints in inpatient and outpatient care nurses: A cross-sectional survey study. Pflege. 2023;36(4):220-7. <https://doi.org/10.1024/1012-5302/a000901>.

10. Hartog CS, Hoffmann F, Mikolajetz A, Schröder S, Michalsen A, Dey K, et al. Übertherapie und emotionale Erschöpfung in der „end-of-life care“ : Ergebnisse einer Mitarbeiterumfrage auf der Intensivstation. Anaesthesist. 2018;67(11):850-8. https://doi.org/10.1007/s00101-018-0485-7.

11. Knape C, Teubner A, Benkenstein A. Arbeitssituation mit einem rollierenden Arbeitszeitmodell in der ambulanten Pflege. HeilberufeSCIENCE. 2018;9(1/2):9-15. https://doi.org/10.1007/s16024-018-0312-8.

12. Isfort M. Evaluation of care conditions in intensive care units : Results of an online questionnaire of critical care nurses. Med Klin Intensivmed Notfmed. 2017;112(6):543-9. https://doi.org/10.1007/s00063-017-0292-y.

13. Mehlis K, Bierwirth E, Laryionava K, Mumm FHA, Hiddemann W, Heußner P, et al. High prevalence of moral distress reported by oncologists and oncology nurses in end-of-life decision making. Psycho-Oncology. 2018;27(12):2733-9. <https://doi.org/10.1002/pon.4868>.

14. Wagner A, Rieger MA, Manser T, Sturm H, Hardt J, Martus P, et al. Healthcare professionals' perspectives on working conditions, leadership, and safety climate: a cross-sectional study. BMC Health Serv Res. 2019;19(1):1-14. https://doi.org/10.1186/s12913-018-3862-7.

15. Lauxen O, Blattert B. Irritationen und Brüche in der beruflichen Identität internationaler Pflegefachpersonen: Eine qualitative Untersuchung. Pflegewissenschaft. 2021;23(2):75-82. <https://doi.org/10.3936/1864>.

16. Schilgen B, Handtke O, Nienhaus A, Mösko M. Work-related barriers and resources of migrant and autochthonous homecare nurses in Germany: A qualitative comparative study. Appl Nurs Res. 2019;46:57-66. https://doi.org/10.1016/j.apnr.2019.02.008.

17. Gencer D, Meffert C, Herschbach P, Hipp M, Becker G. Belastungen im Berufsalltag von Palliativpflegekräften – eine Befragung in Kooperation mit dem KompetenzZentrum Palliative Care Baden-Württemberg (KOMPACT). Gesundheitswesen. 2019;81(02):92-8. https://doi.org/10.1055/s-0043-109429.

18. Claaßen AC, Jeiler K, Martens D, Oetting-Roß C. Handlungsfelder und Arbeitsbereiche nach dem dualen Pflegestudium – Eine Verbleibstudie an der FH Münster. HeilberufeSCIENCE. 2021;12(1/2):30-8. https://doi.org/10.1007/s16024-021-00350-2.

19. Petersen J, Rösler U, Meyer G, Luderer C. Understanding moral distress in home-care nursing: An interview study. Nursing Ethics. 2024;31(8):1568-85. https://doi.org/10.1177/09697330241238338.

20. Ibenthal E, Hinricher N, Nienhaus A, Backhaus C. Hand and wrist complaints in dialysis nurses in Germany: a survey of prevalence, severity, and occupational associations. Ann Work Expo Health. 2024;68(2):136-45. https://doi.org/10.1093/annweh/wxad075.

21. Helaß M, Greinacher A, Genrich M, Müller A, Angerer P, Gündel H, et al. Nursing staff and supervisors perceptions on stress and resilience: a qualitative study. BMC Nurs. 2025;24(1):1-18. https://doi.org/10.1186/s12912-025-02712-x.

22. Raiber L, Kaluscha R, Tepohl L. Berufsbezogener Ü45-Gesundheitscheck: Liegt ein Bedarf bei Beschäftigten in der Pflege vor? Rehabilitation. 2024;63(6):349-56. https://doi.org/10.1055/a-2446-0262.

23. Baumann A-L, Kugler C. Berufsperspektiven von Absolventinnen und Absolventen grundständig qualifizierender Pflegestudiengänge - Ergebnisse einer bundesweiten Verbleibstudie. Pflege. 2019;32(1):7-16. <https://doi.org/10.1024/1012-5302/a000651>.

24. Seemann A-K, Fischer H. Was macht Freude im Arbeitsalltag, und was belastet? HeilberufeSCIENCE. 2017;8(3/4):136-41. <https://doi.org/10.1007/s16024-017-0308-9>.

25. Weigl M, Schmuck F, Heiden B, Angerer P, Müller A. Associations of understaffing and cardiovascular health of hospital care providers: A multi-source study. Int J Nurs Stud. 2019;99(null):N.PAG-N.PAG. https://doi.org/10.1016/j.ijnurstu.2019.103390.

26. Thomas Schramm TJ, Schröder H. Burnout am Arbeitsplatz bayerischer Pflegekräfte. Pflegewissenschaft. 2017;19(5/6):262-79. <https://doi.org/10.3936/1497>.

27. Korbus H, Hildebrand C, Schott N, Bischoff L, Otto AK, Jöllenbeck T, et al. Health status, resources, and job demands in geriatric nursing staff: A cross-sectional study on determinants and relationships. Int J Nurs Stud. 2023;145:104523. https://doi.org/10.1016/j.ijnurstu.2023.104523.

28. Weigl M, Schneider A. Associations of work characteristics, employee strain and self-perceived quality of care in Emergency Departments: A cross-sectional study. Int Emerg Nurs. 2017;30(null):20-4. <https://doi.org/10.1016/j.ienj.2016.07.002>.

29. Vaupel C, Vincent-Höper S, Helms L, Adler M, Schablon A. Sexuelle Belästing und Gewalt in Pflege-und Betreuungsberufen-Ergebnisbericht für die Pflegebranche-Bereiche stationäre Pflegeeinrichtungen und ambulante Pflegedienste. Hamburg: Berufsgenossenschaft für Gesundheitsdienst und Wohlfahrtspflege. 2021. https://www.bgw-online.de/resource/blob/22160/5d35353fe4c9037e6ae64a010e796808/bericht-gewalt-pflege-data.pdf. Accessed 22 Apr 2025.

30. Schablon A, Wendeler D, Kozak A, Nienhaus A, Steinke S. Prevalence and Consequences of Aggression and Violence towards Nursing and Care Staff in Germany-A Survey. Int J Environ Res Public Health. 2018;15(6). https://doi.org/10.3390/ijerph15061274.

31. Isfort M, Rottländer R, Weidner F, Gehlen D, Hylla J, Tucman D. Pflege-Thermometer 2018: Eine bundesweite Befragung von Leitungskräften zur Situation der Pflege und Patientenversorgung in der teil−-/vollstationären Pflege. 2018. <https://www.dip.de/fileadmin/data/pdf/projekte_DIP-Institut/Pflege_Thermometer_2018.pdf>. Accessed 22 Apr 2025.

32. Isfort M, Rottländer R, Weidner F, Tucman D, Gehlen D, Hylla J. Pflege-Thermometer 2016. Eine bundesweite Befragung von Leitungskräften zur Situation der Pflege und Patientenversorgung in der ambulanten Pflege. 2016. <https://www.dip.de/fileadmin/data/pdf/projekte_DIP-Institut/Endbericht_Pflege-Thermometer_2016-MI-2.pdf>. Accessed 22 Apr 2025.

33. Petersen J, Melzer M. Belastungs-und Beanspruchungssituation in der ambulanten Pflege. Bundesanstalt für Arbeitsschutz und Arbeitsmedizin (BAuA), Dortmund. 2022. https://doi.org/10.21934/baua:fokus20220516.

34. Rothgang H, Müller R, Preuß B. BARMER Pflegereport 2020. Belastungen der Pflegekräfte und ihre Folgen. 2020. <https://www.socium.uni-bremen.de/uploads/News/2020/20201201_BARMER_Pflegereport_2020.pdf>. Accessed 22 Apr 2025.

35. Techniker Krankenkasse. Gesundheitsreport. Pflegefall Pflegebranche? So geht’s Deutschlands Pflegekräften. 2019. <https://www.tk.de/resource/blob/2059766/2ee52f34b8d545eb81ef1f3d87278e0e/gesundheitsreport-2019-data.pdf>. Accessed 01. März 2025

36. Knieps F, Pfaff H. BKK Gesundheitsreport. Pflegefall Pflege. Berlin: Medizinisch Wissenschaftliche Verlagsgesellschaft. 2022. https://www.bkk-dachverband.de/fileadmin/user_upload/BKK_Gesundheitsreport_2022.pdf. Accessed 22 Apr 2025.

37. Vollbracht B, Gorgels S, Stuckert M. BeGX- Berufsgesundheits-Index Alten- und Krankenpflege – Branchenmonitoring der BGW und DRV Bund. 2023. <https://www.bgw-online.de/resource/blob/98106/d21ef62b077cd6607328248948c93495/bgw-datenbericht-begx-corona-pflegebranche-2023-data.pdf>. Accessed 22 Apr 2025.

38. Vollbracht M, Gorgels S. BeGX- Berufsgesundheits-Index Alten- und Krankenpflege – Branchenmonitoring der BGW und DRV Bund. 2024. <https://www.bgw-online.de/resource/blob/114162/e984ef4ded82f83881f6d90061998c3a/bgw-datenbericht-begx-pflegebranche-2024-data.pdf>. Accessed 22 Apr 2025.

39. Vollbracht M, Gorgels S, Hombücher V. BeGX- Berufsgesundheits-Index Alten- und Krankenpflege – Branchenmonitoring der BGW und DRV Bund. 2022. <https://www.bgw-online.de/resource/blob/76380/d6037d5569ee17e8a6c1bacbb17bcdf7/bgw55-83-113-datenbericht-begx-corona-pflegebranche-data.pdf>. Accessed 22 Apr 2025.

40. Hildebrandt-Heene S, Dehl T, Zich K, Nolting H-D. Gesundheitsreport. Analyse der Arbeitsunfähigkeiten: Gesundheitsrisiko Personalmangel: Arbeitswelt unter Druck. 2023. https://www.dak.de/dak/unternehmen/reporte-forschung/gesundheitsreport-2023_34592. Accessed 22 Apr 2025.

41. Dehl T, Hildebrandt-Heene S, Zich K, Nolting H-D. Gesundheitsreport. Analyse der Arbeitsunfähigkeiten: Gesundheitsrisiko Hitze: Arbeitswelt im Klimawandel. 2024. <https://www.dak.de/dak/unternehmen/reporte-forschung/gesundheitsreport-2024_66150#rtf-anchor-download-gesundheitsreport-2024-als-e-book>. Accessed 22 Apr 2025.

42. Klie T. DAK-PFLEGEREPORT. Die Baby-Boomer und die Zukunft der Pflege – Beruflich Pflegende im Fokus. Pflege. 2024. https://caas.content.dak.de/caas/v1/media/64750/data/42a02e597e07646cc80c0ddbd1382a8f/dak-pflegereport-2024-ebook.pdf. Accessed 22 Apr 2025.

43. Schaller A, Klas T, Gernert M, Steinbeißer K. Health problems and violence experiences of nurses working in acute care hospitals, long-term care facilities, and home-based long-term care in Germany: A systematic review. PLoS One. 2021;16(11):e0260050. https://doi.org/10.1371/journal.pone.0260050.

44. Frey D, Rieger S, Diehl E, Pinzon LCE. Einflussfaktoren auf chronische Rückenschmerzen bei Pflegekräften in der Altenpflege in Rheinland-Pfalz. Gesundheitswesen. 2018;80(2):172-5. https://doi.org/10.1055/s-0043-104693.

45. Bail C, Marquardt B, Harth V, Mache S. Technostresserleben in der stationären medizinischen Versorgung in deutschen und schweizerischen Kliniken: aktueller Forschungsstand. Zentralbl Arbeitsmed Arbeitsschutz Ergon. 2025;75(2):83-96. https://doi.org/10.1007/s40664-024-00542-3.

46. Kräft J, Wirth T, Harth V, Mache S. Digital stress perception among German hospital nurses and associations with health-oriented leadership, emotional exhaustion and work-privacy conflict: a cross-sectional study. BMC Nursing. 2024;23(1):213. https://doi.org/10.1186/s12912-024-01825-z.

47. Wirth LM, Ruppert N, Büscher A, Hülsken-Giesler M. Arbeitsschutz und Gesundheitsförderung im Kontext von Personalbemessung in der Pflege: Ein Scoping Review. Pflege. 2022;35(3):177-88. <https://doi.org/10.1024/1012-5302/a000873>.

48. Sturm H, Rieger MA, Martus P, Ueding E, Wagner A, Holderried M, et al. Do perceived working conditions and patient safety culture correlate with objective workload and patient outcomes: A cross-sectional explorative study from a German university hospital. PLoS One. 2019;14(1):e0209487. https://doi.org/10.1371/journal.pone.0209487.

49. Bernburg M, Groneberg D, Mache S. Professional training in mental health self-care for nurses starting work in hospital departments. Work. 2020;67(3):583-90. https://doi.org/10.3233/WOR-203311.

50. Jenner SC, Djermester P, Oertelt-Prigione S. Prevention Strategies for Sexual Harassment in Academic Medicine: A Qualitative Study. Journal of Interpersonal Violence. 2022;37(5/6):NP2490-NP515. <https://doi.org/10.1177/0886260520903130>.

51. Becker A, Angerer P, Weber J, Müller A. The prevention of musculoskeletal complaints: long-term effect of a work-related psychosocial coaching intervention compared to physiotherapy alone-a randomized controlled trial. Int Arch Occup Environ Health. 2020;93(7):877-89. https://doi.org/10.1007/s00420-020-01538-1.

52. Roth M, Altmann T. The interplay of acceptance and effectiveness in intervention studies: the example of the empCARE training to reduce burnout and distress symptoms in health care providers. Psychology and Health. 2024;39(8):1077-91. https://doi.org/10.1080/08870446.2022.2129053.

53. Berendonk C, Kaspar R, Bär M, Hoben M. Improving Quality of Work life for Care Providers by Fostering the Emotional well-being of Persons with Dementia: A Cluster-randomized Trial of a Nursing Intervention in German long-term Care Settings. Dementia (London). 2019;18(4):1286-309. https://doi.org/10.1177/1471301217698837.

54. Hoffmann A, Pilger S, Olbrecht T, Claassen K. Qualitative evaluation of a brief positive psychological online intervention for nursing staff. Arch Psychiatr Nurs. 2023;44:38-45. https://doi.org/10.1016/j.apnu.2023.04.003.

55. Ell J, Brückner HA, Johann AF, Steinmetz L, Güth LJ, Feige B, et al. Digital cognitive behavioural therapy for insomnia reduces insomnia in nurses suffering from shift work disorder: A randomised-controlled pilot trial. J Sleep Res. 2024;33(6):e14193. https://doi.org/10.1111/jsr.14193.

56. Roth C, Wensing M, Breckner A, Mahler C, Krug K, Berger S. Keeping nurses in nursing: a qualitative study of German nurses' perceptions of push and pull factors to leave or stay in the profession. BMC Nurs. 2022;21(1):1-11. https://doi.org/10.1186/s12912-022-00822-4.

57. Lützerath J, Bleier H, Gernert M, Schaller A. Implementing workplace health promotion in nursing – A process evaluation in different care settings. BMC Nurs. 2024;23(1):1-14. https://doi.org/10.1186/s12912-024-02272-6.

58. Riedl EM, Perzl J, Wimmer K, Surzykiewicz J, Thomas J. Short Mindfulness Meditations During Breaks and After Work in Everyday Nursing Care: A Simple Strategy for Promoting Daily Recovery, Mood, and Attention? Workplace Health Saf. 2024;72(11):491-502. https://doi.org/10.1177/21650799241262814.

59. Schaller A, Gernert M, Klas T, Lange M. Workplace health promotion interventions for nurses in Germany: a systematic review based on the RE-AIM framework. BMC Nursing. 2022;21(1):1-17. https://doi.org/10.1186/s12912-022-00842-0.

60. Bölsch-Peterka R, Thielmann B, Nübling M, Böckelmann I. Befragung von Beschäftigten in der ambulanten Pflege zu psychischen Belastungsfolgen mithilfe des COPSOQs – ein Altersgruppenvergleich. Zentralbl Arbeitsmed Arbeitsschutz Ergon. 2025. https://doi.org/10.1007/s40664-024-00558-9.

61. Tolksdorf K, Loenenbach A, Buda S. Dritte Aktualisierung der „Retrospektiven Phaseneinteilung der COVID-19-Pandemie in Deutschland". Epid Bull 2022;38:3-6. https://dx.doi.org/10.25646/10598.

62. Helaß M, Maatouk I. An estimate of burnout prevalence among oncology nurses. BMC Nurs. 2024;23(1):1-8. https://doi.org/10.1186/s12912-024-02421-x.

63. Koskinen S, Brugnolli A, Fuster-Linares P, Hourican S, Istomina N, Leino-Kilpi H, et al. A successful nursing education promotes newly graduated nurses' job satisfaction one year after graduation: a cross-sectional multi-country study. BMC Nurs. 2023;22(1):1-10. <https://doi.org/10.1186/s12912-023-01438-y>.

64. Heuel L, Lübstorf S, Otto A-K, Wollesen B. Chronic stress, behavioral tendencies, and determinants of health behaviors in nurses: a mixed-methods approach. BMC Public Health. 2022;22(1):1-13. https://doi.org/10.1186/s12889-022-12993-5.

65. Roth C, Berger S, Krug K, Mahler C, Wensing M. Internationally trained nurses and host nurses' perceptions of safety culture, work-life-balance, burnout, and job demand during workplace integration: a cross-sectional study. BMC Nurs. 2021;20(1):1-15. https://doi.org/10.1186/s12912-021-00581-8.

66. Möckel L, Hönl A-K, Gräfe S, Jantz F, Werner NS. Häufigkeit von Schlafproblemen bei Intensivpflegenden: Eine Post-hoc-Analyse einer Querschnittstudie. Zentralbl Arbeitsmed Arbeitsschutz Ergon. 2022;72(4):175-82. https://doi.org/10.1007/s40664-022-00466-w.

67. Hönl A-K, Jantz F, Möckel L. Schmerzen, Schmerzmitteleinnahme und mentale Gesundheit von Intensivpflegenden in Deutschland. Med Klin Intensivmed Notfmed. 2023;118(1):45-53. https://dx.doi.org/10.1007/s00063-021-00880-7.

68. Bruyneel A, Dello S, Dauvergne JE, Kohnen D, Sermeus W. Prevalence and risk factors for burnout, missed nursing care, and intention-to-leave the job among intensive care unit and general ward nurses: A cross-sectional study across six European countries in the COVID-19 era. Intensive Crit Care Nurs. 2025;86. https://doi.org/10.1016/j.iccn.2024.103885.

69. Laferton JAC, Schiller S, Conrad D, Fischer D, Zimmermann‐Viehoff F. Stress beliefs moderate the impact of COVID‐19 related work stress on depressive, anxiety and distress symptoms in health care workers. Stress Health. 2024;40(4):1-8. <https://doi.org/10.1002/smi.3410>.

70. Walter N, Wimalan B, Baertl S, Lang S, Hinterberger T, Alt V, et al. Managing periprosthetic joint infection—a qualitative analysis of nursing staffs' experiences. BMC Nurs. 2022;21(1):1-8. https://doi.org/10.1186/s12912-022-00978-z.

71. Özkaytan Y, Kukla H, Schulz-Nieswandt F, Zank S. We need a radical change to take place now´–The potential of integrated healthcare for rural long-term care facilities. Geriatr Nurs. 2024;56(null):270-7. https://doi.org/10.1016/j.gerinurse.2024.02.022.

72. Petersen J, Melzer M. Predictors and consequences of moral distress in home-care nursing: A cross-sectional survey. Nursing Ethics. 2023;30(7/8):1199-216. https://doi.org/10.1177/09697330231164761.

73. Hower KI, Pfaff H, Pförtner T-K. Pflege in Zeiten von COVID-19: Onlinebefragung von Leitungskräften zu Herausforderungen, Belastungen und Bewältigungsstrategien. Pflege. 2020;33(4):207-18. https://dx.doi.org/10.1024/1012-5302/a000752.

74. Eggert S, Teubner C. Die SARS-Cov-2 Pandemie in der professionellen Pflege: Perspektive stationärer Langzeitpflege und ambulanter Dienste. 2021. <https://doi.org/10.71059/LHWS9319>.

75. Bartzik M, Aust F, Peifer C. Negative effects of the COVID-19 pandemic on nurses can be buffered by a sense of humor and appreciation. BMC Nurs. 2021;20(1):1-12. https://doi.org/10.1186/s12912-021-00770-5.

76. Morawa E, Schug C, Geiser F, Beschoner P, Jerg-Bretzke L, Albus C, et al. Psychosocial burden and working conditions during the COVID-19 pandemic in Germany: The VOICE survey among 3678 health care workers in hospitals. J Psychosom Res. 2021;144. https://doi.org/10.1016/j.jpsychores.2021.110415.

77. Kirmse KA, Pietrzyk U, Hacker W, Saifoulline R, Fuchs K, Haubold A-K. Status Quo Pflege–Aktuelle Belastungs-und Beanspruchungs-situation in den Bereichen der Akut-und Langzeitpflege. Psychologie des Alltagshandelns-Psychologie of Everyday Activity. 2021;14(1):9-19. http://www.allgemeine-psychologie.info/wp/wp-content/uploads/2023/03/02_Kirmse.pdf. Accessed 22 Apr 2025.

78. Skoda E-M, Teufel M, Stang A, Jöckel K-H, Junne F, Weismüller B, et al. Psychological burden of healthcare professionals in Germany during the acute phase of the COVID-19 pandemic: differences and similarities in the international context. Journal of Public Health. 2020;42(4):688-95. https://doi.org/10.1093/pubmed/fdaa124.

79. Zerbini G, Ebigbo A, Reicherts P, Kunz M, Messman H. Psychosocial burden of healthcare professionals in times of COVID-19 - a survey conducted at the University Hospital Augsburg. Ger Med Sci. 2020;18:1-9. https://doi.org/10.3205/000281.

80. Hower KI, Pfaff H, Pförtner TK. Is time a healer? Course of demands during the COVID-19 pandemic in long-term care: a repeated cross-sectional survey in Germany. J Public Health (Oxf). 2021;43(3):e435-e7. https://doi.org/10.1093/pubmed/fdab144.

81. Schaps V, Hower KI, Pfaff H, Pförtner T-K. Irritation, General and Pandemic-Related Demands, and the Importance of Workplace Health Promotion in Long-Term Care—Results From a Survey of Managers in Outpatient and Inpatient Long-Term Care Facilities in Germany. J Occup Environ Med. 2024;66(2):148-55. https://doi.org/10.1097/JOM.0000000000003011.

82. Mai T, Todisco L, Schilder M, Franke V, Ristau J. Die Situation der Pflegenden in Akutkrankenhäusern während der zweiten Welle der COVID-19-Pandemie: Eine Onlinebefragung. Pflege. 2022;35(2):104-13. <https://doi.org/10.1024/1012-5302/a000846>.

83. Bauer J, Kocks A, Luboeinski J, Fischer U. Bewährungsprobe Coronapandemie. Pflegezeitschrift. 2021;74(5):52-5. https://doi.org/10.1007/s41906-021-1021-y.

84. Dürr L, Forster A, Bartsch CE, Koob C. Anforderungen, Ressourcen und Arbeitsengagement Pflegender während der zweiten Welle der COVID-19-Pandemie: Eine Querschnittstudie. Pflege. 2022;35(1):5-14. <https://doi.org/10.1024/1012-5302/a000820>.

85. Hering C, Gangnus A, Budnick A, Kohl R, Steinhagen-Thiessen E, Kuhlmey A, et al. Psychosocial burden and associated factors among nurses in care homes during the COVID-19 pandemic: findings from a retrospective survey in Germany. BMC Nurs. 2022;21(1):1-10. https://doi.org/10.1186/s12912-022-00807-3.

86. Lücker P, Henning E, Kästner A, Hoffmann W. Inactive nurses' willingness to return to active nursing during the COVID-19 pandemic: A qualitative study. J Adv Nurs. 2024;80(3):1043-57. https://doi.org/10.1111/jan.15881.

87. Kuhlmann E, Behrens GMN, Cossmann A, Homann S, Happle C, Dopfer-Jablonka A. Healthcare workers’ perceptions and medically approved COVID-19 infection risk: understanding the mental health dimension of the pandemic. A German hospital case study. medRxiv. 2022. https://doi.org/10.1101/2022.03.28.22273029.

88. Bußmann A, Pomorin N. Psychosocial burdens in palliative care – a longitudinal cohort study in nursing homes and impacts of the COVID-19 pandemic. BMC Palliat Care. 2023;null(null):1-12. https://doi.org/10.1186/s12904-023-01292-4.

89. Winnand P, Fait Y, Ooms M, Bock A, Heitzer M, Laurentius T, et al. Assessment of psychological and physical stressors among nurses in different functional areas before and during the COVID-19 pandemic: a cross-sectional study. BMC Nurs. 2023;22(1):1-11. <https://doi.org/10.1186/s12912-023-01424-4>.

90. Schulze S, Merz S, Thier A, Tallarek M, König F, Uhlenbrock G, et al. Psychosocial burden in nurses working in nursing homes during the Covid-19 pandemic: a cross-sectional study with quantitative and qualitative data. BMC Health Serv Res. 2022;22(1):1-7. https://doi.org/10.1186/s12913-022-08333-3.

91. Krieger H, Rhein C, Morawa E, Adler W, Steffan J, Lang-Richter N, et al. Using Heart Rate Variability to Assess Nurses' Stress During the COVID-19 Pandemic. West J Nurs Res. 2024;46(7):492-500. https://doi.org/10.1177/01939459241252078.

92. Wildgruber D, Frey J, Seer M, Pinther K, Koob C, Reuschenbach B. Arbeitsengagement und Belastungserleben von Health Professionals in Zeiten der Corona-Pandemie: Eine Querschnittstudie. Pflege. 2020;33(5):299-307. https://doi.org/10.1024/1012-5302/a000759.

93. Benzinger P, Kuru S, Keilhauer A, Hoch J, Prestel P, Bauer JM, et al. Psychosocial effects of the pandemic on staff and residents of nursing homes as well as their relatives-A systematic review. Z Gerontol Geriatr. 2021;54(2):141-5. <https://doi.org/10.1007/s00391-021-01859-x>.

94. Sporket M. Traurig und verzweifelt, aber verständnisvoll–Ergebnisse der Befragung zur Situation in Pflegeheimen. Sozialr Prax. 2020;30(12):1-8.

95. Arndt D, Hering T. Workload and mental health of nursing staff in Germany during the COVID-19 pandemic-a scoping review. Bundesgesundheitsblatt Gesundheitsforschung Gesundheitsschutz. 2025;68(2):130-40. https://doi.org/10.1007/s00103-024-03984-5.

96. Engelmann P, Toussaint A, Addo MM, Brehm TT, Lohse AW, Weigel A, et al. Predictors of somatic symptom burden in healthcare professionals during the COVID-19 pandemic: an 8-week follow-up study. Journal of Mental Health. 2023;32(6):1111-21. https://doi.org/10.1080/09638237.2022.2069709.
